# Chemical Characterization, Antioxidant and Enzyme‐Inhibitory Activities of Different Extracts from Three *Phlomis* Species

**DOI:** 10.1002/open.202500004

**Published:** 2025-05-12

**Authors:** Sakina Yagi, Gokhan Zengin, Mehmet Veysi Cetiz, Zoltán Cziáky, József Jeko, Muammer Bahşi, Uğur Çakılcıoğlu, Halbay Turumtay, Abdurrahman Aktumsek

**Affiliations:** ^1^ Department of Botany Faculty of Science University of Khartoum Khartoum 11115 Sudan; ^2^ Department of Biology Science Faculty Selcuk University 42130 Konya Türkiye; ^3^ Department of Medical Biochemistry Faculty of Medicine Harran University 63290 Sanliurfa Turkey; ^4^ Agricultural and Molecular Research and Service Institute University of Nyíregyháza 001 Nyíregyháza HU‐4400 Hungary; ^5^ Faculty of Education Department of Primary Education Fırat University Elazığ Turkey; ^6^ Pertek Sakine Genç Vocational School Munzur University 62500 Pertek,Tunceli Turkey; ^7^ Department of Energy System Engineering Karadeniz Technical University 61830 Trabzon Turkey; ^8^ Feedstock Division Joint BioEnergy Institute Emeryville CA 94608 USA; ^9^ Environmental Genomics and Systems Biology Division Lawrence Berkeley National Laboratory Berkeley CA 94720 USA

**Keywords:** antioxidants, enzyme inhibition, *P. herba‐venti*, *
P. kurdica*, *Phlomis fruticosa*, phytoconstituents

## Abstract

*Phlomis* species (family Lamiaceae) are highly valued as food and herbal medicine. The present study is designed to investigate the chemical composition and antioxidant and enzyme inhibitory activities of extracts from *P. fruticosa,* *P. herba‐venti,* and *P. kurdica* aerial parts. Different classes of metabolites, including phenolic acids, phenylethanoids, flavonoids, iridoids, organic acids, terpenes, and fatty acids, are identified in the three species, with methanol as the best solvent to recover bioactive compounds from the three species in addition to ethyl acetate for *P. kurdica*. Around 70% methanol extract of *P. herba‐venti* exerts the best radical scavenging and ions‐reducing properties, while its methanol extract exhibits the highest acetylcholinesterase inhibitory activity. The ethyl acetate extract of *P. fruticosa* displays the best chelating power, and its other polar extracts have the highest total antioxidant activity. Furthermore, molecular docking and molecular dynamics simulations have underscored the therapeutic potential of bioactive compounds, including isoverbascoside, samioside, forsythoside B, and hattushoside. In conclusion, the study indicates that these three *Phlomis* species are a rich source of bioactive molecules with possible therapeutic applications, and the selection of appropriate extraction solvents is crucial for the targeted biological activity.

## Graphical abstract

The tested *Phlomis* species are a rich source of bioactive molecules with potential therapeutic applications, and selecting appropriate extraction solvents is crucial for achieving the targeted biological activity.

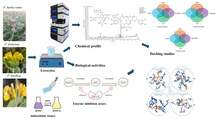

## 1 Introduction

The genus *Phlomis* belongs to the Lamiaceae (Labiatae) family and comprises about 100 perennial herbal species distributed worldwide in Europe, Asia, Africa, and North America.^[^ ^1^ ^]^ The genus has two centers of diversity; the first one includes south and east Anatolia and North‐Western Iran, and the second covers the Central Asian parts of the former Soviet Union to eastern China.^[^ 2, 3 ^]^ Phytochemical studies demonstrated that *Phlomis* species contain a diverse range of compounds like iridoids, phenylpropanoids, flavonoids, lignans, neolignans, monoterpenes, megastigmanes, diterpene glycosyl esters, nortriterpenes, and essential oils.^[^ 4, 5, 6, 7, 8 ^]^ Pharmacologically, they are shown to possess antinociceptive, anti‐inflammatory,^[^ ^9^ ^]^ antioxidant,^[^ ^10^ ^]^ antimicrobial,^[^ ^11^ ^]^ antidiabetic,^[^ ^12^ ^]^ antiviral,^[^ ^13^ ^]^ antiplasmodial,^[^ ^14^ ^]^ and anticancer^[^ ^15^ ^]^ activities, among others.

In Turkey, the genus *Phlomis* is represented by 33 species. They are divided into three groups; Group A includes species with pink or purple corolla, and Groups B and C have a yellow corolla, the former being shrubs or herbs with numerous bracteoles, the latter being herbs having few or no bracteoles.^[^ ^16^ ^]^ Most of these species, including *P. fruticosa,* *P. herba‐venti,* and *P. kurdica* are highly valued as food and herbal medicine. *P. fruticosa* is used traditionally to treat gastric ulcer, and cough and as a cicatrizant agent.^[^ ^17^ ^]^ *P. kurdica* is used as a stimulant and for respiratory diseases.^[^ ^18^ ^]^ Leaves of *P. herba‐venti* are cooked by boiling and used as food.^[^ ^19^ ^]^ Previous studies identified phenolic acids, flavonoids, and phenylpropanoids from the methanolic extract of *P. fruticosa* aerial parts, and the extract exerted antimicrobial activity and inhibited the amylase enzyme.^[^ ^8^ ^]^ Essential oils from the leaves and flowers of *P. herba‐venti* possessed antimicrobial activity.^[^ ^20^ ^]^ Essential oil was also extracted from *P. kurdica*.^[^ ^18^ ^]^ Phenylpropanoids and flavonoids isolated from the aerial parts of *P. kurdica* inhibited the human lactate dehydrogenase^[^ ^5^ ^]^ and exerted cytotoxicity against breast adenocarcinoma, hepatocellular, colon, and lung carcinoma cells.^[^ ^21^ ^]^

In the last decade, the biological activity of natural products has gained interest in the design of therapeutic applications owing to concerns about using synthetic materials.^[^ 22, 23 ^]^ For example, antioxidant compounds protect against free radical attacks associated with the progression of chronic and degenerative diseases such as cancer, diabetes, and heart disease. Several studies suggest that phytochemicals are an antioxidant treasure against this defense.^[^ ^24^ ^]^ Moreover, key enzyme inhibition is involved in treating global health problems such as Alzheimer's disease, diabetes, and obesity. In diabetic patients, for example, inhibition of amylase and glucosidase may control blood glucose levels.^[^ ^25^ ^]^ In this sense, several studies have shown that phytochemicals, especially phenols, have a high potential for inhibiting these key enzymes.^[^ ^26^ ^]^ Together, new sources of antioxidants and enzyme inhibitors are one of the most attractive topics in the scientific community.

Based on the existing literature gap and the need for an in‐depth study to explore the beneficial health potential of *Phlomis* species, the present study was designed to investigate the phytoconstituents in EtOAc, MeOH, 70% MeOH, and aqueous extracts from the aerial parts of *P. fruticosa,* *P. herba‐venti*, and *P. kurdica.* Their antioxidant activity based on their capacity to scavenge free radicals, chelate and reduce metal ions, and their ability to inhibit enzymes implicated in diabetes, skin hyperpigmentation, and Alzheimer's diseases were also evaluated. Furthermore, molecular docking and molecular dynamics (MD) simulations were employed to understand further the interactions of bioactive compounds identified in these species with key enzyme targets, such as AChE, BChE, amylase, and glucosidase. Subsequently, binding affinities, stability, and dynamic profiles of selected ligand–protein complexes were analyzed, providing insights into their potential as therapeutic agents for oxidative stress‐linked and neurodegenerative diseases.

## 2 Results and Discussion

### 2.1 Total Phenolic (TPC) and Flavonoids (TFC) Contents

Phenolic compounds abundantly found in plants have a therapeutic potential to prevent and cure diseases as they play an important role in various biological activities. The TPC and TFC on different extracts of *P. fruticosa,* *P. herba‐venti,* and *P. kurdica* aerial parts were determined, and results are presented in **Table** **1**. The TPC in extracts of the three *Phlomis* species was 22.25 and 51.82 mg GAE g^−1^, with the MeOH and 70% MeOH extracts of *P. herba‐venti* recorded the highest values. This was followed by the MeOH and 70% MeOH extracts of *P. kurdica*, 70% MeOH extracts of *P. fruticosa,* and aqueous extract of *P. herba‐venti,* which exerted comparable TPC (38.91–40.48 mg GAE g^−1^, *p* ≥ 0.05). The lowest TPC in each species was recovered in the EtOAc extracts. The TFC in the three species was in the range of 1.88–32.36 mg RE g^−1^, with the highest amount accumulated in the MeOH extract of *P. herba‐venti* followed by its 70% MeOH extract and the MeOH extract of *P. kurdica* (24.48 and 25.03 mg RE g^−1^ respectively, *p* ≥ 0.05). Although the EtOAc of *P. fruticosa* had the least TPC, it recovered the highest TFC among its 4 extracts (21.36 mg RE g^−1^), indicating that the major constituent was flavonoids. A previous study determining the TPC in 80% MeOH extracts of aerial parts of *P. herba‐venti* and *P. kurdica* growing in Iran found that the former species had lower values (TPC = 22.14 mg GAE g^−1^) than the latter one (TPC = 24.24 mg GAE g^−1^) contrary to the results obtained for the MeOH, 70% MeOH and aqueous extracts in the present study.^[^ ^27^ ^]^ Another study on the aerial parts of *P. herba‐venti* grown in Turkey showed that the MeOH extract recorded comparable TPC (47.96 mg GAE g^−1^) with the results in the current study.^[^ ^28^ ^]^ This variation could be attributed to genetic and environmental factors and other parameters such as harvest time and extraction techniques.^[^ ^29^ ^]^

**Table 1:** Total phenolic and flavonoid contents in the extracts of Phlomis species aerial parts.

Species	Extracts	TPC [mg GAE g^−1^]*	TFC [mg RE g^−1^]*
*P. fruticosa*	Ethyl acetate	23.60 ± 0.35	21.36 ± 0.45
	Methanol	35.99 ± 0.30	20.05 ± 0.55
	Methanol/Water (70%)	38.96 ± 0.71	7.70 ± 0.24
	Water	34.51 ± 0.52	3.45 ± 0.11
*P. herba‐venti*	Ethyl acetate	30.60 ± 0.49	1.88 ± 0.67
	Methanol	46.07 ± 1.59	32.36 ± 0.44
	Methanol/Water (70%)	51.82 ± 1.17	24.48 ± 0.65
	Water	38.91 ± 0.68	18.82 ± 0.14
*P. kurdica*	Ethyl acetate	22.25 ± 0.10	17.83 ± 0.35
	Methanol	40.48 ± 0.64	25.03 ± 0.21
	Methanol/Water (70%)	40.10 ± 0.21	17.99 ± 0.11
	Water	31.87 ± 0.17	10.04 ± 0.04

* Values are reported as mean ± SD of three parallel measurements. GAE: Gallic acid equivalents; RE: Rutin equivalents. Different letters indicate significant differences between the tested extracts (*p* < 0.05).

### 2.2 Chemical Characterization of Extracts

Analysis of the phenolic profile of the different extracts from the three investigated *Phlomis* species was performed using the LC‐MS/MS technique, and the results are presented in **Table** **2**,**3**–**4**. In addition, the details of identified compounds are given in supplemental materials (Table S1–S12, Supporting Information). About 100 compounds were identified in the extracts of *P. fruticosa* and distributed as follows; 68 compounds in EtOAc; 85 in MeOH; 71 in 70% MeOH and 48 in aqueous extracts. From *P. herba‐venti* extracts, a total of 98 compounds were identified and distributed as follows: 70 in EtOAc; 89 in MeOH; 86 in 70% MeOH, and 72 in aqueous extracts, while the 100 identified compounds in *P. kurdica* extracts were distributed as follows; 93 in EtOAc; 93 in MeOH; 63 in 70% MeOH and 60 in aqueous extracts. Thus, MeOH was the best solvent for recovering bioactive compounds from the three species, in addition to EtOAc for *P. kurdica*. The extracts of the three species identified free phenolic acids, pentose, and hexose glycosides of the phenolic acids and their esters with quinic acid. Phenylethanoids glycosides and flavonoids and their glycosylated derivatives were also identified in addition to organic acids, iridoids, terpenes, and fatty acids. Most of these compounds were previously identified in many *Phlomis* species.^[^ 8, 16, 17 ^]^ It was reported that the flavonoid glycosides 7‐O‐glucosides, 7‐O‐glucuronides and 7‐rutinoside of apigenin, luteolin and chrysoeriol, as well as the flavone C‐glycoside vicenin‐2 and the flavanones, naringenin and eriodictyol were commonly found in *Phlomis* species and species belong to the family Lamiaceae.^[^ ^17^ ^]^ Also, phenylethylalcohol glycosides, like verbascoside and forsythoside B were identified in the three species. In contrast, tricin‐7‐O‐glucoside, which was suggested as uncommon flavone glycoside in this family, was previously identified in *P. fruticosa*, and was also detected in the present study in extracts of *P. herba‐venti*. Furthermore, iridoids were only detected in *P. fruticosa* and *P. herba‐venti* extracts. The latter species, in addition to *P. kurdica* extracts, showed the presence of N1,N5,N10‐tricoumaroylspermidines, and to the best of our knowledge, this is the first report of the presence of spermidine hydroxycinnamic acid in *Phlomis* species.

**Table 2:** Chemical composition of *P. fruticosa* extracts.

	Compounds	EA	MeOH	MeOH/Water	Water
1	Quinic acid	+	+	+	+
2	Citric acid	–	+	+	+
3	Gallic acid (3,4,5‐Trihydroxybenzoic acid)	–	+	+	–
4	Protocatechuic acid (3,4‐Dihydroxybenzoic acid)	–	+	+	+
5	p‐Hydroxybenzoic acid	–	+	–	–
6	Lamiide	–	+	+	+
7	Neochlorogenic acid (5‐O‐Caffeoylquinic acid)	+	+	+	+
8	Vanillic acid‐4‐O‐pentosylhexoside	–	+	+	+
9	Decaffeoylverbascoside	+	+	+	+
10	Genipin or isomer	+	–	–	–
11	Uralenneoside	+	+	+	+
12	2‐Isopropylmalic acid	–	+	+	+
13	3‐O‐(p‐Coumaroyl)quinic acid cis isomer	–	+	–	+
14	Swertiamacroside	–	+	+	+
15	Unidentified iridoid	–	+	+	+
16	3‐O‐(p‐Coumaroyl)quinic acid	–	+	–	+
17	Vanillic acid (4‐Hydroxy‐3‐methoxybenzoic acid)	–	+	–	–
18	Caffeic acid‐O‐hexoside	–	+	+	+
19	Chlorogenic acid (3‐O‐Caffeoylquinic acid)	+	+	+	+
20	Caffeic acid	+	+	+	+
21	Genipin or isomer	+	+	+	+
22	Chryptochlorogenic acid (4‐O‐Caffeoylquinic acid)	+	+	–	+
23	4‐O‐(p‐Coumaroyl)quinic acid cis isomer	–	–	–	+
24	5‐O‐(p‐Coumaroyl)quinic acid	+	–	–	+
25	Syringic acid	–	+	–	–
26	5‐O‐(p‐Coumaroyl)quinic acid	–	+	–	+
27	p‐Coumaric acid	+	+	+	+
28	Vicenin‐2 (Apigenin‐6,8‐di‐C‐glucoside)	–	+	+	+
29	Campneoside II (β‐Hydroxverbascoside)	+	+	+	+
30	5‐O‐(p‐Coumaroyl)quinic acid cis isomer	+	+	–	+
31	Ferulic acid	+	+	–	+
32	Campneoside II (β‐Hydroxverbascoside) isomer 1	–	–	–	+
33	Campneoside II (β‐Hydroxverbascoside) isomer 2	–	–	–	+
34	Eriodictyol‐O‐hexoside	+	+	+	–
35	Pentahydroxy(iso)flavone‐O‐hexoside	+	+	+	+
36	Forsythoside B	+	+	+	+
37	Luteolin‐O‐hexosylhexoside	–	+	–	+
38	Verbascoside (Acteoside)	–	+	+	+
39	Luteolin‐O‐pentosylhexoside	+	+	+	+
40	Dicaffeoylquinic acid isomer 1	–	–	+	+
41	Prunin (Naringenin‐7‐O‐glucoside)	+	+	+	–
42	Luteolin‐O‐rhamnosylhexoside	+	+	+	+
43	Luteolin‐7‐O‐glucoside (Cynaroside)	+	+	+	+
44	Unidentified syringic acid derivative	+	+	+	+
45	Dicaffeoylquinic acid isomer 2	–	+	+	–
46	Alyssonoside	+	+	+	+
47	Isoverbascoside	+	+	+	+
48	Isoquercitrin (Quercetin‐3‐O‐glucoside)	+	+	+	–
49	Leucosceptoside A or Plantainoside C	+	+	+	–
50	Methoxy‐tetrahydroxy(iso)flavone‐O‐hexoside	+	+	+	+
51	Apigenin‐O‐pentosylhexoside	–	+	–	–
52	Chrysoeriol‐O‐pentosylhexoside	+	+	+	+
53	Apigenin‐O‐rhamnosylhexoside	+	+	+	+
54	Unidentified phenylethanoid	+	+	+	–
55	Chrysoeriol‐O‐rhamnosylhexoside	+	+	+	–
56	Cosmosiin (Apigenin‐7‐O‐glucoside)	+	+	+	–
57	Dicaffeoylquinic acid isomer 3	–	+	+	+
58	Chrysoeriol‐7‐O‐glucoside	+	+	+	–
59	Methoxy‐trihydroxy(iso)flavone‐O‐hexoside	+	+	–	–
60	Eriodictyol (3′,4′,5,7‐Tetrahydroxyflavanone)	+	+	+	–
61	Leucosceptoside A or Plantainoside C	+	+	+	–
62	Caffeoyl‐hydroxybenzoylhexose	–	+	+	+
63	Tricin‐7‐O‐glucoside	+	+	+	–
64	Isorhamnetin‐3‐O‐glucoside	+	+	+	–
65	Abscisic acid	–	+	–	–
66	Martynoside	+	+	+	–
67	Naringenin (4′,5,7‐Trihydroxyflavanone)	+	+	+	+
68	Homoeriodictyol (3′‐Methoxy‐4′,5,7‐trihydroxyflavanone)	+	+	+	–
69	Lubiminol or Canusesnol I	–	+	+	–
70	Hesperetin (4′‐Methoxy‐3′,5,7‐trihydroxyflavanone)	+	+	+	–
71	Lubiminol or Canusesnol I	–	+	+	–
72	Luteolin (3′,4′,5,7‐Tetrahydroxyflavone)	+	+	+	+
73	Luteolin‐O‐(Z‐p‐coumaroyl)glucoside	–	+	+	–
74	Apigenin‐O‐(E‐p‐coumaroyl)glucoside	+	+	+	–
75	Luteolin‐O‐(E‐p‐coumaroyl)glucoside	–	+	+	–
76	Chrysoeriol‐7‐O‐(3‐Z‐p‐coumaroyl)glucose	+	+	+	–
77	Apigenin (4′,5,7‐Trihydroxyflavone)	+	+	+	+
78	Chrysoeriol (3′‐Methoxy‐4′,5,7‐trihydroxyflavone)	+	+	+	+
79	Dimethoxy‐trihydroxy(iso)flavone	+	+	+	–
80	Chrysoeriol‐7‐O‐(3‐E‐p‐coumaroyl)glucose	+	+	+	–
81	Apigenin‐O‐(Z‐p‐coumaroyl)glucoside	+	+	+	–
82	Dihydroxy‐dimethoxy(iso)flavone isomer 1	+	+	+	–
83	Dihydroxy‐trimethoxy(iso)flavone isomer 1	+	+	+	–
84	Dihydroxy‐dimethoxy(iso)flavone isomer 2	+	+	+	–
85	Dihydroxy‐trimethoxy(iso)flavone isomer 2	+	+	+	–
86	Hydroxy‐tetramethoxy(iso)flavone	+	+	+	–
87	Hydroxy‐trimethoxy(iso)flavone isomer 1	+	+	+	–
88	Hydroxy‐trimethoxy(iso)flavone isomer 2	+	+	+	–
89	Phytosphingosine	–	+	–	–
90	Stearidonic acid	+	–	–	–
91	Hydroxyoctadecatrienoic acid	+	–	–	–
92	*cis*‐9,10‐Epoxystearic acid	+	–	–	–
93	α‐Linolenic acid	+	+	–	–
94	Linoleic acid	+	+	+	–
95	Linoleic acid isomer	+	–	–	–
96	Oleic acid	+	–	–	–
97	Stearic acid	+	–	–	–
98	Arachidic acid	+	–	–	–
99	Lignoceric acid	+	–	–	–
100	Cerotic acid	+	–	–	–

**Table 3:** Chemical composition of P. herba‐venti extracts.

	Compounds	EA	MeOH	MeOH/Water	Water
1	Quinic acid	+	+	+	+
2	Citric acid	+	+	+	+
3	Protocatechuic acid (3,4‐Dihydroxybenzoic acid)	–	+	+	+
4	Lamiide	–	+	–	+
5	Neochlorogenic acid (5‐O‐Caffeoylquinic acid)	–	–	–	+
6	Decaffeoylverbascoside	–	+	+	–
7	Unidentified iridoid	+	+	+	+
8	Unidentified iridoid	+	–	–	–
9	Chlorogenic acid (3‐O‐Caffeoylquinic acid)	+	+	+	+
10	Caffeic acid	+	+	+	+
11	Chryptochlorogenic acid (4‐O‐Caffeoylquinic acid)	–	–	–	+
12	Kynurenic acid	–	+	+	+
13	Naringenin‐6,8‐di‐C‐glucoside	–	+	+	+
14	5‐O‐(4‐Coumaroyl)quinic acid	+	+	+	+
15	12‐Hydroxyjasmonic acid or tuberonic acid	–	+	+	+
16	12‐Hydroxyjasmonic acid or tuberonic acid	–	+	+	+
17	Caffeoylshikimic acid	+	+	+	+
18	Riboflavin	–	–	+	+
19	5‐O‐Feruloylquinic acid	+	+	+	+
20	p‐Coumaric acid	+	+	+	+
21	Vicenin‐2 (Apigenin‐6,8‐di‐C‐glucoside)	–	+	+	+
22	5‐O‐(4‐Coumaroyl)quinic acid cis isomer	+	+	+	+
23	12‐Hydroxyjasmonic acid sulfate or tuberonic acid sulfate	–	+	+	+
24	5‐O‐Feruloylquinic acid cis isomer	+	+	+	+
25	Pentahydroxy(iso)flavone‐O‐rhamnosylglucuronide	+	+	+	+
26	Pentahydroxy(iso)flavone‐O‐hexoside	+	+	+	+
27	Forsythoside B	+	+	+	–
28	Luteolin‐O‐rhamnosylhexoside isomer 1	+	+	+	+
29	Verbascoside (Acteoside)	+	+	+	–
30	Luteolin‐O‐rhamnosylglucuronide isomer 1	–	+	+	+
31	Luteolin‐O‐pentosylhexoside	–	+	+	+
32	Forsythoside B isomer	+	+	+	–
33	Luteolin‐O‐rhamnosylhexoside isomer 2	–	+	+	+
34	Hattushoside	+	+	+	+
35	Luteolin‐7‐O‐glucoside (Cynaroside)	–	+	+	+
36	Luteolin‐O‐crotonylhexoside	+	+	+	+
37	Alyssonoside	+	+	+	–
38	Methoxy‐tetrahydroxy(iso)flavone‐O‐hexoside isomer 1	+	+	+	+
39	Isoverbascoside	+	+	+	–
40	Luteolin‐O‐rhamnosylhexoside isomer 3	–	+	+	+
41	Luteolin‐O‐glucuronide	–	+	–	+
42	Luteolin‐O‐rhamnosylglucuronide isomer 2	+	+	+	+
43	Leucosceptoside A	+	+	+	–
44	Methoxy‐tetrahydroxy(iso)flavone‐O‐hexoside isomer 2	+	+	+	+
45	Methoxy‐tetrahydroxy(iso)flavone‐O‐glucuronide isomer 1	–	+	+	+
46	Chrysoeriol‐O‐pentosylhexoside	–	+	+	+
47	Alyssonoside isomer	+	+	+	–
48	Methoxy‐tetrahydroxy(iso)flavone‐O‐glucuronide isomer 2	–	+	+	+
49	Cosmosiin (Apigenin‐7‐O‐glucoside)	–	+	+	+
50	Dicaffeoylquinic acid	–	+	+	–
51	Chrysoeriol‐7‐O‐glucoside	+	+	+	+
52	Forsythoside G or isomer	+	+	+	+
53	Leucosceptoside B isomer	+	+	+	+
54	Apigenin‐7‐O‐glucuronide	–	+	+	+
55	Eriodictyol (3′,4′,5,7‐Tetrahydroxyflavanone)	+	+	–	–
56	Caffeoyl‐hydroxybenzoylhexose	+	+	+	+
57	Chrysoeriol‐O‐glucuronide isomer 1	–	+	+	+
58	Tricin‐7‐O‐glucoside	+	+	+	+
59	Abscisic acid	+	+	+	+
60	Chrysoeriol‐O‐glucuronide isomer 2	–	+	+	+
61	Chrysoeriol‐O‐glucuronide isomer 3	–	+	+	+
62	Luteolin‐O‐(acetylrhamnosyl)glucuronide isomer 1	+	+	+	+
63	Methoxy‐tetrahydroxy(iso)flavone‐O‐(acetylrhamnosyl)glucuronide	+	+	+	+
64	Leucosceptoside B	+	+	+	+
65	N1,N5,N10‐Tricoumaroylspermidine isomer 1	+	+	+	+
66	Naringenin (4′,5,7‐Trihydroxyflavanone)	+	+	+	+
67	N1,N5,N10‐Tricoumaroylspermidine isomer 2	+	+	+	+
68	Lubiminol or Canusesnol I	+	+	+	–
69	Luteolin‐O‐(acetylrhamnosyl)glucuronide isomer 2	+	+	+	+
70	Dimethoxy‐trihydroxy(iso)flavone isomer 1	–	–	+	+
71	Lubiminol or Canusesnol I	+	+	+	+
72	Luteolin (3′,4′,5,7‐Tetrahydroxyflavone)	+	+	+	+
73	N1,N5,N10‐Tricoumaroylspermidine isomer 3	+	+	+	–
74	Apigenin‐O‐(acetylrhamnosyl)glucuronide	+	+	+	+
75	Chrysoeriol‐O‐(acetylrhamnosyl)glucuronide	+	+	+	–
76	N1,N5,N10‐Tricoumaroylspermidine isomer 4	+	+	+	–
77	Apigenin‐O‐coumaroylhexoside	+	+	+	–
78	ior Canusesnol I isomer	+	–	–	–
79	Chrysoeriol‐7‐O‐(3‐Z‐p‐coumaroyl)glucose	+	+	+	+
80	Dimethoxy‐trihydroxy(iso)flavone isomer 2	+	+	+	+
81	Apigenin (4′,5,7‐Trihydroxyflavone)	+	+	+	+
82	Chrysoeriol (3′‐Methoxy‐4′,5,7‐trihydroxyflavone)	+	+	+	+
83	Dimethoxy‐trihydroxy(iso)flavone isomer 3	+	+	+	+
84	Chrysoeriol‐7‐O‐(3‐E‐p‐coumaroyl)glucose	+	+	+	+
85	Traumatic acid (2‐Dodecenedioic acid)	+	+	+	–
86	Traumatic acid isomer	+	+	+	+
87	Acacetin (5,7‐Dihydroxy‐4′‐methoxyflavone)	+	+	+	+
88	9‐Hydroxyoctadecatrienoic acid	+	+	+	+
89	13‐Hydroxyoctadecatrienoic acid	+	+	+	+
90	9‐Hydroxyoctadecadienoic acid	+	+	+	+
91	Unidentified terpenoid	+	–	–	–
92	α‐Linolenic acid	+	+	+	–
93	Linoleic acid	+	+	+	–
94	Linoleic acid isomer	+	+	+	–
95	Palmitic acid	+	–	–	–
96	Oleic acid	+	–	–	–
97	Gondoic acid (*cis*‐11‐Eicosenoic acid) or isomer	+	–	–	–
98	Pheophytin A	+	+	–	–
99	–	–	–	–	–

**Table 4:** Chemical composition of *P. kurdica* extracts.

	Compounds	EA	MeOH	MeOH/Water	Water
1	Quinic acid	+	+	+	+
2	Citric acid	+	+	+	–
3	Protocatechuic acid (3,4‐Dihydroxybenzoic acid)	+	+	+	–
4	Vanillic acid‐4‐O‐glucoside	+	+	+	–
5	Hydroxybenzoic acid glucoside	–	–	+	–
6	Hydroxybenzoic acid	+	+	–	–
7	Decaffeoylverbascoside	+	+	–	–
8	Hydroxybenzoic acid glucoside	+	+	–	–
9	Vanillic acid (4‐Hydroxy‐3‐methoxybenzoic acid)	+	+	+	–
10	Chlorogenic acid (3‐O‐Caffeoylquinic acid)	+	+	+	+
11	Caffeic acid	+	+	+	+
12	Vanillin ( 4‐Hydroxy‐3‐methoxybenzaldehyde)	+	+	+	+
13	Kynurenic acid	+	+	+	–
14	Benzyl‐primeveroside or Icariside F2	+	+	–	–
15	Naringenin‐6,8‐di‐C‐glucoside	+	+	+	–
16	Ipolamiide	+	+	+	–
17	Benzyl‐primeveroside or Icariside F2	+	+	+	–
18	Syringaldehyde (3,5‐Dimethoxy‐4‐hydroxybenzaldehyde)	+	+	+	+
19	5‐O‐(p‐Coumaroyl)quinic acid	+	+	+	–
20	12‐Hydroxyjasmonic acid or Tuberonic acid	+	+	+	–
21	12‐Hydroxyjasmonic acid or Tuberonic acid	+	+	+	–
22	Riboflavin	–	–	+	–
23	Caffeoylshikimic acid	+	+	+	+
24	5‐O‐Feruloylquinic acid	+	+	+	–
25	p‐Coumaric acid	+	+	+	+
26	Vicenin‐2 (Apigenin‐6,8‐di‐C‐glucoside)	+	+	+	–
27	5‐O‐(p‐Coumaroyl)quinic acid cis isomer	+	+	+	–
28	Ferulic acid	+	+	–	+
29	N1,N10‐Bis(p‐coumaroyl)spermidine	+	+	–	–
30	Eriodictyol‐O‐hexoside	+	+	–	+
31	12‐Hydroxyjasmonic acid sulfate or Tuberonic acid sulfate	+	+	+	–
32	Apigenin‐C‐hexoside‐C‐pentoside	+	+	+	–
33	Forsythoside B	+	+	+	+
34	Luteolin‐7‐O‐sophoroside	+	+	+	–
35	Luteolin‐O‐hexosylglucuronide	–	+	+	–
36	Verbascoside (Acteoside)	+	+	–	+
37	Luteolin‐O‐rhamnosylglucuronide isomer 1	+	+	+	–
38	Luteolin‐O‐pentosylhexoside	+	+	+	–
39	Prunin (Naringenin‐7‐O‐glucoside)	+	+	–	+
40	Samioside	+	+	–	+
41	Hattushoside	+	+	+	+
42	Luteolin‐7‐O‐glucoside (Cynaroside)	+	+	+	+
43	Luteolin‐O‐crotonylhexoside	+	+	+	–
44	Alyssonoside	+	+	+	+
45	Forsythoside B isomer	+	+	–	+
46	Isoverbascoside	+	+	–	+
47	Luteolin‐O‐glucuronide	+	+	+	–
48	Luteolin‐7‐O‐(6″′‐O‐acetylglucosyl)‐(1 → 2)glucoside	+	+	+	–
49	Leucosceptoside A	+	+	–	+
50	Luteolin‐O‐rhamnosylglucuronide isomer 2	+	+	+	–
51	Caffeoyl‐vanilloylglucose	+	+	+	+
52	Chrysoeriol‐O‐pentosylhexoside	+	+	+	+
53	Alyssonoside isomer	+	+	–	+
54	Cosmosiin (Apigenin‐7‐O‐glucoside)	+	+	–	–
55	Rosmarinic acid (labiatenic acid)	+	+	+	+
56	Chrysoeriol‐7‐O‐glucoside	+	+	+	+
57	Leucosceptoside B isomer	+	+	+	+
58	Eriodictyol (3′,4′,5,7‐Tetrahydroxyflavanone)	+	+	+	+
59	Apigenin‐7‐O‐glucuronide	+	+	+	–
60	Caffeoyl‐hydroxybenzoylhexose	+	+	+	+
61	Methoxy‐tetrahydroxy(iso)flavone‐O‐glucuronide	+	+	–	–
62	Chrysoeriol‐O‐glucuronide	+	+	+	+
63	Abscisic acid	+	+	–	+
64	Chrysoeriol‐O‐(acetylglucosyl)glucuronide	+	+	+	–
65	Martynoside	+	+	+	+
66	Leucosceptoside B	+	+	+	+
67	N1,N5,N10‐Tricoumaroylspermidine isomer 1	+	+	+	+
68	Naringenin (4′,5,7‐Trihydroxyflavanone)	+	+	+	+
69	Homoeriodictyol (3′‐Methoxy‐4′,5,7‐trihydroxyflavanone)	+	+	+	–
70	N1,N5,N10‐Tricoumaroylspermidine isomer 2	+	+	+	+
71	Luteolin‐O‐(acetylrhamnosyl)glucuronide	+	+	+	–
72	N1,N5,N10‐Tricoumaroylspermidine isomer 3	+	+	+	+
73	Luteolin (3′,4′,5,7‐Tetrahydroxyflavone)	+	+	+	+
74	Luteolin‐O‐(p‐coumaroyl)hexoside	+	+	–	–
75	Rosmanol isomer	+	+	–	+
76	Apigenin‐O‐(acetylrhamnosyl)glucuronide	+	+	+	–
77	Chrysoeriol‐O‐(acetylrhamnosyl)glucuronide	+	+	+	+
78	N1,N5,N10‐Tricoumaroylspermidine isomer 4	+	+	+	+
79	Apigenin‐O‐(p‐coumaroyl)hexoside	–	+	–	–
80	Apigenin‐O‐coumaroylhexoside	+	–	–	+
81	Rosmanol	+	+	–	+
82	Chrysoeriol‐O‐(p‐coumaroyl)hexoside	+	+	+	+
83	Dimethoxy‐trihydroxy(iso)flavone isomer 1	+	+	–	+
84	Apigenin (4′,5,7‐Trihydroxyflavone)	+	+	+	+
85	Chrysoeriol (3′‐Methoxy‐4′,5,7‐trihydroxyflavone)	+	+	+	+
86	Dimethoxy‐trihydroxy(iso)flavone isomer 2	+	+	+	+
87	Traumatic acid (2‐Dodecenedioic acid)	+	+	–	+
88	Traumatic acid isomer	+	+	–	+
89	Rosmanol isomer	+	+	–	+
90	Dihydroxy‐methoxy(iso)flavone	+	+	–	+
91	Hydroxy‐trimethoxy(iso)flavone	+	+	–	+
92	9‐Hydroxyoctadecatrienoic acid	+	+	–	+
93	Stearidonic acid	–	–	–	+
94	13‐Hydroxyoctadecatrienoic acid	+	+	–	+
95	9‐Hydroxyoctadecadienoic acid	+	+	–	+
96	α‐Linolenic acid	+	+	–	+
97	Linoleic acid	+	+	–	+
98	Oleic acid	–	–	–	+
99	Stearic acid	–	–	–	+
100	Pheophytin A	+	–	–	+

**Figure 1:**
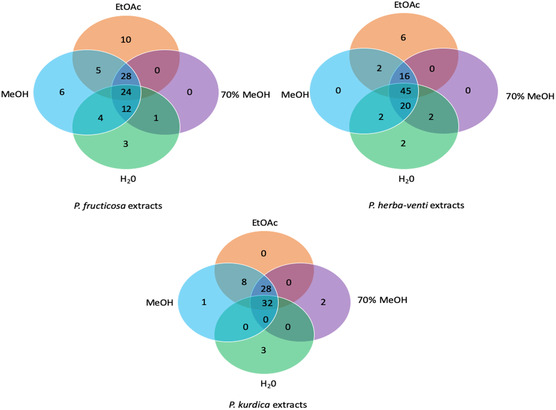
Venn diagrams showing the number and distribution of compounds in extracts of the investigated three *Phlomis* species.

**Figure 2:**
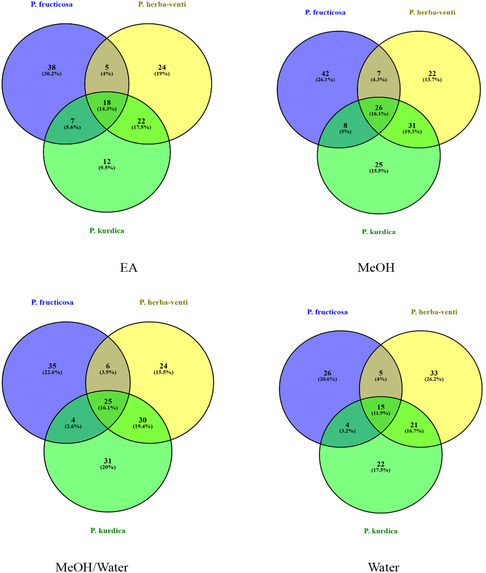
Venn diagrams comparing the number and similar compounds identified in the same extracts of the three *Phlomis* species.

**Figure 3:**
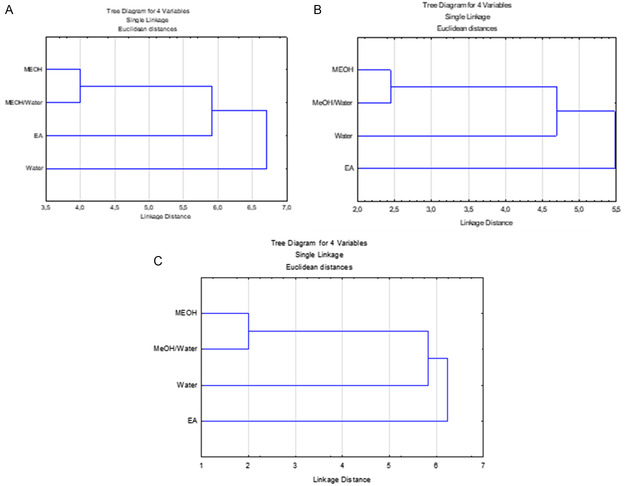
Dendrogram based on the identified compounds in the extracts of the tested *Phlomis* species. A) *P. fruticosa*, B) *P. herba‐venti*; and C) *P. kurdica*.

The distribution of the detected compounds among different extracts of the same species or in the corresponding extracts of the other two species are shown in Venn diagrams (**Figure** **1**, **2**). It was clear that *P. herba‐venti* (45) and *P. kurdica* (32) shared the highest number of similar compounds recovered in their four extracts, probably in variable amounts, while the three organic solvents extracts of *P. fruticosa* and *P. kurdica* shared the highest number of similar compounds (28). Besides, EtOAc (10) and MeOH (6) of *P. fruticosa* had the highest number of distinct compounds not identified in the other extracts. Furthermore, the MeOH and 70% MeOH extracts were similar in the tested *Phlomis* species, and we obtained a dendrogram for each species based on the identified compounds in the extracts (**Figure** **3**). Moreover, as shown in Figure 2, there are many compounds in common between the three species with the highest number (24) recovered from the MeOH extract; however, it was evident that each species had their characteristic compounds indicating their distinct identity. Indeed, this variation would reflect on the biological activities of each species. It is worth mentioning that the present study represented the first comprehensive chemical profile of *P. herba‐venti* and *P. kurdica*.

### 2.3 Antioxidant Activity

Antioxidant compounds are crucial against the harmful effects of free radicals that contribute to developing serious diseases such as cancer and diabetes. Consequently, exploration of novel and potent sources of antioxidants is of utmost importance. In line with this objective, the antioxidant properties of the three *Phlomis* species aerial parts extracts were assessed using various assays. DPPH and ATBS assays measure the ability of the extract to scavenge free radicals, and FRAP and CUPRAC assays indicate the ions reducing the capacity of the extract. In contrast, the phosphomolybdenum assay evaluates its effectiveness in reducing the Mo(VI) to Mo (V). The metal chelating ability of extract is determined by measuring its iron chelating ability. The antioxidant activity of the three *Phlomis* species in the six assays varied according to the species and type of extract (**Table** **5**). 70% MeOH extract of *P. herba‐venti* displayed significantly (*p* < 0.05) the highest antiradical (DPPH = 90.70 mg TE g^−1^, ABTS = 117.69 mg TE g^−1^) and ions reducing (CUPRC = 242.01 mg TE g^−1^, FRAP =128.96 mg TE g^−1^) properties followed by its MeOH extract. In contrast, the EtOAc extract of *P. fruticosa* exerted the best chelating power (28.93 mg EDTAE g^−1^), and its three other extracts (MeOH, 70% MeOH, and aqueous) exhibited the best total antioxidant activity (2.35–2.48 mmol TE g^−1^, *p* ≥ 0.05). Among the *P. kurdica* extracts, the MeOH and 70% MeOH extracts recorded the best antiradical and ion‐reducing properties with values mainly comparable to some polar extracts from *P. fruticosa.* In contrast, its EtOAc extract displayed the second‐best chelating power (27.25 EDTAE g^−1^). Globally, the results of antiradical and ion‐reducing properties align with the TPC values, with 70% of MeOH and MeOH extracts of *P. herba‐venti* recording the highest values. A previous study showed that 80% MeOH extract of *P. kurdica* aerial parts exerted higher anti‐DPPH (48.97 mg ascorbic acid equivalent (AAE) g^−1^) and Fe^3+^ ion reducing (28.8 μmol Fe2+ g^−1^) ability than *P. herba‐venti* extract (DPPH assay = 36.25 mg AAE g^−1^; FRAP = 15.62 μmol Fe2+ g^−1^).^[^ ^27^ ^]^ In contrast, results of a previous study on the antioxidant activity of MeOH extract of *P. fruticosa* aerial parts showed lower values in the DPPH (39.30 mg TE g^−1^), ABTS (54.62 mg TE/g) and phosphomolybdenum (1.22 mmol TE g^−1^) assays but higher effect was obtained from the FRAP (71.17 mg TE g^−1^), CUPRAC (123.44 mg TE g^−1^), and metal chelating (13.16 EDTAE g^−1^) assays than those recorded in the present study.^[^ ^8^ ^]^ As mentioned before, these variations may be attributed to genetic and environmental factors as well as a method of extraction.^[^ ^29^ ^]^ Overall, the antioxidant activity of these three *Phlomis* species could be mainly attributed to the presence of phenolic acids (such as gallic, protocatechuic, caffeic, ferulic, and p‐coumaric acids) and flavonoids (like apigenin, luteolin and chrysoeriol and their glycosides) in different extracts.^[^ 30, 31 ^]^ Also, the phenylethanoids forsythoside B and verbascoside (Acteoside), identified in the three species, exert significant anti‐DPPH activity.^[^ 32, 33 ^]^ Furthermore, it was proposed that the antioxidative activity of phenylethanoid glycosides is related to the number of phenolic hydroxyl, steric hindrance, and *ortho*‐dihydroxyphenyl group.^[^ ^33^ ^]^

**Table 5:** Antioxidant properties of the extracts of Phlomis species aerial parts.

Species	Extracts	DPPH [mg TE g^−1^]*	ABTS [mg TE g^−1^]*	CUPRAC [mg TE g^−1^]*	FRAP [mg TE g^−1^]*	Chelating [mg EDTAE g^−1^]*	PBD [mmol TE g^−1^]*
*P. fruticosa*	Ethyl acetate	8.90 ± 0.96	27.07 ± 2.20	60.70 ± 1.77	29.00 ± 1.60	28.93 ± 1.92	1.83 ± 0.07
	Methanol	50.40 ± 0.89	69.40 ± 0.59	107.58 ± 0.90	60.96 ± 1.84	12.26 ± 1.17	2.48 ± 0.12
	Methanol/Water (70%)	51.29 ± 0.66	80.68 ± 0.64	147.75 ± 4.55	77.47 ± 1.59	20.12 ± 2.89	2.38 ± 0.10
	Water	44.58 ± 3.34	79.04 ± 1.08	102.08 ± 1.76	66.06 ± 0.63	15.63 ± 1.32	2.35 ± 0.14
*P. herba‐venti*	Ethyl acetate	14.05 ± 1.04	26.15 ± 0.40	60.66 ± 3.34	31.39 ± 0.75	21.09 ± 1.27	2.06 ± 0.12
	Methanol	53.18 ± 0.14	86.59 ± 1.16	190.83 ± 8.94	95.01 ± 0.34	9.86 ± 1.30	1.83 ± 0.04
	Methanol/Water (70%)	96.70 ± 3.22	117.69 ± 1.94	242.01 ± 4.67	128.06 ± 1.62	24.81 ± 1.24	1.81 ± 0.07
	Water	46.17 ± 2.06	76.94 ± 0.17	131.70 ± 3.86	67.88 ± 0.37	19.89 ± 1.10	1.59 ± 0.04
*P. kurdica*	Ethyl acetate	3.27 ± 0.09	11.80 ± 0.61	58.43 ± 0.60	23.81 ± 0.27	27.25 ± 1.45	1.64 ± 0.05
	Methanol	51.37 ± 0.62	78.78 ± 0.23	146.77 ± 8.13	78.00 ± 0.51	13.59 ± 1.65	1.32 ± 0.08
	Methanol/Water (70%)	51.60 ± 0.59	78.92 ± 0.25	135.20 ± 0.94	80.49 ± 6.60	19.03 ± 4.67	1.14 ± 0.03
	Water	39.79 ± 3.39	66.39 ± 0.58	85.67 ± 1.95	54.62 ± 1.03	15.20 ± 2.28	0.91 ± 0.05

* Values are reported as mean ± SD of three parallel measurements. PBD: phosphomolybdenum; MCA: metal chelating activity; TE: trolox equivalent; EDTAE: EDTA equivalent. Different letters indicate significant differences between the tested extracts (*p* < 0.05).

### 2.4 Enzyme Inhibitory Activity

Studies have demonstrated that natural substances from plants that can inhibit enzymes play a potential therapeutic role in treating several diseases. For example, cholinesterase inhibitors like acetylcholinesterase (AChE), which increases the synaptic acetylcholine level, and butyrylcholinesterase (BChE), which reduces the beta‐amyloid protein level, are effective for the treatment of Alzheimer's disease. Inhibition of enzymes associated with type 2‐diabetes, like α‐glucosidase and α‐amylase, regulates the blood sugar level and hence offers an attractive strategy to control postprandial hyperglycemia. Tyrosinase (Tyr) inhibitors play an important role as depigmentation agents in cosmetics and medicinal industries and as anti‐browning compounds in the food and agriculture industries.^[^ ^34^ ^]^ Hence, the present study investigated the capacity of the three *Phlomis* species aerial parts extracts to inhibit the AchE, BchE, tyrosinase, α‐amylase, and α‐glucosidase enzymes. Results are shown in **Table** **6**. MeOH extracts of *P. herba‐venti* and *P. kurdica* showed the highest anti‐AChE (1.91 and 1.81 mg GALAE g^−1^, *p* ≥ 0.05) followed by the EtOAc extract of the former and MeOH extract of *P. fruticosa* (1.72 and 1.71 mg GALAE g^−1^, *p* ≥ 0.05). Moreover, the EtOAc extracts of the three investigated *Phlomis* species, in addition to the MeOH extract of *P. herba‐venti* exerted potent anti‐BChE activity (5.12–6.96 mg GALAE g^−1^) with EtOAc extract of *P. kurdica* displayed the highest significant (*p* < 0.05) effect. Concerning the inhibitory effect of extracts toward Tyr, it was noted that all extracts of the three *Phlomis* species (except their aqueous ones) had comparable anti‐Tyr activity (38.27–43.57 mg KAE g^−1^, *p* ≥ 0.05). Generally, the studied species were less effective toward the two enzymes associated with diabetes, and the highest inhibition of α‐glucosidase and α‐amylase was obtained respectively from the EtOAc (0.51 mmol ACAE g^−1^) and 70% MeOH (0.44 mmol ACAE g^−1^) extracts of *P. herba‐venti*. Comparing these results with previous studies, it was observed that the MeOH extract of *P. fruticosa* aerial parts revealed higher inhibitory effect against AChE (3.45 mg GALAE g^−1^), BChE (3.34 mg GALAE g^−1^), Try (133.16 mg KAE g^−1^), and amylase (0.69 mmol ACAE g^−1^) than those obtained in the present study for the same extract of the species.^[^ ^8^ ^]^ Another study on *P. kurdica* revealed that the essential oil at a concentration 250 μg mL^−1^ exhibited moderate anti‐AChE (31.42%) and BChE (26.21%) activities.^[^ ^18^ ^]^ Furthermore, some of the identified phenolics in the present study, like rosmarinic, caffeic, and chlorogenic acids, and verbascoside (acteoside), possess significant anti‐AChE activity.^[^ 35, 36 ^]^ Also, forsythoside B exerts higher anti‐BChE activity than pure galantamine.^[^ ^37^ ^]^ The extracts' anti‐Tyr effect could be associated with their phenolic composition.^[^ ^34^ ^]^ p‐coumaric acid^[^ ^38^ ^]^ and caffeic acid^[^ ^39^ ^]^ are potent Tyr inhibitors. The three species demonstrated good enzyme inhibition, especially against the butyrylcholinesterase and tyrosinase enzymes. Furthermore, the present study reported the enzyme inhibitory property of *P. herba‐venti* for the first time, and results showed that it recorded the highest values in the majority of assays (4/5); hence, it could be suggested as a promising source of enzyme inhibitors.

**Table 6:** Enzyme inhibitory properties of the extracts of Phlomis species aerial parts.

Species	Extracts	AChE [mg GALAE g^−1^]*	BChE [mg GALAE g^−1^]*	Tyrosinase [mg KAE g^−1^]*	Amylase [mmol ACAE g^−1^]*	Glucosidase [mmol ACAE g^−1^]*
*P. fruticosa*	Ethyl acetate	1.29 ± 0.03	5.80 ± 0.96	43.55 ± 0.64	0.47 ± 0.01	0.11 ± 0.01
	Methanol	1.71 ± 0.16	na	39.08 ± 1.01	0.21 ± 0.01	0.18 ± 0.01
	Methanol/Water (70%)	1.41 ± 0.03	1.42 ± 0.08	43.57 ± 0.34	0.14 ± 0.01	0.20 ± 0.01
	Water	0.79 ± 0.10	1.60 ± 0.07	15.60 ± 2.02	0.05 ± 0.01	0.13 ± 0.01
*P. herba‐venti*	Ethyl acetate	1.72 ± 0.02	5.19 ± 0.08	43.58 ± 5.74	0.51 ± 0.01	0.20 ± 0.01
	Methanol	1.91 ± 0.05	5.12 ± 0.08	38.27 ± 1.95	0.17 ± 0.01	0.30 ± 0.01
	Methanol/Water (70%)	1.53 ± 0.11	0.03 ± 0.01	42.19 ± 0.33	0.18 ± 0.01	0.44 ± 0.01
	Water	0.92 ± 0.06	3.74 ± 0.10	24.86 ± 2.24	0.05 ± 0.01	0.30 ± 0.01
*P. kurdica*	Ethyl acetate	1.21 ± 0.02	6.96 ± 0.96	43.06 ± 0.50	0.48 ± 0.01	0.11 ± 0.01
	Methanol	1.81 ± 0.07	1.38 ± 0.10	39.88 ± 0.98	0.20 ± 0.01	0.26 ± 0.01
	Methanol/Water (70%)	1.34 ± 0.09	0.36 ± 0.02	43.51 ± 0.71	0.19 ± 0.01	0.29 ± 0.01
	Water	0.62 ± 0.06	0.90 ± 0.07	12.44 ± 2.17	0.05 ± 0.01	0.21 ± 0.01

* Values are reported as mean ± SD of three parallel measurements. GALAE: galantamine equivalent; KAE: kojic acid equivalent; ACAE: acarbose equivalent; na: not active. Different letters indicate significant differences between the tested extracts (*p* < 0.05).

### 2.5 Molecular Docking

**Figure 4:**
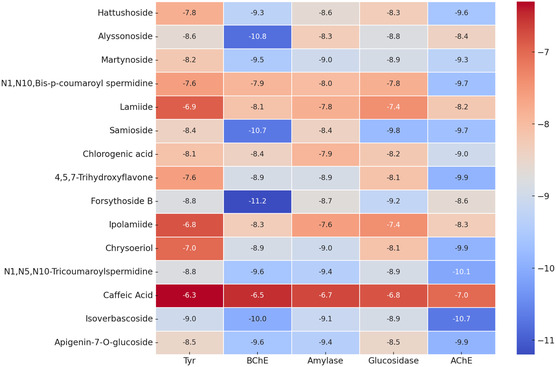
Relevant enzyme result of the docking scores.

**Figure 5:**
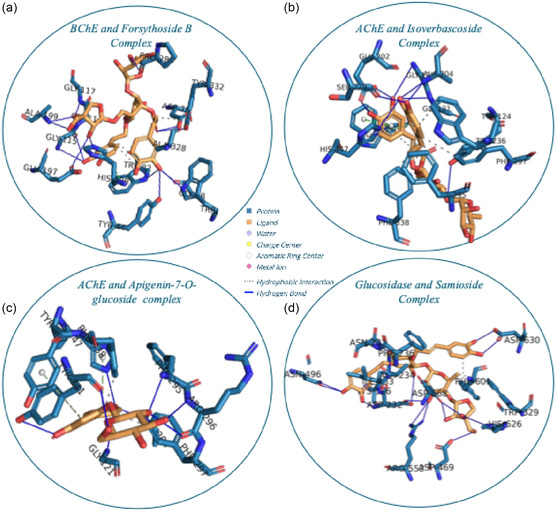
Binding interactions of enzymes with compounds showing the best binding energy; a) Interaction between BChE and Forsythoside B. b) Interaction AChE and Isoverbascoside. c) Interaction between AChE and Apigenin‐7‐O‐glucoside. d) Interaction between Glucosidase and Samioside.

This study employed a comprehensive evaluation approach to assess the potential of bioactive compounds identified in *Phlomis* species for their interactions with standard enzymes. The chemical profiling revealed a diverse array of bioactive compounds, including hattushoside, alyssonoside, martynoside, N1,N10‐bis‐p‐coumaroyl spermidine, lamiide, samioside, chlorogenic acid, 4,5,7‐trihydroxyflavone, forsythoside B, ipolamiide, chrysoeriol, N1,N5,N10‐tricoumaroylspermidine, caffeic acid, isoverbascoside, and apigenin‐7‐O‐glucoside. **Figure** **4** highlights the compounds exhibiting binding energies below −9 kcal mol^−1^, whereas **Table** **7** presents those with binding energies above this threshold. The molecular docking analysis demonstrated binding energies ranging from −11.2 to −6.3 kcal mol^−1^ and root mean square deviation (RMSD) values between 0.2 and 9.2 (**Figure** 4 and **5**).

**Table 7:** The docking score (kcal/mol) and interacting residues of the enzyme.

**Compound**	**Target**	**PDB ID**	**Binding energy**	**RMSD**	**Interaction**		**Binding site**
					**Type**	**Number**	
Isoverbascoside	*Tyr*	5m8o	−9.0	5.29	Hbond	10	HIS A:192; VAL A:211; ASP A:212; GLU A:216; SER A:349; TYR A:369; HIS A:381; GLY A:388; THR A:391; SER A:394
Hattushoside	*BChE*	3djy	−9.3	0.72	Hbond	7	ASP A:70; TRP A:82; ALA A:199; ALA A:199; SER A:287; TYR A:332; HIS A:438
Alyssonoside	*BChE*	3djy	−10.8	9.25	Hbond	14	ASN A:68; TRP A:82; GLY A:116; GLY A:117; THR A:120; THR A:120; TYR A:128; ALA A:199; GLU A:276; ASN A:289; TYR A:332; TRP A:430; HIS A:438; TYR A:440
Martynoside	*BChE*	3djy	−9.5	0.66	Hbond	7	ASP A:70; ASP A:70; THR A:120; GLU A:197; ALA A:199; ALA A:328; HIS A:438
Samioside	*BChE*	3djy	−10.7	9.21	Hbond	15	ASP A:70; ASN A:83; GLY A:115; GLY A:116; GLY A:117; GLN A:119; TYR A:128; GLU A:197; ALA A:199; ALA A:199; ALA A:199; GLU A:276; PRO A:285; ASN A:289; HIS A:438
Forsythoside B	*BChE*	3djy	−11.2	1.73	Hbond	15	ASP A:70; GLY A:78; TRP A:82; GLY A:115; GLY A:115; GLY A:116; GLY A:117; GLU A:197; ALA A:199; ALA A:199; PRO A:285; TYR A:332; TRP A:430; HIS A:438; TYR A:440
N1,N5,N10‐Tricoumaroylspermidine	*BChE*	3djy	−9.6	2.13	Hbond	8	TRP A:82; THR A:120; GLU A:197; ALA A:199; ALA A:199; PRO A:285; TRP A:430; TYR A:440
Isoverbascoside	*BChE*	3djy	−10.0	5.29	Hbond	9	GLY A:115; GLY A:116; GLY A:116; GLY A:117; THR A:120; GLU A:197; ALA A:199; PRO A:285; SER A:287
Apigenin‐7‐O‐glucoside	*BChE*	3djy	−9.6	0.06	Hbond	4	ASN A:68; ASP A:70; THR A:284; SER A:287
Martynoside	*Amylase*	2qv4	−9.0	0.2	Hbond	6	GLN A:63; ASP A:197; ALA A:198; LYS A:200; GLU A:233; GLU A:240
Chrysoeriol	*Amylase*	2qv4	−9.0	0.8	Hbond	5	GLN A:63; ARG A:195; ARG A:195; GLU A:233; HIS A:299
N1,N5,N10‐Tricoumaroylspermidine	*Amylase*	2qv4	−9.4	1.11	Hbond	6	GLN A:63; SER A:108; ARG A:195; HIS A:201; ILE A:235; ASP A:300
Isoverbascoside	*Amylase*	2qv4	−9.1	1.01	Hbond	7	GLN A:63; GLY A:104; ASN A:105; ALA A:106; HIS A:299; ASP A:300; ASP A:300
Apigenin‐7‐O‐glucoside	*Amylase*	2qv4	−9.4	1.08	Hbond	3	HIS A:101; ALA A:198; HIS A:201
Samioside	*Glucosidase*	3w37	−9.8	0.65	Hbond	15	ASP A:70; ASN A:83; GLY A:115; GLY A:116; GLY A:117; GLN A:119; TYR A:128; GLU A:197; ALA A:199; ALA A:199; ALA A:199; GLU A:276; PRO A:285; ASN A:289; HIS A:438
Forsythoside B	*Glucosidase*	3w37	−9.2	0.7	Hbond	11	ALA A:234; ASN A:237; ASP A:357; ASP A:469; SER A:474; PHE A:476; LYS A:506; ASP A:568; GLU A:603; HIS A:626; ASP A:630
Hattushoside	*AChE*	2y2v	−9.6	1.8	Hbond	12	TYR A:72; TYR A:72; THR A:75; LEU A:76; GLY A:121; GLY A:122; TRP A:286; GLN A:291; SER A:293; SER A:293; PHE A:295; TYR A:341
Martynoside	*AChE*	2y2v	−9.3	0.43	Hbond	6	TYR A:72; ASP A:74; TRP A:286; SER A:293; SER A:293; TYR A:341
N1,N10‐Bis p‐coumaroyl spermidine	*AChE*	2y2v	−9.7	4.97	Hbond	4	ASP A:74; ASN A:87; PHE A:295; ARG A:296
Samioside	*AChE*	2y2v	−9.7	0.74	Hbond	9	TYR A:72; TYR A:72; TYR A:72; THR A:75; TYR A:124; SER A:293; PHE A:295; ARG A:296; TYR A:341
Chlorogenic acid	*AChE*	2y2v	−9.0	0.67	Hbond	7	GLY A:121; GLY A:122; TYR A:124; ALA A:204; ALA A:204; ARG A:296; HIS A:447
4,5,7‐Trihydroxyflavone	*AChE*	2y2v	−9.9	0.15	Hbond	3	GLY A:121; GLY A:122; TYR A:341
Chrysoeriol	*AChE*	2y2v	−9.9	0.1	Hbond	3	GLY A:121; GLY A:122; SER A:293
N1,N5,N10‐Tricoumaroylspermidine	*AChE*	2y2v	−10.1	8.51	Hbond	4	TYR A:72; TRP A:286; GLU A:292; PHE A:295
Isoverbascoside	*AChE*	2y2v	−10.7	0.21	Hbond	9	GLY A:120; GLY A:121; TYR A:124; GLU A:202; GLU A:202; ALA A:204; ALA A:204; SER A:229; HIS A:447
Apigenin‐7‐O‐glucoside	*AChE*	2y2v	−9.9	0.25	Hbond	9	GLY A:121; ALA A:204; PHE A:295; ARG A:296; ARG A:296; TYR A:341; TYR A:341; TYR A:341; HIS A:447

In this comprehensive docking investigation, we evaluated the binding profiles of multiple ligands against four enzyme targets (AChE, amylase, BChE, and glucosidase) by employing root‐mean‐square deviation (RMSD ≤ 2 Å), hydrogen bond formation (≥4 h bonds), and binding energy as primary criteria (Table 7). Against the AChE target, hattushoside (RMSD: 1.80 Å, binding energy: −9.60 kcal mol^−1^) engaged with TYR A:72 (twice), THR A:75, LEU A:76, GLY A:121, GLY A:122, TRP A:286, GLN A:291, SER A:293 (twice), PHE A:295, and TYR A:341. Martynoside (RMSD: 0.43 Å, −9.30 kcal mol^−1^) formed contacts with TYR A:72, ASP A:74, TRP A:286, and SER A:293 (twice), as well as TYR A:341. Samioside (RMSD: 0.74 Å, −9.70 kcal mol^−1^) displayed interactions with TYR A:72 (thrice), THR A:75, TYR A:124, SER A:293, PHE A:295, ARG A:296, and TYR A:341 (Figure 5d). Chlorogenic acid (RMSD: 0.67 Å, −9.00 kcal mol^−1^) interacted with GLY A:121, GLY A:122, TYR A:124, ALA A:204 (twice), ARG A:296, and HIS A:447. Isoverbascoside (RMSD: 0.21 Å, −10.70 kcal mol^−1^) bound to GLY A:120, GLY A:121, TYR A:124, GLU A:202 (twice), ALA A:204 (twice), SER A:229, and HIS A:447 (Figure 5b). Finally, Apigenin‐7‐O‐glucoside (RMSD: 0.25 Å, −9.90 kcal mol^−1^) interacted with GLY A:121, ALA A:204, PHE A:295, ARG A:296 (twice), and TYR A:341 (thrice), as well as HIS A:447 (Figure 5c). Common interaction “hotspots” in AChE included TYR A:72, GLY A:121, SER A:293, and TYR A:341. For the Amylase target, martynoside (RMSD: 0.20 Å, −9.00 kcal mol^−1^) showed prominent interactions with GLN A:63, ASP A:197, ALA A:198, LYS A:200, GLU A:233, and GLU A:240, whereas chrysoeriol (RMSD: 0.80 Å, −9.00 kcal mol^−1^) bound GLN A:63, ARG A:195 (twice), GLU A:233, and HIS A:299. N1,N5,N10‐tricoumaroylspermidine (RMSD: 1.10 Å, −9.40 kcal mol^−1^) engaged with GLN A:63, SER A:108, ARG A:195, HIS A:201, ILE A:235, and ASP A:300, while isoverbascoside (RMSD: 1.01 Å, −9.10 kcal mol^−1^) contacted GLN A:63, GLY A:104, ASN A:105, ALA A:106, HIS A:299, and ASP A:300 (twice). Notably, GLN A:63 emerged as a consistent binding site across all ligands targeting amylase. Concerning the BChE target, hattushoside (RMSD: 0.72 Å, −9.30 kcal mol^−1^) established interactions with ASP A:70, TRP A:82, ALA A:199 (twice), SER A:287, TYR A:332, and HIS A:438. Martynoside (RMSD: 0.66 Å, −9.50 kcal mol^−1^) formed contacts with ASP A:70 (twice), THR A:120, GLU A:197, ALA A:199, ALA A:328, and HIS A:438. Forsythoside B (RMSD: 1.73 Å, −11.20 kcal mol^−1^) exhibited binding to ASP A:70, GLY A:78, TRP A:82, GLY A:115 (twice), GLY A:116, GLY A:117, GLU A:197, ALA A:199 (twice), PRO A:285, TYR A:332, TRP A:430, HIS A:438, and TYR A:440 (Figure 5a), whereas apigenin‐7‐O‐glucoside (RMSD: 0.06 Å, −9.60 kcal mol^−1^) interacted with ASN A:68, ASP A:70, THR A:284, and SER A:287 (Figure 5a). Key residues for BChE inhibition included ASP A:70, ALA A:199, and HIS A:438. For the glucosidase target, samioside (RMSD: 0.65 Å, −9.80 kcal mol^−1^) engaged with ASP A:70, ASN A:83, GLY A:115, GLY A:116, GLY A:117, GLN A:119, TYR A:128, GLU A:197, ALA A:199, GLU A:276, PRO A:285, ASN A:289, and HIS A:438 (Figure 5d). In contrast, forsythoside B (RMSD: 0.70 Å, −9.20 kcal mol^−1^) exhibited notable interactions with ALA A:234, ASN A:237, ASP A:357, ASP A:469, SER A:474, PHE A:476, LYS A:506, ASP A:568, GLU A:603, HIS A:626, and ASP A:630. No overlapping residues were observed among the Glucosidase‐binding ligands. Overall, these findings highlight that compounds achieving low RMSD values, robust hydrogen bonding (≥4 h bonds), and favorable binding energies are promising candidates for further optimization. Common residue “hotspots” across specific targets offer valuable insights for rational drug design, underscoring the potential for these ligands to lead the development of novel therapeutic agents.

### 2.6 Binding Free Energy Analysis: MM/PBSA Results and Implications for Ligand Efficacy

**Figure 6:**
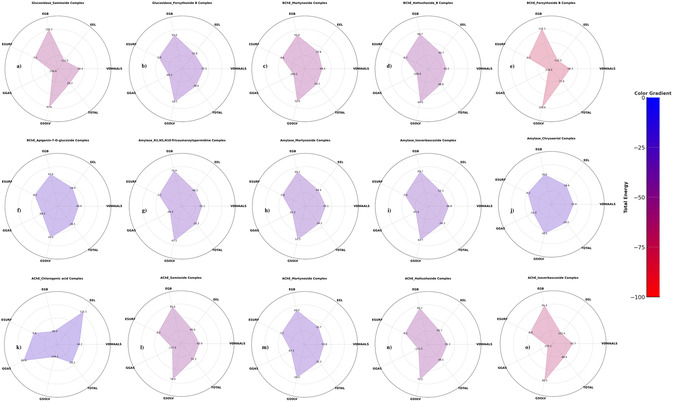
MM/PBSA binding free energy analysis. a) Glucosidase_Samioside complex, b) Glucosidase_Forsythoside_B complex, c) BChE_Martynoside complex, d) BChE_Hattushoside complex, e) BChE_Forsythoside_B complex, f) BChE_Apigenin‐7‐O‐glucoside complex, g) Amylase_N1,N5,N10‐Trioumouranilyspermidine complex, h) Amylase_Martynoside complex, i) Amylase_Isoverbascoside complex, j) Amylase_Chrysoeriol complex, k) AChE_Chlorogenic acid complex, l) AChE_Samioside complex, m) AChE_Martynoside complex, n) AChE_Hattushoside complex, and o) AChE_Isoverbascoside complex.

The present study evaluated the binding stability of a series of protein‐ligand complexes by performing MM/PBSA binding free energy calculations. Key energy components, including van der Waals interactions (VDWAALS), electrostatic energy (EEL), polar solvation energy (EGB), surface tension (ESURF), gas phase energy (GGAS), solvation energy (GSOLV), and total binding free energy (TOTAL), were analyzed to determine their relative contributions to the overall stability (**Figure** **6**). The screening revealed different levels of stability among the complexes. In particular, among the AChE complexes, AChE–isoverbascoside showed the highest stability with a total binding free energy of −68.64 kcal mol^−1^. Significant contributions from electrostatic interactions (EEL = −102.44 kcal mol^−1^) and favorable solvation energy (GSOLV = 86.53 kcal mol^−1^) supported its strong binding profile (Figure 6o). Similarly, AChE–hattushoside showed strong interactions with a total energy of −49.45 kcal mol^−1^, mainly due to significant gas phase energy contributions (GGAS = −120.97 kcal mol^−1^) (Figure 6n). In contrast, AChE–martynoside showed moderate stability with a total energy of −31.22 kcal mol^−1^ and a balanced contribution from van der Waals forces and electrostatics (Figure 6m). Additionally, AChE–chlorogenic acid and AChE–samioside complexes exhibited total binding energies of −34.2 and −51.2 kcal mol^−1^, respectively (Figure 6k,l), further supporting the binding diversity within the AChE complexes. The binding profiles of the amylase complexes were also diverse. Amylase–isoverbascoside emerged as a stable candidate with a total binding energy of −34.32 kcal mol^−1^, while Amylase–martynoside showed a total energy of −34.21 kcal mol^−1^, and Amylase‐N1,N5,N10‐trioumouranilyspermidine had a total energy of −32.2 kcal mol^−1^ (Figure 6i,h,g). These complexes show consistent gas phase and solvation energy contributions. In comparison, Amylase–chrysoeriol showed lower binding stability with a total energy of −24.54 kcal mol^−1^, indicating weaker interactions in this complex (Figure 6j). Among the panel of BChE complexes, BChE‐forsythoside B showed excellent stability with the most significant binding energy reported at −77.86 kcal mol^−1^. The stability of this complex was mainly determined by highly favorable gas phase energy contributions (GGAS = −178.46 kcal mol^−1^) together with compensatory solvation energy (GSOLV = 100.60 kcal mol^−1^) (Figure 6e). In addition, both BChE–hattushoside (−54.2 kcal mol^−1^; Figure 6d) and BChE–martynoside (−32.2 kcal mol^−1^; Figure 6c) complexes exhibited remarkable binding affinity toward the target enzyme. The glucosidase complexes showed promising features, with glucosidase‐samioside standing out due to its sizeable total energy of −59.25 kcal mol^−1^. This complex showed significant electrostatic contributions (EEL = −122.45 kcal mol^−1^) and a favorable solvation energy (GSOLV = 97.60 kcal mol^−1^) (Figure 6a). Similarly, glucosidase‐forsythoside B showed a moderate binding profile with a total energy of −34.56 kcal mol^−1^ (Figure 6b).

Based on a detailed analysis of the 10 nanosecond MD simulations, AChE‐isoverbascoside, AChE‐samioside, BChE‐forsythoside B, and BChE‐hattushoside were selected for further investigation. The above selection was based on their strong binding profiles, high stability, and favorable energetic properties, making them ideal candidates for extended simulations and experimental verification. These findings underscore the strong interplay between different energy components in modulating binding stability. Robust van der Waals interactions and energies in the gas phase were generally reported to favor stability. In contrast, electrostatic interactions and solvation energies often played an important role in modulating the binding free energy. The identified complexes, particularly isoverbascoside, hattushoside, samioside, martynoside, and forsythoside B, show good energy profiles, making them candidates for further MD simulations and drug design studies. This in‐depth study provides substantial insight into the binding efficiencies of these molecules, thus providing a robust platform for developing therapeutic agents targeting proteins associated with disease.

### 2.7 Stability and Flexibility in MD Simulation

The present study aims to identify potential therapeutic drugs through a comprehensive investigation of the molecular interactions between specific ligands and target proteins, with a particular focus on elucidating their binding sites. The selection of complexes for evaluation was based on the results of the AChE‐isoverbascoside, AChE‐samioside, BChE‐forsythoside B, and BChE‐hattushoside as complexes. These complexes exhibited robust selectivity and stability in their interactions, as well as important criteria such as the presence of hydrogen‐bonding residues and the results of MM/PBSA binding free energy calculations. Subsequently, the complexes underwent MD simulations, which afforded more significant insight into their biological efficacy and protein binding capacities, thus facilitating a more comprehensive evaluation of their potential as therapeutic agents.

**Figure 7:**
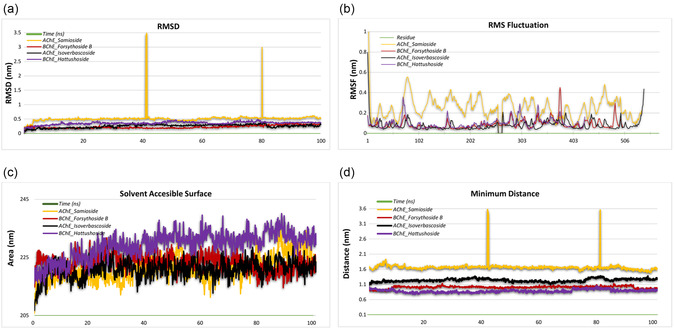
Presentation of MD simulations in graphical form; a) RMSD of AChE_Isoverbascoside, AChE_Samioside, BChE_Forsythoside B, and BChE_Hattushoside complexes. b) RMSF of AChE_Isoverbascoside, AChE_Samioside, BChE_Forsythoside B, and BChE_Hattushoside complexes. c) Solvent accessibility of AChE_Isoverbascoside, AChE_Samioside, BChE_Forsythoside B, and BChE_Hattushoside complexes. d) Minimum distance of AChE_Isoverbascoside, AChE_Samioside, BChE_Forsythoside B, and BChE_Hattushoside complexes.

The structural stability of the four ligand–protein complexes was rigorously evaluated based on their RMSD profiles over a 100‐nanosecond MD simulation. The AChE‐isoverbascoside complex showed significant conformational variability, with RMSD values rising steadily from 0.2 nm to ≈0.6 nm during the first 40 ns. This increase suggests a progressive structural adjustment, possibly due to shifts in the binding region or repositioning of the ligand. Beyond 40 ns, the RMSD values fluctuated between 0.4 and 0.6 nm, indicating a dynamic equilibrium without further significant conformational stabilization. Specific inflection points, such as a rapid increase around 20 ns, highlight transient destabilizations associated with localized structural rearrangements. The AChE‐samioside complex, in contrast, maintained a stable RMSD profile throughout the simulation, consistently between 0.3 and 0.4 nm. This stability, characterized by negligible variability, indicates a tightly maintained binding conformation with minimal perturbations. No sharp inflection points or abrupt changes were observed, reflecting the ligand–protein interaction's robustness and the binding region's structural integrity. The BChE‐forsythoside B complex showed a more gradual increase in RMSD during the first 60 ns, starting from 0.2 nm and stabilizing around 0.4–0.5 nm. This trend suggests a slow structural adaptation, with notable inflection points at 25 and 50 ns that may represent conformational shifts leading to eventual stabilization. The plateau in RMSD values at 60 ns reflects the achievement of a semi‐stable configuration. However, the earlier fluctuations suggest moderate flexibility in the binding interactions (**Figure** **7**a). Root mean square fluctuation (RMSF) analysis provides valuable insights into the conformational dynamics and adaptability of residues within AChE and BChE complexes. These observations highlight how ligand binding affects residue flexibility and reveal structural features critical for protein function and ligand specificity. The AChE complexes with the ligands isoverbascoside and samioside exhibit a mean flexibility of 0.165, indicating a predominantly stable structural configuration. Regions of low flexibility, such as residues 9–10, 16–18, and 257–264, suggest a robust structural framework critical for maintaining the integrity of the binding site. The extended rigidity across residues 402–411 and 526–529 further emphasizes structural stabilization within functional domains, potentially minimizing conformational entropy penalties during ligand binding. Conversely, regions of high flexibility, including residues 75–86 and 437–440, correspond to regions that may facilitate allosteric regulation or adapt to environmental or ligand‐induced perturbations. In particular, residues 463–468 and 540–543 show localized fluctuations, suggesting a dynamic role in substrate recognition or transient interactions with neighboring structural elements. The flexibility patterns suggest that AChE complexes balance local adaptability and overall structural rigidity. This duality is essential for maintaining enzymatic efficiency while allowing precise ligand‐induced conformational shifts. The BChE complexes, particularly those bound to forsythoside B and hattushoside, have a lower mean flexibility of 0.092, indicating a more tightly regulated conformational state. Regions of low flexibility, such as residues 532–543, form a rigid backbone that likely supports the structural integrity of the active site. This stability is critical for the hydrolytic function of BChE as it minimizes unnecessary conformational noise. In contrast, regions of high flexibility, including residues 70–77 and 280–284, highlight segments that are likely peripheral or involved in ligand‐induced dynamics. The increased fluctuation in residues 358–363 and 425–426 may facilitate transient conformations required for substrate accessibility or release of hydrolysis products. Of note is the increased flexibility observed in residues 485–490, which may correspond to loops or noncatalytic regions that contribute to the substrate versatility of BChE. Comparison of AChE and BChE complexes reveals distinct dynamic profiles reflecting their functional specialization. AChE exhibits a broader distribution of both high and low‐flexibility regions, supporting its role in fine‐tuned neurotransmission processes. In contrast, the narrower distribution of BChE flexibility values underscores its broader substrate range and nonspecific hydrolytic activity. The flexibility observed in residues distal to the active site, such as 437–440 in AChE and 358–363 in BChE, suggests potential allosteric regulation. These regions could act as conformational relays, transmitting structural changes from distal binding events to the catalytic core. In addition, regions with increased flexibility in ligand‐specific contexts, such as 463–468 in AChE‐isoverbascoside, may provide insights into how different ligands modulate enzyme activity. RMSF results highlight the delicate interplay between rigidity and flexibility in protein‐ligand complexes. Residues with low RMSF values often correlate with structurally conserved domains critical for ligand binding and enzymatic activity, while high RMSF regions indicate adaptable or allosterically modulated sites. From a drug design perspective, targeting regions of high flexibility could enable the development of allosteric inhibitors or stabilizers. In addition, the differential flexibility patterns observed across complexes could guide mutagenesis experiments aimed at modulating enzymatic specificity or stability. In conclusion, the structural dynamics elucidated by RMSF analysis provide a granular view of residue‐specific contributions to protein function. The contrasting flexibility profiles between AChE and BChE complexes underscore their functional divergence, while common patterns of ligand‐induced flexibility highlight conserved mechanisms of molecular adaptability (Figure 7b). The solvent exposure of the ligand–protein complexes was evaluated using the SASA method, which provides insights into the complexes' structural compactness and solvent interaction. The analysis revealed significant variations across the studied systems. For instance, the AChE‐samioside complex exhibited the highest SASA values, averaging ≈221 nm^2^, indicative of an extensive surface area and greater solvent interaction. This suggests a less compact structure, allowing more dynamic interactions with the surrounding solvent. In contrast, the BChE‐forsythoside B complex exhibited moderate SASA values, ranging from 214 to 223 nm^2^, suggesting a balance between solvent exposure and structural compactness. Among the complexes, the BChE‐hattushoside exhibited the lowest SASA values, with an average of ≈229 nm^2^, indicating a relatively compact structure with limited solvent interaction. The structural stability of these complexes was further investigated by analyzing the minimum binding distance in the binding region. The results demonstrated varying degrees of stability and flexibility. For example, the AChE‐samioside complex showed a progressive increase in minimum distance values, ranging from 0.8 to 1.6 nm over the simulation period. This suggests a flexible binding interaction and reduced stability, which could be attributed to the dynamic nature of its binding conformation. Similarly, the AChE‐isoverbascoside complex demonstrated increasing fluctuations in the 0.7 to 1.3 nm range, signifying moderate flexibility. In contrast, the BChE‐hattushoside and BChE‐forsythoside B complexes demonstrated consistent stability, maintaining minimum distances within the 0.4–0.6 nm range throughout the simulation. This observation suggests the presence of a compact and stable binding region, which is indicative of strong interactions and reduced flexibility. This enhanced stability could improve the functional efficacy of these complexes by preserving robust binding conformations over time. The findings underscore the intricate relationship between solvent exposure, surface area, and structural dynamics in determining the stability of ligand–protein interactions. The elevated SASA values observed for complexes such as AChE‐samioside imply extensive solvent interaction and structural flexibility. In contrast, the diminished SASA and stable minimum distance values in complexes like BChE‐Hattushoside underscore their compact and stable nature. These findings offer a foundational understanding of the dynamics of ligand–protein interactions, which could have implications for drug design and biomolecular engineering (Figure 7c). The minimum distance between atoms in the binding region is a critical indicator of ligand–protein complexes' structural stability and dynamics. The complex behaviors exhibited by each complex analyzed in this study indicate their unique stability and dynamic alterations throughout the simulation period. The AChE‐isoverbascoside complex demonstrates moderate fluctuations in minimum distance values, ranging from 1.08 to 1.30 nm. A slight decrease in trend is observed over time, with the minimum distance dropping from 1.20 nm at 20 ns to 1.18 nm at 80 ns. These variations suggest a stable binding region with minor dynamic adjustments. The binding conformation remains compact, maintaining structural integrity throughout the simulation. The AChE‐samioside complex shows relatively higher fluctuations, with minimum distances between 1.48 and 1.88 nm. A slight decrease in distance is observed for the simulation, from an average of 1.66 nanometers (nm) at 20 nanoseconds (ns) to 1.60 nm at 80 ns. This behavior reflects moderate flexibility in the binding region, with the binding conformation slightly tightening over time. The BChE‐forsythoside B complex displays a stable profile, with minimum distances ranging from 0.86 to 1.12 nm. Minimal fluctuations are observed, as the average distance remains consistent from 1.00 nm at 20 ns to 0.98 nm at 80 ns, indicating a tightly packed binding region with negligible dynamic alterations, thereby highlighting strong ligand–protein interactions. The BChE‐hattushoside complex demonstrates a compact and stable binding conformation, with minimum distances consistently ranging between 0.77 and 0.95 nm. The simulation reveals minimal fluctuations, with a slight decrease in distance from 0.89 nm at 20 ns to 0.87 nm at 80 ns. This observation suggests a tightly bound and structurally stable interaction, exhibiting negligible flexibility in the binding region. Among the complexes under consideration, the BChE‐hattushoside complex demonstrates the most stable and compact binding conformation, exhibiting minimal fluctuations throughout the simulation. Conversely, the AChE‐samioside complex exhibits slightly greater variability, suggesting moderate flexibility in its binding region. The other complexes, specifically AChE‐isoverbascoside and BChE‐forsythoside B, maintain stability with minor fluctuations, indicative of compact and reliable interactions. The findings indicate that ligand–protein complexes exhibit varying degrees of stability and flexibility. The BChE‐hattushoside complex, characterized by its highly compact conformation, is the most stable complex. In contrast, AChE‐samioside demonstrates moderate dynamic behavior, suggesting a relatively less compact binding conformation (Figure 7d).

**Figure 8:**
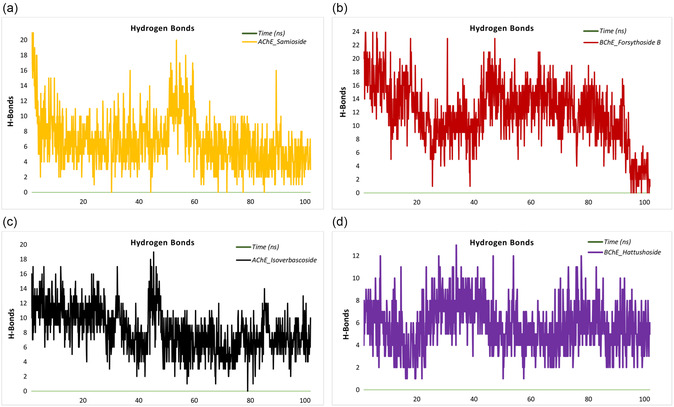
Hydrogen bond analysis of ligand–protein complexes over a 100–ns simulation period. a) Hydrogen bonds in the AChE_Isoverbascoside complex. b) Hydrogen bonds in the AChE_Samioside complex. c) Hydrogen bonds in the BChE_Forsythoside B complex. d) Hydrogen bonds in the BChE_Hattushoside complex.

During the 100–ns simulation of the AChE‐isoverbascoside complex, the number of hydrogen bonds exhibited fluctuations between a minimum of 0 and a maximum of 19. During the initial 20 ns, the hydrogen bond count demonstrated significant variability, ranging from 5 to 17, with a mean value of ≈10. As the simulation progressed (20–60 ns), a more stable pattern emerged, with the bond count varying between 1 and 19 and averaging around 9. In the final stages of the simulation (60–100 ns), the hydrogen bond count stabilized, typically fluctuating between 0 and 14, with a mean value of 7. This trend suggests a decreasing number of hydrogen bonds over time, indicating that the complex may not achieve full stability (**Figure** **8**c). For the AChE‐samioside complex, the number of hydrogen bonds fluctuated between 0 and 24 throughout the simulation. During the initial 20 ns, the bond count ranged from 2 to 24, with a mean of ≈8, suggesting an adaptation period to the binding site. From 20 to 60 ns, the bond count demonstrated greater stability, generally remaining between 0 and 20 and averaging around 8. However, during the final 60–100 ns phase, the bond count exhibited a decline in stability, fluctuating between 0 and 16 with a mean value of 5, suggesting inconsistent and unstable binding interactions (Figure 8a). In the case of the BChE‐forsythoside B complex, the hydrogen bond count fluctuated between 0 and 25. During the initial 20 ns, the count ranged from 5 to 25, with a mean value of ≈15, reflecting an initial stabilization phase. Between 20 and 60 ns, the bond count demonstrated enhanced stability, fluctuating between 1 and 23 with an average of 11. In the final 60–100 ns interval, the bond count maintained relative stability, fluctuating between 0 and 21 and averaging 10, indicating moderate stability despite a slight decrease in the number of bonds over time (Figure 8b). The BChE‐hattushoside complex exhibited hydrogen bond counts ranging from 1 to 13 throughout the simulation. During the initial 20 ns, the bond count fluctuated between 1 and 12, with a mean value of 5, indicating an early stabilization phase. From 20 to 60 ns, the bond count ranged from 1 to 13, with an average of 6 reflecting greater stability. In the final 60–100 ns phase, the count ranged between 2 and 12, with a mean value of 5, suggesting steady binding interactions but no significant improvement in stability over time (Figure 8d). These observations provide insights into the dynamic nature of hydrogen bonding in these complexes and highlight the varying degrees of stability achieved during the simulation, with particular emphasis on instances where binding stability decreases or remains inconsistent.

## 3 Conclusion

The present study elucidated the phytoconstituents, antioxidant, and enzyme inhibitory activities of extracts obtained from *P. herba‐venti* and *P. kurdica* aerial parts. MeOH, or 70% MeOH, recovered the highest TPC among the three species. The same solvents also extracted the highest TFC for *P. herba‐venti* and *P. kurdica,* while for *P. fruticosa* it was the EtOAc. Phenolic acids, phenylethanoids, flavonoids, iridoids, organic acids, terpenes, and fatty acids were identified. The three species and their extracts had variable effects on biological activities. 70% MeOH extract of *P. herba‐venti* exerted the best radical scavenging and ions‐reducing properties. EtOAc extract from *P. fruticosa* displayed the best chelating power, while its three other extracts showed the highest total antioxidant activity. In contrast, the highest anti‐BChE and anti‐AChE activities were obtained respectively from the EtOAc extract from *P. kurdica* and MeOH extract of *P. herba‐venti.* The three organic extracts of the species displayed significant and comparable anti‐Tyr effects. Furthermore, molecular docking and MD simulation studies underscored the therapeutic potential of bioactive compounds, including isoverbascoside, samioside, forsythoside B, and hattushoside. These ligands exhibited robust binding profiles, considerable stability, and conducive energetic properties when engaged with enzyme targets such as AChE, BChE, amylase, and glucosidase. In‐depth MM/PBSA binding free energy analysis and hydrogen bond dynamics provided valuable insights into ligand–protein complexes' structural stability, flexibility, and solvent interactions, underscoring their suitability as lead compounds for further drug design efforts. In conclusion, this study appraised that these three *Phlomis* species are a rich source of biologically active compounds with potential for future development of phytopharmaceuticals targeting specific oxidative stress‐linked diseases. The selection of appropriate extraction solvents is crucial for the targeted biological activity. The isolation of bioactive molecules, the illustration of their mechanism of action and safety, and in vivo studies are recommended.

## 4 Experimental Section

4.1

4.1.1

##### Plant Collection

In 2023, botanical specimens were collected from the East Region of Turkey. The details can be found below. Dr. Uğur Çakilcioğlu conducted the taxonomic identification, and a voucher specimen was preserved in the herbarium of Munzur University. The aerial parts were separated, dried in the shade at ambient temperature, pulverized, and stored away from light.

*Phlomis fruticosa* L.: Between Şahinkaya and Nurali village, Elazığ: Voucher Number: UC‐22‐20.

*Phlomis* *herba‐venti* L.: North of Şahinkaya village 2 km. Elazığ: Voucher Number: UC‐22‐15.

*Phlomis kurdica* Rech.f. : Çaydaçıra Location. Elazığ: Voucher Number: UC‐23‐21.

##### Plant Extract Preparation

The extraction procedure included four solvents: ethyl acetate, methanol, a 70% methanol/water mixture, and water. Each 10 g sample was macerated with 200 mL of ethyl acetate, methanol, and a mixture of methanol and water for 24 h at ambient temperature. The aqueous extract was prepared by infusing 10 g of plant material in boiling water for 15 min. Organic solvents were removed via evaporation under low pressure, and the aqueous extract was subjected to freeze‐drying.

##### Assay for Total Phenolic and Flavonoid Contents

Total phenolics and flavonoids were quantified according to the procedures outlined by.^[^ ^40^ ^]^ gallic acid (GA) and rutin (R) were used as reference standards in the studies, with results expressed as gallic acid equivalents (GAE) and rutin equivalents (RE). The experimental details are given in the supplemental materials.

##### LC‐MS/MS Metabolomic Analysis

Liquid chromatography and mass spectrometry (UHPLC/MS/MS) were used to analyze various extracts. The Dionex Ultimate 3000RS UHPLC system was used for this analysis, which was fitted with a mass spectrometer (Q‐Exactive Orbitrap, Thermo, USA). Our previous publication included all analytical details,^[^ ^41^ ^]^ and all details are also presented in the supplemental materials. Trace Finder 3.1 (Thermo Scientific) software was analyzed the raw files. The secondary metabolites were identified based on our previous published works and our/online databases (Metlin, Massbank of North America, m/z Cloud). The exact molecular mass, isotopic pattern, characteristic fragment ions, and retention time were used to identify the secondary metabolites.

##### Assays for In Vitro Antioxidant Capacity

In accordance with the methodologies detailed in our prior publication,^[^ ^42^ ^]^ various antioxidant tests were carried out. The outcomes were represented as milligrams of Trolox equivalents (TE) per gram for the DPPH, ABTS radical scavenging, CUPRAC, and FRAP tests. In millimoles of TE per gram of extract, the phosphomolybdenum (PBD) test examined antioxidant potential, and in milligrams of disodium edetate equivalents (EDTAE) per gram of extract, the metal chelating activity (MCA) was determined. The experimental details are given in the supplemental materials.

##### Inhibitory Effects Against Some Key Enzymes

In accordance with the established protocols,^[^ ^42^ ^]^ experiments on enzyme inhibition were performed on the samples. Acarbose equivalents (ACAE) per gram of extract were used to measure the activities that inhibit amylase and glucosidase, while milligrams of galanthamine equivalents (GALAE) per gram of extract were used to examine the inhibition of acetylcholinesterase (AChE) and butyrylcholinesterase (BChE). The amount of tyrosinase inhibition for each gram of extract was measured in milligrams of kojic acid equivalents (KAE). The experimental details are given in the supplemental materials.

##### Molecular Docking

In the present work, a set of enzymes such as AChE, BChE, Tyr, amylase, and glucosidase were retrieved from the Protein Data Bank‐PDB: https://www.rcsb.org/. Re‐docking studies were performed in these enzymes with their co‐crystallized ligands to validate our docking protocol by calculating RMSD values.^[^ 43, 44, 45 ^]^ Enzyme structures were prepared for docking by removing co‐crystallized ligands, cofactors, and water molecules using BIOVIA Discovery Studio Visualizer (v4.5).^[^ 46, 47 ^]^ The ligands were downloaded from the PubChem database (https://pubchem.ncbi.nlm.nih.gov/) and optimized for geometry and energy using Avogadro (v1.2.0). The MGL Tools (v1.5.6) were then used to complete the enzyme structures.^[^ 45, 48 ^]^ The following grid box dimensions and center coordinates were utilized for docking simulations: The first grid box (AChE, PDB ID: 2y2v) had dimensions of 22 x 30 x 40 Å and coordinates at 31.062, 20.311, 11.947. The second grid box (BChE, PDB ID: 3djy) had dimensions of 30 Å × 30 Å × 30 Å and coordinates at 44.794, −19.63, −25.227. The third grid box (Tyr, PDB ID: 5m8o) had dimensions of 26 Å × 26 Å × 28 Å and coordinates at −13. The amylase (PDB ID: 2qv4) grid size was set at 28 Å × 28 Å × 24 Å, and its coordinates were set at 14.188, 48.964, 22.886. The glucosidase (PDB ID: 3w37) grid size was set at 42 Å × 52 Å × 54 Å, and its coordinates were set at 3.091, −8.008, −4.08. A comprehensive review of the available data concerning the inhibitor binding for the aforementioned enzymes was conducted, and the grid dimensions were set as previously defined to facilitate docking simulations. The active binding sites were defined based on the literature's available data on inhibitor binding.^[^ 43, 49, 50, 51, 52 ^]^ Molecular docking simulations were performed with AutoDock Vina (v1.1.2) using grid boxes designed according to the method proposed by Trott and Olson.^[^ ^53^ ^]^ To better understand the enzyme‐ligand interactions, we utilized the Protein‐Ligand Interaction Profiler (PLIP) (https://plip‐tool.biotec.tu‐dresden.de/plip‐web/plip/index), which pointed out critical interactions, mainly hydrogen bonds.^[^ 46, 47 ^]^ These analysis methods confirmed that our docking results were precise and reliable.

##### Calculation of MM/PBSA Free Energy to Determine Ligand‐Binding Affinity

The study employed the gmx_MMPBSA tool (https://valdes‐tresanco‐ms.github.io/gmx_MMPBSA/dev/getting‐started/) to evaluate the stability and calculate the binding free energy of various molecular complexes. Ten‐nanosecond MD simulations were conducted to evaluate the stability of the aforementioned complexes. The complexes BChE‐hattushoside, BChE‐martynoside, BChE‐forsythoside B, BChE‐apigenin‐7‐O‐glucoside, Amylase‐martynoside, Amylase‐chrysoeriol, Amylase_N1,N5,N10‐tricoumaroylspermidine, Amylase‐isoverbascoside, Glucosidase‐samioside, Glucosidase‐forsythoside B, AChE‐hattushoside, AChE‐martynoside, AChE‐samioside, AChE‐chlorogenicacid, AChE‐isoverbascoside, and AChE‐apigenin‐7‐O‐glucoside were subjected to MD simulations. Based on these simulations, the complexes demonstrating the highest stability were identified and selected for further investigation. These selected complexes were then subjected to extended 10‐ns MD simulations to gain deeper insights into their interactions and potential as therapeutic candidates.^[^ 54, 55 ^]^

##### MD Simulation

The simulations were executed in accordance with the CHARMM‐gui platform (https://charmm‐gui.org) MD. The configuration of the system was constructed utilizing the Solution Builder tool, which was previously reported by Jo et al.^[^ ^56^ ^]^ The parametrization of the protein was performed using the CHARMM36m force field, as described by Yagi et al.^[^ ^57^ ^]^ and Maier et al.^[^ ^58^ ^]^ It is imperative to ensure the maintenance of a minimum distance of 10 Å between the boundaries of the box and the protein. The TIP3P water molecules periodic boundary box was formed around the protein. To obtain the required 0.15 m NaCl concentration and to balance the charge of the system, counterions were introduced. To adjust the electrostatic and van Der Waals interactions, the formal cut‐off method due to Verlet was employed, while the LINCS algorithm was implemented to constrain bond lengths. The long‐range electrostatics were calculated with the PME method. Subsequent to this, energy minimization was performed using the steepest descent method until the variation in potential energy fell below 1000 kJ mol nm^−1^. At this juncture, the system was prepared for equilibration, which was carried out under NVT and NPT settings set at 310 K to ensure thermodynamic stability. Production simulations were subsequently carried out for a total of 100 ns (nstep = 50 000 000) using GROMACS 2023.2. These simulations were conducted for the AChE‐isoverbascoside, AChE‐samioside, BChE‐forsythoside B, and BChE‐hattushoside complexes to investigate their stability, interactions, and potential as therapeutic candidates.

##### Statistical Analysis

Experiments were performed in triplicate, and differences between the extracts were compared using One‐way ANOVA (by Tukey's assay), and GraphPad Prism (version 9.2) was used for the analysis. The *p* value of less than 0.05 was deemed to be statistically significant.

## Conflict of Interest

The authors declare no conflict of interest.

## Supporting information

Supplementary Material

## Data Availability

The data that support the findings of this study are available from the corresponding author upon reasonable request.
