# Supplementary material for: Chemical Characterization, Antioxidant and Enzyme‐Inhibitory Activities of Different Extracts from Three Phlomis Species
Source: ChemistryOpen. 2025 May 12;14(8):e202500004. doi: 10.1002/open.202500004 (PMC12957883; doi:10.1002/open.202500004)
Supplement: Supplementary file 1 — Supplementary Material [file OPEN-14-e202500004-s001.pdf]

Assays for Total Phenolic and Flavonoid Contents

The total phenolic content was determined by employing the methods given in the literature with some modification. Sample solution (0.25 mL) was mixed with diluted Folin–Ciocalteu reagent (1 mL, 1:9, v/v) and shaken vigorously. After 3 min, Na_2CO_3 solution (0.75 mL, 1%) was added and the sample absorbance was read at 760 nm after a 2 h incubation at room temperature. The total phenolic content was expressed as milligrams of gallic acid equivalents (mg GAE/g extract)[1].

The total flavonoid content was determined using the $AlCl_3$ method. Briefly, sample solution (1 mL) was mixed with the same volume of aluminum trichloride (2%) in methanol. Similarly, a blank was prepared by adding sample solution (1 mL) to methanol (1 mL) without $AlCl_3$. The sample and blank absorbances were read at 415 nm after a 10 min incubation at room temperature. The absorbance of the blank was subtracted from that of the sample. Rutin was used as a reference standard and the total flavonoid content was expressed as milligrams of rutin equivalents (mg RE/g extract) [1].

Determination of Antioxidant and Enzyme Inhibitory Effects

Antioxidant (DPPH and ABTS radical scavenging, reducing power (CUPRAC and FRAP), phosphomolybdenum and metal chelating (ferrozine method)) and enzyme inhibitory activities (cholinesterase (Elmann’s method), tyrosinase (dopachrome method), α-amylase (iodine/potassium iodide method), α -glucosidase (chromogenic PNPG method) and pancreatic lipase (*p*-nitrophenyl butyrate (p-NPB) method) were determined using the methods previously described by Uysal et al. [1] and Grochowski et al. [2]

For the DPPH (1,1-diphenyl-2-picrylhydrazyl) radical scavenging assay: Sample solution was added to 4 mL of a 0.004% methanol solution of DPPH. The sample absorbance was read at 517 nm after a 30 min incubation at room temperature in the dark. DPPH radical scavenging activity was expressed as milligrams of trolox equivalents (mg TE/g extract).

For ABTS (2,2′-azino-bis(3-ethylbenzothiazoline) 6-sulfonic acid) radical scavenging assay: Briefly, ABTS+ was produced directly by reacting 7 mM ABTS solution with 2.45 mM potassium persulfate and allowing the mixture to stand for 12–16 in the dark at room temperature. Prior to beginning the assay, ABTS solution was diluted with methanol to an absorbance of 0.700 ± 0.02 at 734 nm. Sample solution was added to ABTS solution (2 mL) and mixed. The sample absorbance was read at 734 nm after a 30 min incubation at room temperature. The ABTS radical scavenging activity was expressed as milligrams of trolox equivalents (mg TE/g extract).

For CUPRAC (cupric ion reducing activity) activity assay: Sample solution was added to premixed reaction mixture containing $CuCl_2$ (1 mL, 10 mM), neocuproine (1 mL, 7.5 mM) and NH_4Ac buffer (1 mL, 1 M, pH 7.0). Similarly, a blank was prepared by adding sample solution (0.5 mL) to premixed reaction mixture (3 mL) without $CuCl_2$. Then, the sample and blank absorbances were read at 450 nm after a 30 min incubation at room temperature. The absorbance of the blank was subtracted from that of the sample. CUPRAC activity was expressed as milligrams of trolox equivalents (mg TE/g extract).

For FRAP (ferric reducing antioxidant power) activity assay: Sample solution was added to premixed FRAP reagent (2 mL) containing acetate buffer (0.3 M, pH 3.6), 2,4,6-tris(2-pyridyl)-S-triazine (TPTZ) (10 mM) in 40 mM HCl and ferric chloride (20 mM) in a ratio of 10:1:1 (v/v/v). Then, the sample absorbance was read at 593 nm after a 30 min incubation at room temperature. FRAP activity was expressed as milligrams of trolox equivalents (mg TE/g extract).

For phosphomolybdenum method: Sample solution was combined with 3 mL of reagent solution (0.6 M sulfuric acid, 28 mM sodium phosphate and 4 mM ammonium molybdate). The sample absorbance was read at 695 nm after a 90 min incubation at 95 °C. The total antioxidant capacity was expressed as millimoles of trolox equivalents (mmol TE/g extract).

For metal chelating activity assay: Briefly, sample solution was added to $FeCl_2$ solution (0.05 mL, 2 mM). The reaction was initiated by the addition of 5 mM ferrozine (0.2 mL). Similarly, a blank was prepared by adding sample solution (2 mL) to $FeCl_2$ solution (0.05 mL, 2 mM) and water (0.2 mL) without ferrozine. Then, the sample and blank absorbances were read at 562 nm after 10 min incubation at room temperature. The absorbance of the blank was sub-tracted from that of the sample. The metal chelating activity was expressed as milligrams of EDTA (disodium edetate) equivalents (mg EDTAE/g extract).

For Cholinesterase (ChE) inhibitory activity assay: Sample solution (was mixed with DTNB (5,5-dithio-bis(2-nitrobenzoic) acid, Sigma, St. Louis, MO, USA) (125 µL) and AChE (acetylcholines-terase (Electric ell acetylcholinesterase, Type-VI-S, EC 3.1.1.7,Sigma)), or BChE (butyrylcholinesterase (horse serum butyrylcholinesterase, EC 3.1.1.8, Sigma)) solution (25 µL) in Tris–HCl buffer (pH 8.0) in a 96-well microplate and incubated for 15 min at 25 °C. The reaction was then initiated with the addition of acetylthiocholine iodide (ATCI, Sigma) or butyrylthiocholine chloride (BTCl, Sigma) (25 µL). Similarly, a blank was prepared by adding sample solution to all reaction reagents without enzyme (AChE or BChE) solution. The sample and blank absorbances were read at 405 nm after 10 min incubation at 25 °C. The absorbance of the blank was subtracted from that of the sample and the cholinesterase inhibitory activity was expressed as galanthamine equivalents (mgGALAE/g extract).

For Tyrosinase inhibitory activity assay: Sample solution was mixed with tyrosinase solution (40 µL, Sigma) and phosphate buffer (100 µL, pH 6.8) in a 96-well microplate and incubated for 15 min at 25 °C. The reaction was then initiated with the addition of L-DOPA (40 µL, Sigma). Similarly, a blank

was prepared by adding sample solution to all reaction reagents without enzyme (tyrosinase) solution. The sample and blank absorbances were read at 492 nm after a 10 min incubation at 25 °C. The absorbance of the blank was subtracted from that of the sample and the tyrosinase inhibitory activity was expressed as kojic acid equivalents (mgKAE/g extract).

For α-amylase inhibitory activity assay: Sample solution was mixed with α-amylase solution (ex-porcine pancreas, EC 3.2.1.1, Sigma) (50 µL) in phosphate buffer (pH 6.9 with 6 mM sodium chloride) in a 96-well microplate and incubated for 10 min at 37 °C. After pre-incubation, the reaction was initiated with the addition of starch solution (50 µL, 0.05%). Similarly, a blank was prepared by adding sample solution to all reaction reagents without enzyme (α-amylase) solution. The reaction mixture was incubated 10 min at 37 °C. The reaction was then stopped with the addition of HCl (25 µL, 1 M). This was followed by addition of the iodine-potassium iodide solution (100 µL). The sample and blank absorbances were read at 630 nm. The absorbance of the blank was subtracted from that of the sample and the α-amylase inhibitory activity was expressed as acarbose equivalents (mmol ACE/g extract).

For α-glucosidase inhibitory activity assay: Sample solution was mixed with glutathione (50 µL), α-glucosidase solution (from Saccharomyces cerevisiae, EC 3.2.1.20, Sigma) (50 µL) in phosphate buffer (pH 6.8) and PNPG (4-N-trophenyl-α-D-glucopyranoside, Sigma) (50 µL) in a 96-well microplate and incubated for 15 min at 37 °C. Similarly, a blank was prepared by adding sample solution to all reaction reagents without enzyme (α-glucosidase) solution. The reaction was then stopped with the addition of sodium carbonate (50 µL, 0.2 M). The sample and blank absorbances were read at 400 nm. The absorbance of the blank was subtracted from that of the sample and the α-glucosidase inhibitory activity was expressed as acarbose equivalents (mmol ACE/g extract).

UHPLC-MS/MS analysis

Analysis of different extracts was carried out on liquid chromatography coupled with mass spectrometry (UHPLC/MS/MS) using a system in which UHPLC (Dionex Ultimate 3000RS) system was equipped with Mass Spectrometer (Q-Exactive Orbitrap, Thermo, USA). Before the analysis extracts were filtered through 0.22 µm PTFE filter membrane (Labex Ltd, Hungary). In order to get chromatographic separation, 2 µL of each sample was injected into the HPLC system equipped with reverse phase C-18 column (Accucore C18 (100 mm x 2.1, mm i. d., 2.6 µm, Thermo). Column was themostated at 25 °C (± 1 °C). The elution was carried out at a flow rate of 0.2 mL/min using gradient elution. The solvents used were water (A) and methanol (B). Both was acidified with 0.1 % formic acid. Elution was performed using following gradient: isocratic 5 % B (0-3 min), a linear gradient increasing from 5% B to 100% (3-43 min), 100% B (43-61 min), a linear gradient decreasing from 100% B to 5% (61-62 min) and 5 % B (62-70 min). The total run time of analysis was 70 minutes.

The Thermo Q-Exactive Orbitrap mass spectrometer equipped with electrospray ionization source was in positive or negative polarity at the resolving power of 70,000 (full MS, range: m/z 100-1500) and 35000 (ddMS2). The ESI source parameters include ion spray voltage 4.0 kV in positive and 3.8 kV in negative mode; capillary temperature 320 °C; S-lens RF level 50 V; auxiliary gas: N2 (purity > 95%), heater temperature 300 °C. The data was acquired in full MS-ddMS2 mode by using Xcalibur 3.1 software. The full MS-ddMS^2 mode provided a full MS with MS/MS spectrum simultaneously in a single LC run. The full MS spectrum provided information about the intact molecular ion (e.g., M+, [M + H] + , [M - H] -), while the ddMS2 discovery generates the product ion spectra. The acquired data was processed by using TraceFinder 3.1 software (Thermo Fisher Scientific). All detailed analytical conditions have been also published [3]. Two runs were made with each sample, spectra were recorded separately in positive and negative mode. This method helped the identification of compounds in comparison with databases and peak identification was also based on the comparision the chromatographic data with standards, the exact molecular mass/adducts, fragmentation patterns, isotopic distributions and comparision with own tandem mass spectral library (MS/MS). As can be seen from the data in Tables S1-S12 a large number of components, structural isomers were tentatively identified. All samples were measured in both positive and negative ionization mode, the data recorded in the negative mode were more suitable for identifying the components.

Table S1. Chemical composition of the ethyl acetate extract of *P. fructicosa*

No.	Name	Formula	Rt	$[M + H]^+$	$[M - H]^-$	Fragment 1	Fragment 2	Fragment 3	Fragment 4	Fragment 5	Literature
1	Quinic acid	C7H12O6	2,13		191,05557	173,0440	171,0285	127,0386	111,0435	85,0278	
2	Neochlorogenic acid (5-O-Caffeoylquinic acid)	C16H18O9	13,59	355,10291		163,0387	145,0279	135,0443	117,0337	89,0390	[4]
3	Decaffeoylverbascoside	C20H30O12	14,56		461,16591	315,1088	297,0979	161,0448	135,0438	113,0228	[4]
4	Uralenneoside	C12H14O8	14,75		285,06105	153,0184	152,0101	109,0277	108,0199		
5	Genipin or isomer	C11H14O5	15,95	227,09195		209,0806	191,0701	177,0544	149,0595	121,0648	
6[1]	Chlorogenic acid (3-O-Caffeoylquinic acid)	C16H18O9	17,31	355,10291		163,0387	145,0282	135,0440	117,0336	89,0388	[4]
7[1]	Caffeic acid	C9H8O4	17,49		179,03444	135,0437					
8	Genipin or isomer	C11H14O5	18,00	227,09195		209,0806	191,0701	177,0544	149,0596	121,0649	
9[1]	Chryptochlorogenic acid (4-O-Caffeoylquinic acid)	C16H18O9	18,23	355,10291		163,0387	145,0284	135,0441	117,0336	89,0386	[4]
10	5-O-(p-Coumaroyl)quinic acid	C16H18O8	19,96		337,09235	191,0552	173,0439	163,0388	119,0486		[4]
11[1]	p-Coumaric acid	C9H8O3	20,69		163,03952	119,0486					
12	Campneoside II (β-Hydroxverbascoside)	C29H36O16	21,00		639,19252	621,1812	529,1570	487,1464	459,1492	161,0230	[4]
13	5-O-(p-Coumaroyl)quinic acid cis isomer	C16H18O8	21,43		337,09235	191,0552	173,0446	163,0387	119,0485		
14[1]	Ferulic acid	C10H10O4	21,84		193,05009	178,0261	149,0596	137,0234	134,0360		
15	Eriodictyol-O-hexoside	C21H22O11	22,21		449,10839	287,0561	151,0022	135,0437	107,0121		
16	Pentahydroxy(iso)flavone-O-hexoside	C21H20O12	23,15		463,08765	301,0359	300,0271	271,0245	243,0305	228,0417	
17	Forsythoside B	C34H44O19	23,40		755,23986	593,2086	461,1666	447,1512	315,1089	161,0230	[5]
18	Verbascoside (Acteoside)	C29H36O15	23,74		623,19760	461,1667	315,1090	179,0336	161,0230	133,0281	[5]
19	Luteolin-O-pentosylhexoside	C26H28O15	24,27		579,13500	285,0404	284,0329	151,0021	133,0280		[4]
20	Prunin (Naringenin-7-O-glucoside)	C21H22O10	24,30		433,11347	271,0613	177,0178	151,0022	119,0486	107,0123	
21	Luteolin-O-rhamnosylhexoside	C27H30O15	24,56		593,15065	285,0403	284,0325	151,0025	133,0277		[4]
22	Unidentified syringic acid derivative	C28H36O15	24,63		611,19760	413,1454	197,0446	182,0210	153,0543	121,0279	

23[1]	Luteolin-7-O-glucoside (Cynaroside)	C21H20O11	24,65		447,09274	327,0491	285,0403	284,0325	256,0375	133,0282	[5]
24	Alyssonoside	C35H46O19	24,87		769,25550	593,2091	575,1981	461,1664	175,0388	161,0230	[5]
25	Isoverbascoside	C29H36O15	25,11		623,19760	461,1663	315,1089	179,0342	161,0230	135,0438	[4]
26[1]	Isoquercitrin (Quercetin-3-O-glucoside)	C21H20O12	25,30		463,08765	301,0346	300,0271	271,0251	255,0302	151,0024	
27	Leucosceptoside A or Plantainoside C	C30H38O15	25,45		637,21325	461,1664	443,1535	315,1086	193,0495	175,0388	[4]
28	Methoxy-tetrahydroxy(iso)flavone-O-hexoside	C22H22O12	25,46		477,10330	462,0803	315,0512	313,0351	300,0272	299,0195	
29	Chrysoeriol-O-pentosylhexoside	C27H30O15	25,96		593,15065	299,0557	297,0389	285,0406	284,0335		
30	Apigenin-O-rhamnosylhexoside	C27H30O14	26,06		577,15574	269,0454	268,0374	151,0023	117,0325		
31	Unidentified phenylethanoid	C30H38O15	26,11		637,21325	475,1829	179,0341	161,0230	133,0278	113,0229	
32	Chrysoeriol-O-rhamnosylhexoside	C28H32O15	26,23		607,16630	299,0558	297,0396	284,0325	283,0239	255,0293	[5]
33[1]	Cosmosiin (Apigenin-7-O-glucoside)	C21H20O10	26,25	433,11347		271,0597	153,0181	119,0491			[4]
34	Chrysoeriol-7-O-glucoside	C22H22O11	26,51		461,10839	446,0852	299,0558	298,0481	283,0246	255,0295	[5]
35	Methoxy-trihydroxy(iso)flavone-O-hexoside	C22H22O11	26,94		461,10839	446,0855	298,0481	297,0402	283,0246	255,0294	
36[1]	Eriodictyol (3′,4′,5,7-Tetrahydroxyflavanone)	C15H12O6	26,97		287,05556	269,0457	151,0022	135,0437	125,0230	107,0122	[5]
37	Leucosceptoside A or Plantainoside C	C30H38O15	26,98		637,21325	461,1651	223,0606	193,0492	175,0387	161,0229	[4]
38	Tricin-7-O-glucoside	C23H24O12	27,15		491,11896	476,0956	327,0508	313,0353	298,0117	285,0403	[6]
39[1]	Isorhamnetin-3-O-glucoside	C22H22O12	27,22		477,10330	315,0484	314,0432	299,0189	285,0391	271,0244	
40	Martynoside	C31H40O15	27,68		651,22890	475,1836	193,0496	160,0152	134,0359	113,0228	[4]
41[1]	Naringenin (4',5,7-Trihydroxyflavanone)	C15H12O5	29,24		271,06065	227,0711	177,0182	151,0022	119,0486	107,0123	[5]
42	Homoeriodictyol (3'-Methoxy-4',5,7-trihydroxyflavanone)	C16H14O6	29,28		301,07122	196,0005	177,0182	151,0022	149,0594	134,0359	
43[1]	Hesperetin (4'-Methoxy-3',5,7-trihydroxyflavanone)	C16H14O6	29,81	303,08686		285,0763	177,0545	171,0287	153,0181	145,0284	[6]
44[1]	Luteolin (3',4',5,7-Tetrahydroxyflavone)	C15H10O6	30,31		285,03991	217,0499	199,0392	175,0388	151,0022	133,0280	
45	Apigenin-O-(E-p-coumaroyl)glucoside	C30H26O12	31,44		577,13461	431,0981	413,0885	269,0455	268,0377	145,0281	[4]
46	Chrysoeriol-7-O-(3-Z-p-coumaroyl)glucose	C31H28O13	31,60		607,14517	461,1092	299,0560	284,0325	255,0295	145,0281	[6]
47[1]	Apigenin (4',5,7-Trihydroxyflavone)	C15H10O5	32,06		269,04500	227,0347	225,0549	151,0023	149,0231	117,0329	[4]
48[1]	Chrysoeriol (3'-Methoxy-4',5,7-trihydroxyflavone)	C16H12O6	32,28		299,05556	284,0326	256,0372	227,0339	151,0025	107,0123	[6]
49	Dimethoxy-trihydroxy(iso)flavone	C17H14O7	32,35	331,08178		316,0572	301,0337	273,0387			

50	Chrysoeriol-7-O-(3-E-p-coumaroyl)glucose	C31H28O13	32,55		607,14517	461,1106	299,0560	284,0326	255,0294	145,0282	[6]
51	Apigenin-O-(Z-p-coumaroyl)glucoside	C30H26O12	32,56		577,13461	431,1022	413,0881	269,0454	268,0382	145,0282	[4]
52	Dihydroxy-dimethoxy(iso)flavone isomer 1	C17H14O6	33,47		313,07122	298,0481	297,0402	283,0247	269,0452	255,0294	
53	Dihydroxy-trimethoxy(iso)flavone isomer 1	C18H16O7	33,56		343,08178	328,0588	313,0355	298,0119	285,0405	270,0174	
54	Dihydroxy-dimethoxy(iso)flavone isomer 2	C17H14O6	34,00		313,07122	298,0481	297,0403	283,0248	269,0455	255,0295	
55	Dihydroxy-trimethoxy(iso)flavone isomer 2	C18H16O7	34,06		343,08178	328,0587	313,0354	298,0118	285,0400	270,0171	
56	Hydroxy-tetramethoxy(iso)flavone	C19H18O7	35,04	359,11308		344,0888	343,0809	329,0656	326,0781	298,0831	
57	Hydroxy-trimethoxy(iso)flavone isomer 1	C18H16O6	36,67	329,10252		314,0782	296,0676	268,0725			
58	Hydroxy-trimethoxy(iso)flavone isomer 2	C18H16O6	37,41	329,10252		314,0780	313,0702	300,0626	299,0545	285,0754	
59	Phytosphingosine	C18H39NO3	38,03	318,30082		300,2895	282,2787	264,2691	95,0856	60,0450	
60	Stearidonic acid	C18H28O2	40,82		275,20111	231,2117	59,0123				
61	Hydroxyoctadecatrienoic acid	C18H30O3	42,03		293,21167	277,2173	183,1379	155,1061			
62	cis-9,10-Epoxystearic acid	C18H34O3	43,01		297,24297	279,2327	171,1012	155,1063			
63[1]	α-Linolenic acid	C18H30O2	45,25		277,21676	259,2067	59,0121				
64[1]	Linoleic acid	C18H32O2	46,15		279,23241						
65	Linoleic acid isomer	C18H32O2	46,75		279,23241						
66[1]	Oleic acid	C18H34O2	47,11		281,24806						
67[1]	Stearic acid	C18H36O2	48,12		283,26371						
68[1]	Arachidic acid	C20H40O2	49,32		311,29501						
69	Lignoceric acid	C24H48O2	52,06		367,35761						
70	Cerotic acid	C26H52O2	53,80		395,38891						

[1] Confirmed by standard

Table S2. Chemical composition of the methanol extract of *P. fructicosa*

No.	Name	Formula	Rt	[M + H]$^+$	[M - H]$^-$	Fragment 1	Fragment 2	Fragment 3	Fragment 4	Fragment 5	Literature
1	Quinic acid	C7H12O6	2,01		191,05557	173,0437	171,0286	127,0387	111,0436	85,0278	
2	Citric acid	C6H8O7	2,94		191,01918	173,0446	171,0288	111,0072	87,0071	85,0279	
3[1]	Gallic acid (3,4,5-Trihydroxybenzoic acid)	C7H6O5	4,56		169,01370	125,0229	97,0278	69,0328			[4]
4	Protocatechuic acid (3,4-Dihydroxybenzoic acid)	C7H6O4	9,06		153,01879	109,0279	108,0200	91,0174	81,0328		[4]
5	p-Hydroxybenzoic acid	C7H6O3	12,50		137,02387	109,0283					[4]
6	Lamiide	C17H26O12	13,23		467.14008[2]	421,1359	403,1242	385,1137	259,0819	101,0229	[5]
7[1]	Neochlorogenic acid (5-O-Caffeoylquinic acid)	C16H18O9	13,56	355,10291		163,0388	145,0283	135,0440	117,0337	89,0388	[4]
8	Vanillic acid-4-O-pentosylhexoside	C19H26O13	13,58		461,12952	329,0890	167,0337	152,0101	123,0436	108,0201	[4]
9	Decaffeoylverbascoside	C20H30O12	14,52		461,16591	315,1086	297,0978	161,0446	135,0437	113,0228	[4]
10	Uralenneoside	C12H14O8	14,73		285,06105	153,0179	152,0101	109,0279	108,0201		
11	2-Isopropylmalic acid	C7H12O5	14,98		175,06065	157,0492	131,0704	115,0384	113,0592	85,0642	
12	3-O-(p-Coumaroyl)quinic acid cis isomer	C16H18O8	15,46		337,09235	191,0554	173,0441	163,0389	119,0486		
13	Swertiamacroside	C21H28O13	15,53		487,14517	179,0339	161,0231	135,0438	113,0229	85,0278	[4]
14	Unidentified iridoid	C17H26O12	15,89		467.14008[2]	403,1244	385,1135	331,1049	223,0604	179,0550	
15	3-O-(p-Coumaroyl)quinic acid	C16H18O8	15,90		337,09235	191,0547	173,0440	163,0387	119,0487		
16	Vanillic acid (4-Hydroxy-3-methoxybenzoic acid)	C8H8O4	16,83		167,03444	152,0101	123,0438	108,0202			
17	Caffeic acid-O-hexoside	C15H18O9	16,90		341,08726	179,0339	135,0438				[4]
18[1]	Chlorogenic acid (3-O-Caffeoylquinic acid)	C16H18O9	17,27	355,10291		163,0388	145,0284	135,0441	117,0336	89,0389	[4]
19[1]	Caffeic acid	C9H8O4	17,55		179,03444	135,0437					
20	Genipin or isomer	C11H14O5	17,96	227,09195		209,0806	191,0701	177,0544	149,0596	121,0649	
21[1]	Chryptochlorogenic acid (4-O-Caffeoylquinic acid)	C16H18O9	18,21	355,10291		163,0387	145,0283	135,0440	117,0337	89,0387	[4]
22	Syringic acid	C9H10O5	18,60		197,04500	182,0212	166,9972	153,0539	138,0306	123,0070	
23	5-O-(p-Coumaroyl)quinic acid	C16H18O8	19,88		337,09235	191,0552	173,0445	163,0387	119,0489		[4]
24[1]	p-Coumaric acid	C9H8O3	20,72		163,03952	119,0487					
25	Vicenin-2 (Apigenin-6,8-di-C-glucoside)	C27H30O15	20,91	595,16630		577,1536	541,1309	457,1126	325,0702	295,0586	[6]

26	Campneoside II (β-Hydroxverbascoside)	C29H36O16	21,01		639,19252	621,1821	529,1572	487,1466	459,1521	161,0230	[4]
27	5-O-(p-Coumaroyl)quinic acid cis isomer	C16H18O8	21,44		337,09235	191,0550	173,0443	163,0385	119,0485		
28[1]	Ferulic acid	C10H10O4	21,88		193,05009	178,0262	149,0592	137,0228	134,0361		
29	Eriodictyol-O-hexoside	C21H22O11	22,22		449,10839	287,0559	151,0022	135,0437	107,0123		
30	Pentahydroxy(iso)flavone-O-hexoside	C21H20O12	23,14		463,08765	301,0353	300,0274	271,0247	243,0308	228,0420	
31	Forsythoside B	C34H44O19	23,38		755,23986	593,2086	461,1671	447,1520	315,1088	161,0230	[5]
32	Luteolin-O-hexosylhexoside	C27H30O16	23,49		609,14557	447,0941	285,0404	284,0328	151,0021		
33	Verbascoside (Acteoside)	C29H36O15	23,73		623,19760	461,1665	315,1085	179,0336	161,0230	133,0281	[5]
34	Luteolin-O-pentosylhexoside	C26H28O15	24,26		579,13500	285,0403	284,0325	151,0020	133,0278		[4]
35	Prunin (Naringenin-7-O-glucoside)	C21H22O10	24,28		433,11347	271,0610	177,0182	151,0022	119,0486	107,0122	
36	Luteolin-O-rhamnosylhexoside	C27H30O15	24,54		593,15065	285,0403	284,0326	151,0019	133,0281		[4]
37[1]	Luteolin-7-O-glucoside (Cynaroside)	C21H20O11	24,63		447,09274	327,0508	285,0404	284,0325	256,0379	133,0278	[6]
38	Unidentified syringic acid derivative	C28H36O15	24,63		611,19760	413,1454	197,0446	182,0210	153,0543	121,0279	
39	Dicaffeoylquinic acid isomer 2	C25H24O12	24,69		515,11896	353,0876	335,0799	191,0552	179,0339	135,0437	
40	Alyssonoside	C35H46O19	24,87		769,25550	593,2086	575,1986	461,1658	175,0389	161,0230	[5]
41	Isoverbascoside	C29H36O15	25,10		623,19760	461,1666	315,1088	179,0339	161,0230	135,0438	[4]
42[1]	Isoquercitrin (Quercetin-3-O-glucoside)	C21H20O12	25,28		463,08765	301,0357	300,0274	271,0245	255,0296	151,0026	
43	Leucosceptoside A or Plantainoside C	C30H38O15	25,44		637,21325	461,1664	443,1561	315,1074	193,0496	175,0388	[4]
44	Methoxy-tetrahydroxy(iso)flavone-O-hexoside	C22H22O12	25,45		477,10330	462,0789	315,0510	313,0357	300,0274	299,0197	
45	Apigenin-O-pentosylhexoside	C26H28O14	25,76		565,15574	269,0455	113,0226				
46	Chrysoeriol-O-pentosylhexoside	C27H30O15	25,94		593,15065	299,0559	297,0387	285,0403	284,0326		
47	Apigenin-O-rhamnosylhexoside	C27H30O14	26,05		577,15574	269,0454	268,0375	151,0024	117,0327		
48	Unidentified phenylethanoid	C30H38O15	26,10		637,21325	475,1840	179,0340	161,0230	133,0281	113,0229	
49	Chrysoeriol-O-rhamnosylhexoside	C28H32O15	26,21		607,16630	299,0558	297,0408	284,0324	283,0253	255,0293	[6]
50[1]	Cosmosiin (Apigenin-7-O-glucoside)	C21H20O10	26,24	433,11347		271,0597	153,0179	119,0492			[4]
51	Dicaffeoylquinic acid isomer 3	C25H24O12	26,43		515,11896	353,0874	191,0551	179,0338	173,0442	135,0437	
52	Chrysoeriol-7-O-glucoside	C22H22O11	26,50		461,10839	446,0854	299,0560	298,0480	283,0247	255,0295	[6]
53	Methoxy-trihydroxy(iso)flavone-O-hexoside	C22H22O11	26,92		461,10839	446,0849	298,0481	297,0403	283,0247	255,0296	
54[1]	Eriodictyol (3′,4′,5,7-Tetrahydroxyflavanone)	C15H12O6	26,95		287,05556	269,0464	151,0022	135,0437	125,0227	107,0122	[6]
55	Leucosceptoside A or Plantainoside C	C30H38O15	26,97		637,21325	461,1674	223,0604	193,0503	175,0390	161,0231	[4]
56	Caffeoyl-hydroxybenzoylhexose	C22H22O11	27,09		461,10839	323,0770	283,0247	179,0339	161,0230	137,0230	

57	Tricin-7-O-glucoside	C23H24O12	27,14		491,11896	476,0959	327,0505	313,0353	298,0121	285,0417	[6]
58[1]	Isorhamnetin-3-O-glucoside	C22H22O12	27,21		477,10330	315,0511	314,0429	299,0193	285,0409	271,0251	
59	Abscisic acid	C15H20O4	27,27		263,12834	219,1388	204,1148	201,1276	152,0828	151,0749	
60	Martynoside	C31H40O15	27,67		651,22890	475,1818	193,0497	160,0152	134,0359	113,0228	[4]
61[1]	Naringenin (4',5,7-Trihydroxyflavanone)	C15H12O5	29,23		271,06065	227,0702	177,0182	151,0023	119,0486	107,0123	[6]
62	Homoeriodictyol (3'-Methoxy-4',5,7-trihydroxyflavanone)	C16H14O6	29,27		301,07122	196,0006	177,0182	151,0022	149,0594	134,0360	
63	Lubiminol or Canusesnol I	C15H26O2	29,43	239,20111		221,1896	203,1795	163,1480	135,1169	95,0859	
64[1]	Hesperetin (4'-Methoxy-3',5,7-trihydroxyflavanone)	C16H14O6	29,81	303,08686		285,0761	177,0545	171,0287	153,0181	145,0283	[6]
65	Lubiminol or Canusesnol I	C15H26O2	30,30	239,20111		221,1899	203,1795	163,1479	135,1168	95,0858	
66[1]	Luteolin (3',4',5,7-Tetrahydroxyflavone)	C15H10O6	30,30		285,03991	217,0500	199,0393	175,0388	151,0023	133,0281	
67	Luteolin-O-(Z-p-coumaroyl)glucoside	C30H26O13	30,40		593,12952	447,0923	285,0403	284,0326	151,0021	145,0281	[6]
68	Apigenin-O-(E-p-coumaroyl)glucoside	C30H26O12	31,44		577,13461	431,0981	413,0873	269,0454	268,0376	145,0281	[4]
69	Luteolin-O-(E-p-coumaroyl)glucoside	C30H26O13	31,50		593,12952	447,0925	285,0405	284,0330	151,0022	145,0283	[6]
70	Chrysoeriol-7-O-(3-Z-p-coumaroyl)glucose	C31H28O13	31,59		607,14517	461,1082	299,0560	284,0325	255,0300	145,0281	[6]
71[1]	Apigenin (4',5,7-Trihydroxyflavone)	C15H10O5	32,05		269,04500	227,0333	225,0549	151,0023	149,0230	117,0329	[4]
72[1]	Chrysoeriol (3'-Methoxy-4',5,7-trihydroxyflavone)	C16H12O6	32,27		299,05556	284,0325	256,0374	227,0342	151,0022	107,0118	[6]
73	Dimethoxy-trihydroxy(iso)flavone	C17H14O7	32,35	331,08178		316,0573	301,0344	273,0384			
74	Chrysoeriol-7-O-(3-E-p-coumaroyl)glucose	C31H28O13	32,53		607,14517	461,1086	299,0559	284,0325	255,0296	145,0281	[6]
75	Apigenin-O-(Z-p-coumaroyl)glucoside	C30H26O12	32,57		577,13461	431,0981	413,0871	269,0454	268,0378	145,0281	[4]
76	Dihydroxy-dimethoxy(iso)flavone isomer 1	C17H14O6	33,47		313,07122	298,0479	297,0407	283,0248	269,0463	255,0292	
77	Dihydroxy-trimethoxy(iso)flavone isomer 1	C18H16O7	33,55		343,08178	328,0579	313,0349	298,0124	285,0399	270,0180	
78	Dihydroxy-dimethoxy(iso)flavone isomer 2	C17H14O6	34,00		313,07122	298,0482	297,0403	283,0247	269,0454	255,0295	
79	Dihydroxy-trimethoxy(iso)flavone isomer 2	C18H16O7	34,05		343,08178	328,0588	313,0355	298,0122	285,0402	270,0169	
80	Hydroxy-tetramethoxy(iso)flavone	C19H18O7	35,03	359,11308		344,0885	343,0808	329,0658	326,0781	298,0832	
81	Hydroxy-trimethoxy(iso)flavone isomer 1	C18H16O6	36,66	329,10252		314,0782	296,0677	268,0727			
82	Hydroxy-trimethoxy(iso)flavone isomer 2	C18H16O6	37,40	329,10252		314,0778	313,0703	300,0623	299,0555	285,0756	
83	Phytosphingosine	C18H39NO3	38,01	318,30082		300,2887	282,2789	264,2691	95,0859	60,0450	
84[1]	α-Linolenic acid	C18H30O2	45,24		277,21676						
85[1]	Linoleic acid	C18H32O2	46,15		279,23241						

[1] Confirmed by standard

[2] Formate adduct

Table S3. Chemical composition of the methanol/water extract of *P. fructicosa*

No.	Name	Formula	Rt	[M + H]+	[M - H]-	Fragment 1	Fragment 2	Fragment 3	Fragment 4	Fragment 5	Literature
1	Quinic acid	C7H12O6	2,01		191,05557	173,0443	171,0289	127,0390	111,0438	85,0280	
2	Citric acid	C6H8O7	2,89		191,01918	173,0455	171,0289	111,0073	87,0072	85,0280	
3[1]	Gallic acid (3,4,5-Trihydroxybenzoic acid)	C7H6O5	4,45		169,01370	125,0231	97,0279	69,0331			[4]
4	Protocatechuic acid (3,4-Dihydroxybenzoic acid)	C7H6O4	8,78		153,01879	109,0281	108,0200	91,0177	81,0329		[4]
5	Lamiide	C17H26O12	13,11		467.14008[2]	421,1346	403,1249	385,1139	259,0825	101,0230	[5]
6[1]	Neochlorogenic acid (5-O-Caffeoylquinic acid)	C16H18O9	13,36	355,10291		163,0390	145,0286	135,0443	117,0339	89,0391	[4]
7	Vanillic acid-4-O-pentosylhexoside	C19H26O13	13,45		461,12952	329,0873	167,0339	152,0103	123,0436	108,0204	[4]
8	Decaffeoylverbascoside	C20H30O12	14,40		461,16591	315,1092	297,0986	161,0445	135,0439	113,0230	[4]
9	Uralenneoside	C12H14O8	14,69		285,06105	153,0181	152,0103	109,0280	108,0202		
10	2-Isopropylmalic acid	C7H12O5	14,84		175,06065	157,0495	131,0700	115,0387	113,0594	85,0643	
11	Swertiamacroside	C21H28O13	15,42		487,14517	179,0342	161,0233	135,0440	113,0230	85,0279	[4]
12	Unidentified iridoid	C17H26O12	15,80		467.14008[2]	403,1242	385,1146	331,1034	223,0608	179,0553	
13	Caffeic acid-O-hexoside	C15H18O9	16,84		341,08726	179,0342	135,0440				[4]
14[1]	Chlorogenic acid (3-O-Caffeoylquinic acid)	C16H18O9	17,07	355,10291		163,0390	145,0285	135,0443	117,0339	89,0391	[4]
15[1]	Caffeic acid	C9H8O4	17,39		179,03444	135,0439					
16	Genipin or isomer	C11H14O5	17,89	227,09195		209,0810	191,0705	177,0547	149,0598	121,0651	
17[1]	Chryptochlorogenic acid (4-O-Caffeoylquinic acid)	C16H18O9	18,12	355,10291		163,0390	145,0285	135,0443	117,0337	89,0390	[4]
18[1]	p-Coumaric acid	C9H8O3	20,59		163,03952	119,0488					
19	Vicenin-2 (Apigenin-6,8-di-C-glucoside)	C27H30O15	20,76	595,16630		577,1534	541,1334	457,1130	325,0707	295,0613	[6]
20	Campneoside II (β-Hydroxverbascoside)	C29H36O16	20,91		639,19252	621,1831	529,1568	487,1463	459,1508	161,0233	[4]
21	Eriodictyol-O-hexoside	C21H22O11	22,12		449,10839	287,0547	151,0025	135,0439	107,0126		
22	Pentahydroxy(iso)flavone-O-hexoside	C21H20O12	23,05		463,08765	301,0358	300,0278	271,0244	243,0304	228,0423	
23	Luteolin-O-hexosylhexoside	C27H30O16	23,27		609,14557	447,0912	285,0408	284,0329	151,0023		
24	Forsythoside B	C34H44O19	23,29		755,23986	593,2091	461,1670	447,1515	315,1098	161,0233	[5]
25	Verbascoside (Acteoside)	C29H36O15	23,63		623,19760	461,1675	315,1096	179,0340	161,0233	133,0284	[5]
26	Luteolin-O-pentosylhexoside	C26H28O15	24,15		579,13500	285,0409	284,0331	151,0024	133,0281		[4]
27	Prunin (Naringenin-7-O-glucoside)	C21H22O10	24,16		433,11347	271,0616	177,0183	151,0025	119,0489	107,0124	
28	Dicaffeoylquinic acid isomer 1	C25H24O12	24,18		515,11896	353,0890	335,0760	191,0552	179,0344	173,0445	
29	Luteolin-O-rhamnosylhexoside	C27H30O15	24,44		593,15065	285,0408	284,0330	151,0019	133,0282		[4]

30[1]	Luteolin-7-O-glucoside (Cynaroside)	C21H20O11	24,52		447,09274	327,0512	285,0408	284,0329	256,0376	133,0283		[6]
31	Unidentified syringic acid derivative	C28H36O15	24,53		611,19760	413,1441	197,0449	182,0214	153,0545	121,0281		
32	Dicaffeoylquinic acid isomer 2	C25H24O12	24,57		515,11896	353,0883	335,0769	191,0555	179,0342	135,0440		
33	Alyssonoside	C35H46O19	24,76		769,25550	593,2102	575,2026	461,1652	175,0391	161,0233		[5]
34	Isoverbascoside	C29H36O15	24,99		623,19760	461,1671	315,1085	179,0342	161,0233	135,0440		[4]
35[1]	Isoquercitrin (Quercetin-3-O-glucoside)	C21H20O12	25,17		463,08765	301,0356	300,0280	271,0251	255,0296	151,0024		
36	Leucosceptoside A or Plantainoside C	C30H38O15	25,33		637,21325	461,1671	443,1535	315,1092	193,0499	175,0391		[4]
37	Methoxy-tetrahydroxy(iso)flavone-O-hexoside	C22H22O12	25,34		477,10330	462,0790	315,0517	313,0360	300,0279	299,0203		
38	Chrysoeriol-O-pentosylhexoside	C27H30O15	25,84		593,15065	299,0562	297,0384	285,0398	284,0329			
39	Apigenin-O-rhamnosylhexoside	C27H30O14	25,93		577,15574	269,0459	268,0383	151,0023	117,0331			
40	Unidentified phenylethanoid	C30H38O15	26,00		637,21325	475,1822	179,0339	161,0232	133,0283	113,0227		
41	Chrysoeriol-O-rhamnosylhexoside	C28H32O15	26,12		607,16630	299,0563	297,0418	284,0329	283,0256	255,0294		[6]
42[1]	Cosmosiin (Apigenin-7-O-glucoside)	C21H20O10	26,14	433,11347		271,0600	153,0183	119,0494				[4]
43	Dicaffeoylquinic acid isomer 3	C25H24O12	26,33		515,11896	353,0882	191,0554	179,0342	173,0445	135,0440		
44	Chrysoeriol-7-O-glucoside	C22H22O11	26,39		461,10839	446,0859	299,0566	298,0485	283,0252	255,0298		[6]
45[1]	Eriodictyol (3′,4′,5,7-Tetrahydroxyflavanone)	C15H12O6	26,82		287,05556	269,0456	151,0025	135,0440	125,0232	107,0125		[6]
46	Leucosceptoside A or Plantainoside C	C30H38O15	26,88		637,21325	461,1662	223,0601	193,0500	175,0390	161,0234		[4]
47	Caffeoyl-hydroxybenzoylhexose	C22H22O11	26,95		461,10839	323,0776	283,0251	179,0344	161,0233	137,0232		
48	Tricin-7-O-glucoside	C23H24O12	27,04		491,11896	476,0981	327,0510	313,0358	298,0116	285,0411		[6]
49[1]	Isorhamnetin-3-O-glucoside	C22H22O12	27,11		477,10330	315,0515	314,0440	299,0195	285,0406	271,0241		
50	Martynoside	C31H40O15	27,58		651,22890	475,1834	193,0503	160,0154	134,0360	113,0233		[4]
51[1]	Naringenin (4',5,7-Trihydroxyflavanone)	C15H12O5	29,10		271,06065	227,0712	177,0185	151,0025	119,0489	107,0125		[6]
52	Homoeriodictyol (3'-Methoxy-4',5,7-trihydroxyflavanone)	C16H14O6	29,15		301,07122	196,0011	177,0182	151,0025	149,0597	134,0363		
53	Lubiminol or Canusesnol I	C15H26O2	29,30	239,20111		221,1897	203,1797	163,1479	135,1171	95,0863		
54[1]	Hesperetin (4'-Methoxy-3',5,7-trihydroxyflavanone)	C16H14O6	29,66	303,08686		285,0763	177,0546	171,0289	153,0183	145,0286		[6]
55	Lubiminol or Canusesnol I	C15H26O2	30,17	239,20111		221,1894	203,1798	163,1476	135,1169	95,0860		
56[1]	Luteolin (3',4',5,7-Tetrahydroxyflavone)	C15H10O6	30,19		285,03991	217,0498	199,0394	175,0391	151,0024	133,0283		
57	Luteolin-O-(Z-p-coumaroyl)glucoside	C30H26O13	30,30		593,12952	447,0971	285,0409	284,0330	151,0023	145,0283		[6]
58	Apigenin-O-(E-p-coumaroyl)glucoside	C30H26O12	31,33		577,13461	431,0989	413,0880	269,0458	268,0381	145,0283		[4]
59	Luteolin-O-(E-p-coumaroyl)glucoside	C30H26O13	31,41		593,12952	447,0927	285,0411	284,0333	151,0022	145,0282		[6]
60	Chrysoeriol-7-O-(3-Z-p-coumaroyl)glucose	C31H28O13	31,50		607,14517	461,1098	299,0564	284,0330	255,0295	145,0283		[6]
61[1]	Apigenin (4',5,7-Trihydroxyflavone)	C15H10O5	31,94		269,04500	227,0341	225,0554	151,0025	149,0232	117,0331		[4]
62[1]	Chrysoeriol (3'-Methoxy-4',5,7-trihydroxyflavone)	C16H12O6	32,18		299,05556	284,0329	256,0377	227,0338	151,0025	107,0117		[6]
63	Dimethoxy-trihydroxy(iso)flavone	C17H14O7	32,21	331,08178		316,0582	301,0343	273,0398				
64	Chrysoeriol-7-O-(3-E-p-coumaroyl)glucose	C31H28O13	32,46		607,14517	461,1092	299,0564	284,0329	255,0296	145,0283		[6]
65	Apigenin-O-(Z-p-coumaroyl)glucoside	C30H26O12	32,47		577,13461	431,0984	413,0870	269,0458	268,0379	145,0284		[4]

66	Dihydroxy-dimethoxy(iso)flavone isomer 1	$C_{17}H_{14}O_6$	33,38		313,07122	298,0481	297,0411	283,0257	269,0462	255,0290	
67	Dihydroxy-dimethoxy(iso)flavone isomer 2	$C_{17}H_{14}O_6$	33,92		313,07122	298,0483	297,0407	283,0250	269,0464	255,0296	
68	Hydroxy-tetramethoxy(iso)flavone	$C_{19}H_{18}O_7$	34,92	359,11308		344,0886	343,0810	329,0661	326,0786	298,0837	
69	Hydroxy-trimethoxy(iso)flavone isomer 1	$C_{18}H_{16}O_6$	36,56	329,10252		314,0780	296,0678	268,0726			
70	Hydroxy-trimethoxy(iso)flavone isomer 2	$C_{18}H_{16}O_6$	37,27	329,10252		314,0773	313,0707	300,0633	299,0552	285,0762	
71[1]	α-Linolenic acid	$C_{18}H_{30}O_2$	45,19		277,21676						
72[1]	Linoleic acid	$C_{18}H_{32}O_2$	46,08		279,23241						

[1] Confirmed by standard

[2] Formate adduct

Table S4. Chemical composition of the water extract of *P. fructicosa*

No.	Name	Formula	Rt	[M + H]+	[M - H]-	Fragment 1	Fragment 2	Fragment 3	Fragment 4	Fragment 5	Literature
1	Quinic acid	C7H12O6	2,10		191,05557	173,0443	171,0282	127,0387	111,0436	85,0279	
2	Citric acid	C6H8O7	2,95		191,01918	173,0441	171,0288	111,0073	87,0072	85,0279	
3	Protocatechuic acid (3,4-Dihydroxybenzoic acid)	C7H6O4	8,86		153,01879	109,0280	108,0202	91,0173	81,0331		[4]
4	Lamiide	C17H26O12	13,16		467.14008[2]	421,1347	403,1242	385,1138	259,0821	101,0230	[6]
5[1]	Neochlorogenic acid (5-O-Caffeoylquinic acid)	C16H18O9	13,27	355,10291		163,0389	145,0284	135,0441	117,0337	89,0390	[4]
6	Vanillic acid-4-O-pentosylhexoside	C19H26O13	13,53		461,12952	329,0893	167,0338	152,0102	123,0437	108,0202	[4]
7	Decaffeoylverbascoside	C20H30O12	14,46		461,16591	315,1093	297,0980	161,0442	135,0438	113,0229	[4]
8	Uralenneoside	C12H14O8	14,72		285,06105	153,0180	152,0102	109,0280	108,0202		
9	2-Isopropylmalic acid	C7H12O5	14,89		175,06065	157,0494	131,0702	115,0386	113,0593	85,0643	
10	3-O-(p-Coumaroyl)quinic acid cis isomer	C16H18O8	15,10		337,09235	191,0556	173,0440	163,0389	119,0486		
11	Swertiamacroside	C21H28O13	15,50		487,14517	179,0340	161,0230	135,0439	113,0230	85,0278	[4]
12	3-O-(p-Coumaroyl)quinic acid	C16H18O8	15,65		337,09235	191,0554	173,0441	163,0388	119,0487		
13	Unidentified iridoid	C17H26O12	15,85		467.14008[2]	403,1239	385,1151	331,1035	223,0606	179,0552	
14	Caffeic acid-O-hexoside	C15H18O9	16,92		341,08726	179,0340	135,0438				[4]
15[1]	Chlorogenic acid (3-O-Caffeoylquinic acid)	C16H18O9	17,11	355,10291		163,0390	145,0285	135,0443	117,0338	89,0390	[4]
16[1]	Caffeic acid	C9H8O4	17,45		179,03444	135,0438					
17	Genipin or isomer	C11H14O5	17,94	227,09195		209,0810	191,0704	177,0547	149,0598	121,0651	
18[1]	Chryptochlorogenic acid (4-O-Caffeoylquinic acid)	C16H18O9	18,05	355,10291		163,0389	145,0284	135,0442	117,0339	89,0390	[4]
19	4-O-(p-Coumaroyl)quinic acid cis isomer	C16H18O8	18,19		337,09235	191,0544	173,0443	163,0391	119,0488		
20	5-O-(p-Coumaroyl)quinic acid	C16H18O8	19,67		337,09235	191,0553	173,0445	163,0389	119,0488		[4]
21	4-O-(p-Coumaroyl)quinic acid	C16H18O8	19,91		337,09235	191,0553	173,0443	163,0389	119,0487		
22[1]	p-Coumaric acid	C9H8O3	20,65		163,03952	119,0487					
23	Vicenin-2 (Apigenin-6,8-di-C-glucoside)	C27H30O15	20,84	595,16630		577,1494	541,1325	457,1139	325,0713	295,0596	[6]
24	Campneoside II (β-Hydroxverbascoside)	C29H36O16	20,96		639,19252	621,1832	529,1566	487,1473	459,1515	161,0232	[4]
25	5-O-(p-Coumaroyl)quinic acid cis isomer	C16H18O8	21,26		337,09235	191,0553	173,0443	163,0384	119,0482		

26[1]	Ferulic acid	C10H10O4	21,82		193,05009	178,0256	149,0593	137,0229	134,0360		
27	Campneoside II (β-Hydroxverbascoside) isomer 1	C29H36O16	22,42		639,19252	621,1830	529,1564	487,1465	459,1505	161,0232	
28	Campneoside II (β-Hydroxverbascoside) isomer 2	C29H36O16	22,72		639,19252	621,1827	529,1578	487,1451	459,1519	161,0232	
29	Pentahydroxy(iso)flavone-O-hexoside	C21H20O12	23,09		463,08765	301,0360	300,0280	271,0245	243,0304	228,0421	
30	Forsythoside B	C34H44O19	23,34		755,23986	593,2092	461,1661	447,1509	315,1103	161,0231	[5]
31	Luteolin-O-hexosylhexoside	C27H30O16	23,42		609,14557	447,0925	285,0407	284,0328	151,0022		
32	Verbascoside (Acteoside)	C29H36O15	23,70		623,19760	461,1669	315,1101	179,0338	161,0231	133,0282	[5]
33	Luteolin-O-pentosylhexoside	C26H28O15	24,21		579,13500	285,0406	284,0328	151,0028	133,0280		[4]
34	Dicaffeoylquinic acid isomer 1	C25H24O12	24,27		515,11896	353,0866	335,0739	191,0552	179,0341	173,0442	
35	Luteolin-O-rhamnosylhexoside	C27H30O15	24,50		593,15065	285,0406	284,0328	151,0021	133,0278		[4]
36	Dicaffeoylquinic acid isomer 2	C25H24O12	24,53		515,11896	353,0880	335,0771	191,0553	179,0340	135,0440	
37[1]	Luteolin-7-O-glucoside (Cynaroside)	C21H20O11	24,58		447,09274	327,0502	285,0407	284,0332	256,0380	133,0278	[6]
38	Unidentified syringic acid derivative	C28H36O15	24,60		611,19760	413,1456	197,0447	182,0213	153,0544	121,0280	
39	Alyssonoside	C35H46O19	24,82		769,25550	593,2061	575,1984	461,1655	175,0391	161,0232	[5]
40	Isoverbascoside	C29H36O15	25,04		623,19760	461,1668	315,1088	179,0340	161,0232	135,0439	[4]
41	Methoxy-tetrahydroxy(iso)flavone-O-hexoside	C22H22O12	25,40		477,10330	462,0787	315,0505	313,0356	300,0289	299,0192	
42	Chrysoeriol-O-pentosylhexoside	C27H30O15	25,91		593,15065	299,0561	297,0385	285,0400	284,0321		
43	Apigenin-O-rhamnosylhexoside	C27H30O14	25,98		577,15574	269,0457	268,0372	151,0023	117,0325		
44	Chrysoeriol-O-rhamnosylhexoside	C28H32O15	26,16		607,16630	299,0562	297,0406	284,0328	283,0249	255,0288	[6]
45	Dicaffeoylquinic acid isomer 3	C25H24O12	26,23		515,11896	353,0879	191,0552	179,0340	173,0444	135,0439	
46	Caffeoyl-hydroxybenzoylhexose	C22H22O11	27,01		461,10839	323,0774	283,0256	179,0340	161,0232	137,0231	
47[1]	Naringenin (4',5,7-Trihydroxyflavanone)	C15H12O5	29,17		271,06065	227,0704	177,0186	151,0025	119,0487	107,0123	[6]
48[1]	Luteolin (3',4',5,7-Tetrahydroxyflavone)	C15H10O6	30,27		285,03991	217,0498	199,0391	175,0387	151,0023	133,0284	
49[1]	Apigenin (4',5,7-Trihydroxyflavone)	C15H10O5	32,02		269,04500	227,0332	225,0548	151,0022	149,0228	117,0332	[4]
50[1]	Chrysoeriol (3'-Methoxy-4',5,7-trihydroxyflavone)	C16H12O6	32,21		299,05556	284,0328	256,0379	227,0335	151,0024	107,0119	[6]

[1] Confirmed by standard

[2] Formate adduct

Table S5. Chemical composition of the ethyl acetate extract of *P. herba-venti*

No.	Name	Formula	Rt	$[M + H]^+$	$[M - H]^-$	Fragment 1	Fragment 2	Fragment 3	Fragment 4	Fragment 5	Literature
1	Quinic acid	C7H12O6	2,08		191,05557	173,0443	171,0288	127,0388	111,0436	85,0279	
2	Citric acid	C6H8O7	2,92		191,01918	173,0079	171,0285	111,0073	87,0071	85,0279	
3	Unidentified iridoid	C17H26O12	8,67		467.14008[2]	403,1243	385,1149	331,1029	223,0605	89,0228	
4	Unidentified iridoid	C17H26O12	15,88		467.14008[2]	403,1246	385,1138	331,1033	223,0606	179,0552	
5[1]	Chlorogenic acid (3-O-Caffeoylquinic acid)	C16H18O9	17,14	355,10291		163,0390	145,0285	135,0443	117,0338	89,0389	
6[1]	Caffeic acid	C9H8O4	17,45		179,03444	135,0438	107,0489				
7	5-O-(4-Coumaroyl)quinic acid	C16H18O8	19,68		337,09235	191,0553	173,0444	163,0383	119,0487		
8	Caffeoylshikimic acid	C16H16O8	20,39		335,07670	179,0340	173,0446	161,0232	135,0438	111,0438	
9	5-O-Feruloylquinic acid	C17H20O9	20,53		367,10291	193,0496	191,0553	173,0443	134,0360		
10[1]	p-Coumaric acid	C9H8O3	20,63		163,03952	119,0487					
11	5-O-(4-Coumaroyl)quinic acid cis isomer	C16H18O8	21,28		337,09235	191,0553	173,0431	163,0385	119,0486		
12	5-O-Feruloylquinic acid cis isomer	C17H20O9	22,11		367,10291	193,0495	191,0552	173,0440	134,0359		
13	Pentahydroxy(iso)flavone-O-rhamnosylglucuronide	C27H28O17	22,43		623,12483	301,0357	300,0260	255,0300	227,0338		
14	Pentahydroxy(iso)flavone-O-hexoside	C21H20O12	23,13		463,08765	301,0360	300,0272	283,0244	255,0297	228,0420	
15	Forsythoside B	C34H44O19	23,35		755,23986	593,2090	461,1683	447,1507	315,1097	161,0232	[7]
16	Verbascoside (Acteoside)	C29H36O15	23,69		623,19760	461,1668	315,1085	179,0340	161,0231	133,0282	
17	Luteolin-O-rhamnosylglucuronide isomer 1	C27H28O16	23,97		607,12991	285,0406	284,0315	133,0278			
18	Forsythoside B isomer	C34H44O19	24,42		755,23986	593,2099	461,1674	315,1096	179,0340	161,0231	
19	Hattushoside	C28H36O15	24,59		611,19760	431,1580	413,1454	299,1140	197,0447	153,0544	[7]
20	Luteolin-O-crotonylhexoside	C25H24O12	24,59		515,11896	447,0937	285,0406	284,0324			
21[1]	Luteolin-7-O-glucoside (Cynaroside)	C21H20O11	24,60		447,09274	285,0406	284,0327	256,0378	151,0022	133,0284	
22	Alyssonoside	C35H46O19	24,81		769,25551	593,2089	575,1976	461,1684	447,1514	175,0389	[7]
23	Methoxy-tetrahydroxy(iso)flavone-O-hexoside isomer 1	C22H22O12	25,01		477,10330	462,0810	315,0501	314,0429	300,0280	299,0189	
24	Isoverbascoside	C29H36O15	25,05		623,19760	461,1663	315,1079	179,0337	161,0231	135,0439	
25	Luteolin-O-rhamnosylglucuronide isomer 2	C27H28O16	25,32		607,12991	285,0408	284,0327	151,0022	133,0278		

26	Leucosceptoside A	C30H38O15	25,40		637,21325	461,1670	443,1570	315,1075	175,0389	160,0153		
27	Methoxy-tetrahydroxy(iso)flavone-O-hexoside isomer 2	C22H22O12	25,40		477,10330	462,0796	315,0514	313,0350	300,0194	299,0194		
28	Alyssonoside isomer	C35H46O19	25,92		769,25551	593,2091	575,1956	461,1656	315,1087	175,0390		
29	Chrysoeriol-7-O-glucoside	C22H22O11	26,46		461,10839	446,0851	299,0551	298,0478	283,0248	255,0296		
30	Forsythoside G or isomer	C35H46O19	26,62		769,25551	607,2228	475,1826	329,1245	179,0334	161,0231		
31	Leucosceptoside B isomer	C36H48O19	26,84		783,27116	607,2235	589,2101	461,1677	193,0498	175,0389		
32[1]	Eriodictyol (3′,4′,5,7-Tetrahydroxyflavanone)	C15H12O6	26,89		287,05556	151,0023	135,0438	107,0123	83,0122			
33	Caffeoyl-hydroxybenzoylhexose	C22H22O11	27,02		461,10839	323,0772	283,0251	179,0338	161,0231	137,0231		
34	Tricin-7-O-glucoside	C23H24O12	27,09		491,11896	476,0960	327,0502	313,0354	298,0118	285,0412		
35	Abscisic acid	C15H20O4	27,22		263,12834	219,1383	204,1148	201,1276	152,0827	151,0749		
36	Luteolin-O-(acetylrhamnosyl)glucuronide isomer 1	C29H30O17	27,77		649,14048	285,0406	284,0332	199,0387				
37	Methoxy-tetrahydroxy(iso)flavone-O-(acetylrhamnosyl)glucuronide	C30H32O18	27,88		679,15104	315,0512	314,0392	300,0280	299,0196			
38	Leucosceptoside B	C36H48O19	28,04		783,27116	607,2247	589,2144	475,1845	329,1249	175,0389	[7]	
39	N1,N5,N10-Tricoumaroylspermidine isomer 1	C34H37N3O6	28,38		582,26042	462,2027	342,1456	316,1682	145,0282	119,0487		
40[1]	Naringenin (4',5,7-Trihydroxyflavanone)	C15H12O5	29,18		271,06065	177,0179	151,0024	119,0487	107,0124			
41	N1,N5,N10-Tricoumaroylspermidine isomer 2	C34H37N3O6	29,39		582,26042	462,2036	342,1456	316,1672	145,0282	119,0487		
42	Lubiminol or Canusesnol I	C15H26O2	29,40	239,20111		221,1906	203,1800	163,1482	135,1170	95,0859		
43	Luteolin-O-(acetylrhamnosyl)glucuronide isomer 2	C29H30O17	29,55		649,14048	285,0407	284,0328	199,0392	151,0022	133,0284		
44	Lubiminol or Canusesnol I	C15H26O2	30,25	239,20111		221,1898	203,1795	163,1482	135,1170	95,0860		
45[1]	Luteolin (3',4',5,7-Tetrahydroxyflavone)	C15H10O6	30,26		285,03991	217,0500	199,0389	175,0391	151,0024	133,0281		
46	N1,N5,N10-Tricoumaroylspermidine isomer 3	C34H37N3O6	30,26		582,26042	462,2042	342,1471	316,1661	145,0283	119,0487		
47	Apigenin-O-(acetylrhamnosyl)glucuronide	C29H30O16	30,96		633,14556	269,0456	268,0383	227,0340	225,0550	113,0228		
48	Chrysoeriol-O-(acetylrhamnosyl)glucuronide	C30H32O17	30,98		663,15612	299,0561	298,0491	284,0327	283,0260	256,0374		
49	N1,N5,N10-Tricoumaroylspermidine isomer 4	C34H37N3O6	31,14		582,26042	462,2029	342,1458	316,1668	145,0281	119,0487		
50	Apigenin-O-coumaroylhexoside	C30H26O12	31,39		577,13461	431,0989	269,0455	268,0376	145,0282			
51	Lubiminol or Canusesnol I isomer	C15H26O2	31,53	239,20111		221,1898	203,1796	163,1484	135,1174	95,0859		
52	Chrysoeriol-7-O-(3-Z-p-coumaroyl)glucose	C31H28O13	31,56		607,14517	461,1093	299,0562	284,0329	255,0298	145,0282		
53	Dimethoxy-trihydroxy(iso)flavone isomer 2	C17H14O7	31,76		329,06613	314,0433	313,0355	299,0197	285,0407	271,0249		
54[1]	Apigenin (4',5,7-Trihydroxyflavone)	C15H10O5	32,01		269,04500	227,0344	225,0550	151,0026	149,0229	117,0331		
55[1]	Chrysoeriol (3'-Methoxy-4',5,7-trihydroxyflavone)	C16H12O6	32,11		299,05556	284,0327	256,0374	227,0340	151,0021	107,0125		
56	Dimethoxy-trihydroxy(iso)flavone isomer 3	C17H14O7	32,36		329,06613	314,0434	313,0346	299,0199	271,0239	255,0288		

57	Chrysoeriol-7-O-(3-E-p-coumaroyl)glucose	C31H28O13	32,51		607,14517	461,1080	299,0559	284,0321	255,0290	145,0281	
58	Traumatic acid (2-Dodecenedioic acid)	C12H20O4	33,38		227,12834	209,1180	183,1381	165,1272	111,0802		
59	Traumatic acid isomer	C12H20O4	33,86		227,12834	209,1174	183,1381	165,1272	111,0801		
60	Acacetin (5,7-Dihydroxy-4'-methoxyflavone)	C16H12O5	35,57		283,06065	268,0378	240,0420	239,0326			
61	9-Hydroxyoctadecatrienoic acid	C18H30O3	40,76		293,21167	275,2018	231,2105	171,1014	121,1008	59,0122	
62	13-Hydroxyoctadecatrienoic acid	C18H30O3	40,93		293,21167	275,2019	235,1698	223,1331	195,1383	59,0122	
63	9-Hydroxyoctadecadienoic acid	C18H32O3	41,98		295,22732	277,2174	195,1384	171,1015	59,0122		
64	Unidentified terpenoid	C30H46O3	42,79	455,35253		437,3430	425,3395	409,3465	391,3373	189,1639	
65[1]	α-Linolenic acid	C18H30O2	45,18		277,21676	233,1895					
66[1]	Linoleic acid	C18H32O2	46,10		279,23241						
67	Linoleic acid isomer	C18H32O2	46,69		279,23241						
68[1]	Palmitic acid	C16H32O2	46,73		255,23241						
69[1]	Oleic acid	C18H34O2	47,04		281,24806						
70	Gondoic acid (cis-11-Eicosenoic acid) or isomer	C20H38O2	48,32		309,27936						
71	Pheophytin A	C55H74N4O5	65,06	871,57375		593,2758	533,2547	505,2189	460,2253	433,2413	

[1] Confirmed by standard

[2] Formate adduct

Table S6. Chemical composition of the methanol extract of *P. herba-venti*

No.	Name	Formula	Rt	[M + H]+	[M - H]-	Fragment 1	Fragment 2	Fragment 3	Fragment 4	Fragment 5	Literature
1	Quinic acid	C7H12O6	2,08		191,05557	173,0445	171,0284	127,0386	111,0436	85,0279	
2	Citric acid	C6H8O7	2,94		191,01918	173,0082	171,0287	111,0073	87,0072	85,0279	
3	Protocatechuic acid (3,4-Dihydroxybenzoic acid)	C7H6O4	8,99		153,01879	109,0280	108,0201	91,0171	81,0328		
4	Lamiide	C17H26O12	13,21		467.14008[2]	421,1349	403,1245	385,1140	259,0821	101,0230	[7]
5	Decaffeoylverbascoside	C20H30O12	14,49		461,16591	315,1094	297,0990	161,0441	135,0439	113,0229	
6	Unidentified iridoid	C17H26O12	15,85		467.14008[2]	403,1246	385,1138	331,1033	223,0606	179,0552	
7[1]	Chlorogenic acid (3-O-Caffeoylquinic acid)	C16H18O9	17,17	355,10291		163,0390	145,0285	135,0443	117,0339	89,0390	
8[1]	Caffeic acid	C9H8O4	17,52		179,03444	135,0438	107,0488				
9	Kynurenic acid	C10H7NO3	18,28	190,05042		162,0550	144,0446	116,0497	89,0391		
10	Naringenin-6,8-di-C-glucoside	C27H32O15	18,73		595,16630	475,1269	415,1005	385,0935	355,0821		
11	5-O-(4-Coumaroyl)quinic acid	C16H18O8	19,74		337,09235	191,0553	173,0445	163,0389	119,0487		
12	12-Hydroxyjasmonic acid or Tuberonic acid	C12H18O4	20,16		225,11269	181,1229	165,0908	163,1121	147,0803	59,0123	
13	12-Hydroxyjasmonic acid or Tuberonic acid	C12H18O4	20,37		225,11269	181,1228	165,0909	163,1121	147,0801	59,0123	
14	Caffeoylshikimic acid	C16H16O8	20,44		335,07670	179,0341	173,0446	161,0232	135,0439	111,0437	
15	5-O-Feruloylquinic acid	C17H20O9	20,57		367,10291	193,0500	191,0553	173,0444	134,0360		
16[1]	p-Coumaric acid	C9H8O3	20,69		163,03952	119,0488					
17	Vicenin-2 (Apigenin-6,8-di-C-glucoside)	C27H30O15	20,88	595,16630		577,1539	541,1342	457,1136	325,0707	295,0602	
18	5-O-(4-Coumaroyl)quinic acid cis isomer	C16H18O8	21,33		337,09235	191,0553	173,0442	163,0387	119,0487		
19	12-Hydroxyjasmonic acid sulfate or Tuberonic acid sulfate	C12H18O7S	22,03		305,06950	225,1126	96,9586	59,0123			
20	5-O-Feruloylquinic acid cis isomer	C17H20O9	22,15		367,10291	193,0497	191,0554	173,0442	134,0359		
21	Pentahydroxy(iso)flavone-O-rhamnosylglucuronide	C27H28O17	22,44		623,12483	301,0356	300,0263	255,0302	227,0341		
22	Pentahydroxy(iso)flavone-O-hexoside	C21H20O12	23,10		463,08765	301,0355	300,0277	283,0246	255,0299	228,0420	
23	Forsythoside B	C34H44O19	23,36		755,23986	593,2091	461,1685	447,1495	315,1094	161,0232	[7]
24	Luteolin-O-rhamnosylhexoside isomer 1	C27H30O15	23,67		593,15065	285,0407	284,0329	283,0259	133,0277		
25	Verbascoside (Acteoside)	C29H36O15	23,70		623,19760	461,1673	315,1088	179,0340	161,0232	133,0283	
26	Luteolin-O-rhamnosylglucuronide isomer 1	C27H28O16	23,99		607,12991	285,0406	284,0333	133,0278			
27	Luteolin-O-pentosylhexoside	C26H28O15	24,21		579,13500	285,0407	284,0324	151,0023	133,0285		

28	Forsythoside B isomer	C34H44O19	24,43		755,23986	593,2094	461,1670	315,1091	179,0338	161,0232	
29	Luteolin-O-rhamnosylhexoside isomer 2	C27H30O15	24,52		593,15065	285,0407	284,0329	151,0018	133,0278		
30	Hattushoside	C28H36O15	24,60		611,19760	431,1581	413,1470	299,1143	197,0448	153,0544	[7]
31[1]	Luteolin-7-O-glucoside (Cynaroside)	C21H20O11	24,61		447,09274	285,0407	284,0328	256,0393	151,0023	133,0283	
32	Luteolin-O-crotonylhexoside	C25H24O12	24,61		515,11896	447,0933	285,0405	284,0327			
33	Alyssonoside	C35H46O19	24,82		769,25551	593,2089	575,1967	461,1667	447,1514	175,0389	[7]
34	Methoxy-tetrahydroxy(iso)flavone-O-hexoside isomer 1	C22H22O12	25,00		477,10330	462,0814	315,0512	314,0433	300,0276	299,0199	
35	Isoverbascoside	C29H36O15	25,05		623,19760	461,1671	315,1086	179,0338	161,0232	135,0439	
36	Luteolin-O-rhamnosylhexoside isomer 3	C27H30O15	25,11		593,15065	447,0958	285,0407	284,0328	151,0025	133,0283	
37	Luteolin-O-glucuronide	C21H18O12	25,30		461,07201	285,0407	199,0400	175,0381	151,0025	133,0281	
38	Luteolin-O-rhamnosylglucuronide isomer 2	C27H28O16	25,33		607,12991	285,0406	284,0329	151,0021	133,0279		
39	Leucosceptoside A	C30H38O15	25,40		637,21325	461,1667	443,1572	315,1067	175,0389	160,0153	
40	Methoxy-tetrahydroxy(iso)flavone-O-hexoside isomer 2	C22H22O12	25,41		477,10330	462,0794	315,0513	313,0353	300,0276	299,0197	
41	Methoxy-tetrahydroxy(iso)flavone-O-glucuronide isomer 1	C22H20O13	25,68		491,08257	315,0512	300,0273				
42	Chrysoeriol-O-pentosylhexoside	C27H30O15	25,91		593,15065	299,0564	297,0388	285,0406	284,0326		
43	Alyssonoside isomer	C35H46O19	25,93		769,25551	593,2091	575,1959	461,1667	315,1090	175,0389	
44	Methoxy-tetrahydroxy(iso)flavone-O-glucuronide isomer 2	C22H20O13	26,16		491,08257	315,0518	300,0282				
45[1]	Cosmosiin (Apigenin-7-O-glucoside)	C21H20O10	26,20	433,11347		271,0602	153,0181	119,0492			
46	Dicaffeoylquinic acid	C25H24O12	26,29		515,11896	353,0877	335,0760	191,0553	179,0342	173,0445	
47	Chrysoeriol-7-O-glucoside	C22H22O11	26,47		461,10839	446,0858	299,0565	298,0484	283,0250	255,0298	
48	Forsythoside G or isomer	C35H46O19	26,63		769,25551	607,2264	475,1831	329,1249	179,0337	161,0232	
49	Leucosceptoside B isomer	C36H48O19	26,86		783,27116	607,2263	589,2136	461,1674	193,0500	175,0390	
50[1]	Apigenin-7-O-glucuronide	C21H18O11	26,87		445,07709	269,0457	175,0246	113,0229			
51[1]	Eriodictyol (3′,4′,5,7-Tetrahydroxyflavanone)	C15H12O6	26,90		287,05556	151,0024	135,0439	107,0124	83,0123		
52	Caffeoyl-hydroxybenzoylhexose	C22H22O11	27,03		461,10839	323,0772	283,0254	179,0340	161,0231	137,0231	
53	Chrysoeriol-O-glucuronide isomer 1	C22H20O12	27,09		475,08766	299,0566	284,0326				
54	Tricin-7-O-glucoside	C23H24O12	27,11		491,11896	476,0962	327,0514	313,0356	298,0121	285,0409	
55	Abscisic acid	C15H20O4	27,23		263,12834	219,1382	204,1142	201,1269	152,0827	151,0751	
56	Chrysoeriol-O-glucuronide isomer 2	C22H20O12	27,45		475,08766	299,0560	284,0326				
57	Chrysoeriol-O-glucuronide isomer 3	C22H20O12	27,70		475,08766	299,0564	284,0328				
58	Luteolin-O-(acetylrhamnosyl)glucuronide isomer 1	C29H30O17	27,81		649,14048	285,0406	284,0324	199,0390			
59	Methoxy-tetrahydroxy(iso)flavone-O-(acetylrhamnosyl)glucuronide	C30H32O18	27,92		679,15104	315,0512	314,0444	300,0276	299,0199		
60	Leucosceptoside B	C36H48O19	28,05		783,27116	607,2244	589,2128	475,1810	329,1230	175,0389	[7]
61	N1,N5,N10-Tricoumaroylspermidine isomer 1	C34H37N3O6	28,41		582,26042	462,2036	342,1467	316,1675	145,0282	119,0487	
62[1]	Naringenin (4',5,7-Trihydroxyflavanone)	C15H12O5	29,20		271,06065	177,0194	151,0022	119,0489	107,0124		

63	N1,N5,N10-Tricoumaroylspermidine isomer 2	C34H37N3O6	29,39		582,26042	462,2039	342,1463	316,1673	145,0282	119,0488	
64	Lubiminol or Canusesnol I	C15H26O2	29,40	239,20111		221,1902	203,1795	163,1482	135,1170	95,0860	
65	Luteolin-O-(acetylrhamnosyl)glucuronide isomer 2	C29H30O17	29,57		649,14048	285,0406	284,0327	199,0393	151,0022	133,0279	
66	Lubiminol or Canusesnol I	C15H26O2	30,25	239,20111		221,1898	203,1794	163,1481	135,1169	95,0860	
67[1]	Luteolin (3',4',5,7-Tetrahydroxyflavone)	C15H10O6	30,26		285,03991	217,0496	199,0396	175,0392	151,0022	133,0281	
68	N1,N5,N10-Tricoumaroylspermidine isomer 3	C34H37N3O6	30,26		582,26042	462,2038	342,1450	316,1659	145,0281	119,0488	
69	Apigenin-O-(acetylrhamnosyl)glucuronide	C29H30O16	30,96		633,14556	269,0456	268,0374	227,0341	225,0550	113,0229	
70	Chrysoeriol-O-(acetylrhamnosyl)glucuronide	C30H32O17	31,02		663,15612	299,0562	298,0472	284,0327	283,0250	256,0371	
71	N1,N5,N10-Tricoumaroylspermidine isomer 4	C34H37N3O6	31,15		582,26042	462,2043	342,1454	316,1652	145,0280	119,0487	
72	Apigenin-O-coumaroylhexoside	C30H26O12	31,40		577,13461	431,0986	269,0456	268,0377	145,0282		
73	Chrysoeriol-7-O-(3-Z-p-coumaroyl)glucose	C31H28O13	31,56		607,14517	461,1087	299,0562	284,0328	255,0294	145,0282	
74	Dimethoxy-trihydroxy(iso)flavone isomer 2	C17H14O7	31,76		329,06613	314,0442	313,0333	299,0197	285,0392	271,0248	
75[1]	Apigenin (4',5,7-Trihydroxyflavone)	C15H10O5	32,01		269,04500	227,0346	225,0551	151,0024	149,0231	117,0330	
76[1]	Chrysoeriol (3'-Methoxy-4',5,7-trihydroxyflavone)	C16H12O6	32,24		299,05556	284,0327	256,0376	227,0347	151,0019	107,0122	
77	Dimethoxy-trihydroxy(iso)flavone isomer 3	C17H14O7	32,31		329,06613	314,0436	313,0354	299,0198	271,0250	255,0289	
78	Chrysoeriol-7-O-(3-E-p-coumaroyl)glucose	C31H28O13	32,57		607,14517	461,1088	299,0563	284,0327	255,0299	145,0282	
79	Traumatic acid (2-Dodecenedioic acid)	C12H20O4	33,39		227,12834	209,1172	183,1381	165,1272	111,0803		
80	Traumatic acid isomer	C12H20O4	33,87		227,12834	209,1173	183,1381	165,1272	111,0803		
81	Acacetin (5,7-Dihydroxy-4'-methoxyflavone)	C16H12O5	35,58		283,06065	268,0377	240,0423	239,0349			
82	9-Hydroxyoctadecatrienoic acid	C18H30O3	40,77		293,21167	275,2021	231,2116	171,1016	121,1008	59,0123	
83	13-Hydroxyoctadecatrienoic acid	C18H30O3	40,94		293,21167	275,2021	235,1703	223,1335	195,1384	59,0122	
84	9-Hydroxyoctadecadienoic acid	C18H32O3	41,99		295,22732	277,2174	195,1382	171,1013	59,0121		
85[1]	α-Linolenic acid	C18H30O2	45,19		277,21676	233,1899					
86[1]	Linoleic acid	C18H32O2	46,09		279,23241						
87	Linoleic acid isomer	C18H32O2	46,66		279,23241						
88	Pheophytin A	C55H74N4O5	64,93	871,57375		593,2760	533,2549	505,2205	460,2255	433,2403	

[1] Confirmed by standard

[2] Formate adduct

Table S7. Chemical composition of the methanol/water extract of *P. herba-venti*

No.	Name	Formula	Rt	[M + H]$^+$	[M - H]$^-$	Fragment 1	Fragment 2	Fragment 3	Fragment 4	Fragment 5	Literature
1	Quinic acid	C7H12O6	2,05		191,05557	173,0443	171,0290	127,0388	111,0437	85,0279	
2	Citric acid	C6H8O7	2,98		191,01918	173,0085	171,0287	111,0073	87,0072	85,0279	
3	Protocatechuic acid (3,4-Dihydroxybenzoic acid)	C7H6O4	8,97		153,01879	109,0280	108,0202	91,0173	81,0331		
4	Decaffeoylverbascoside	C20H30O12	14,50		461,16591	315,1091	297,0989	161,0442	135,0438	113,0229	
5	Unidentified iridoid	C17H26O12	15,87		467.14008[2]	403,1256	385,1146	331,1050	223,0604	179,0552	
6[1]	Chlorogenic acid (3-O-Caffeoylquinic acid)	C16H18O9	17,19	355,10291		163,0390	145,0286	135,0443	117,0337	89,0390	
7[1]	Caffeic acid	C9H8O4	17,55		179,03444	135,0438	107,0489				
8	Kynurenic acid	C10H7NO3	18,44	190,05042		162,0551	144,0439	116,0499	89,0389		
9	Naringenin-6,8-di-C-glucoside	C27H32O15	18,73		595,16630	475,1239	415,1024	385,0931	355,0821		
10	5-O-(4-Coumaroyl)quinic acid	C16H18O8	19,78		337,09235	191,0553	173,0444	163,0388	119,0488		
11	12-Hydroxyjasmonic acid or Tuberonic acid	C12H18O4	20,17		225,11269	181,1221	165,0910	163,1120	147,0802	59,0123	
12	12-Hydroxyjasmonic acid or Tuberonic acid	C12H18O4	20,39		225,11269	181,1245	165,0908	163,1112	147,0803	59,0123	
13	Caffeoylshikimic acid	C16H16O8	20,46		335,07670	179,0340	173,0451	161,0232	135,0439	111,0437	
14[1]	Riboflavin	C17H20N4O6	20,50	377,14611		359,0715	243,0879	172,0867	99,0448	69,0341	
15	5-O-Feruloylquinic acid	C17H20O9	20,61		367,10291	193,0499	191,0553	173,0444	134,0360		
16[1]	p-Coumaric acid	C9H8O3	20,72		163,03952	119,0488					
17	Vicenin-2 (Apigenin-6,8-di-C-glucoside)	C27H30O15	20,90	595,16630		577,1519	541,1351	457,1131	325,0708	295,0605	
18	5-O-(4-Coumaroyl)quinic acid cis isomer	C16H18O8	21,36		337,09235	191,0553	173,0444	163,0391	119,0491		
19	5-O-Feruloylquinic acid cis isomer	C17H20O9	22,17		367,10291	193,0497	191,0554	173,0448	134,0361		
20	12-Hydroxyjasmonic acid sulfate or Tuberonic acid sulfate	C12H18O7S	22,29		305,06950	225,1122	96,9585	59,0123			
21	Pentahydroxy(iso)flavone-O-rhamnosylglucuronide	C27H28O17	22,51		623,12483	301,0354	300,0281	255,0300	227,0343		
22	Pentahydroxy(iso)flavone-O-hexoside	C21H20O12	23,13		463,08765	301,0355	300,0277	283,0232	255,0288	228,0425	
23	Forsythoside B	C34H44O19	23,37		755,23986	593,2088	461,1685	447,1505	315,1092	161,0231	[7]
24	Verbascoside (Acteoside)	C29H36O15	23,71		623,19760	461,1668	315,1090	179,0341	161,0232	133,0282	
25	Luteolin-O-rhamnosylhexoside isomer 1	C27H30O15	23,78		593,15065	285,0407	284,0326	283,0256	133,0278		

26	Luteolin-O-rhamnosylglucuronide isomer 1	C27H28O16	24,07		607,12991	285,0406	284,0318	133,0268			
27	Luteolin-O-pentosylhexoside	C26H28O15	24,23		579,13500	285,0407	284,0325	151,0023	133,0278		
28	Forsythoside B isomer	C34H44O19	24,43		755,23986	593,2089	461,1680	315,1081	179,0338	161,0232	
29	Luteolin-O-rhamnosylhexoside isomer 2	C27H30O15	24,54		593,15065	285,0407	284,0328	151,0026	133,0278		
30	Hattushoside	C28H36O15	24,62		611,19760	431,1573	413,1437	299,1142	197,0448	153,0545	[7]
31[1]	Luteolin-7-O-glucoside (Cynaroside)	C21H20O11	24,62		447,09274	285,0407	284,0328	256,0378	151,0026	133,0282	
32	Luteolin-O-crotonylhexoside	C25H24O12	24,64		515,11896	447,0933	285,0407	284,0330			
33	Alyssonoside	C35H46O19	24,83		769,25551	593,2090	575,1981	461,1663	447,1515	175,0390	[7]
34	Methoxy-tetrahydroxy(iso)flavone-O-hexoside isomer 1	C22H22O12	25,02		477,10330	462,0797	315,0512	314,0433	300,0276	299,0198	
35	Isoverbascoside	C29H36O15	25,06		623,19760	461,1670	315,1097	179,0342	161,0232	135,0439	
36	Luteolin-O-rhamnosylhexoside isomer 3	C27H30O15	25,12		593,15065	447,0929	285,0407	284,0328	151,0019	133,0281	
37	Luteolin-O-rhamnosylglucuronide isomer 2	C27H28O16	25,38		607,12991	285,0406	284,0328	151,0022	133,0278		
38	Leucosceptoside A	C30H38O15	25,42		637,21325	461,1667	443,1577	315,1090	175,0390	160,0154	
39	Methoxy-tetrahydroxy(iso)flavone-O-hexoside isomer 2	C22H22O12	25,43		477,10330	462,0845	315,0514	313,0357	300,0277	299,0199	
40	Methoxy-tetrahydroxy(iso)flavone-O-glucuronide isomer 1	C22H20O13	25,76		491,08257	315,0515	300,0278				
41	Chrysoeriol-O-pentosylhexoside	C27H30O15	25,93		593,15065	299,0563	297,0389	285,0411	284,0328		
42	Alyssonoside isomer	C35H46O19	25,95		769,25551	593,2089	575,1940	461,1671	315,1098	175,0389	
43[1]	Cosmosiin (Apigenin-7-O-glucoside)	C21H20O10	26,20	433,11347		271,0602	153,0186	119,0495			
44	Methoxy-tetrahydroxy(iso)flavone-O-glucuronide isomer 2	C22H20O13	26,25		491,08257	315,0509	300,0277				
45	Dicaffeoylquinic acid	C25H24O12	26,31		515,11896	353,0866	335,0762	191,0554	179,0343	173,0443	
46	Chrysoeriol-7-O-glucoside	C22H22O11	26,48		461,10839	446,0858	299,0562	298,0485	283,0251	255,0297	
47	Forsythoside G or isomer	C35H46O19	26,65		769,25551	607,2266	475,1817	329,1249	179,0337	161,0232	
48	Leucosceptoside B isomer	C36H48O19	26,86		783,27116	607,2236	589,2170	461,1650	193,0498	175,0390	
49[1]	Eriodictyol (3′,4′,5,7-Tetrahydroxyflavanone)	C15H12O6	26,92		287,05556	151,0023	135,0439	107,0122	83,0119		
50[1]	Apigenin-7-O-glucuronide	C21H18O11	26,97		445,07709	269,0457	175,0231	113,0229			
51	Caffeoyl-hydroxybenzoylhexose	C22H22O11	27,05		461,10839	323,0773	283,0247	179,0340	161,0232	137,0231	
52	Tricin-7-O-glucoside	C23H24O12	27,12		491,11896	476,0967	327,0512	313,0357	298,0122	285,0406	
53	Chrysoeriol-O-glucuronide isomer 1	C22H20O12	27,17		475,08766	299,0566	284,0324				
54	Abscisic acid	C15H20O4	27,24		263,12834	219,1385	204,1150	201,1286	152,0829	151,0752	
55	Chrysoeriol-O-glucuronide isomer 2	C22H20O12	27,52		475,08766	299,0563	284,0328				
56	Chrysoeriol-O-glucuronide isomer 3	C22H20O12	27,79		475,08766	299,0563	284,0329				

57	Luteolin-O-(acetylrhamnosyl)glucuronide isomer 1	C29H30O17	27,89		649,14048	285,0406	284,0330	199,0388				
58	Methoxy-tetrahydroxy(iso)flavone-O-(acetylrhamnosyl)glucuronide	C30H32O18	28,00		679,15104	315,0513	314,0438	300,0277	299,0204			
59	Leucosceptoside B	C36H48O19	28,06		783,27116	607,2253	589,2155	475,1829	329,1247	175,0390		[7]
60	N1,N5,N10-Tricoumaroylspermidine isomer 1	C34H37N3O6	28,40		582,26042	462,2034	342,1455	316,1692	145,0283	119,0488		
61[1]	Naringenin (4',5,7-Trihydroxyflavanone)	C15H12O5	29,20		271,06065	177,0177	151,0023	119,0487	107,0124			
62	N1,N5,N10-Tricoumaroylspermidine isomer 2	C34H37N3O6	29,39		582,26042	462,2037	342,1458	316,1660	145,0283	119,0487		
63	Lubiminol or Canusesnol I	C15H26O2	29,40	239,20111		221,1900	203,1795	163,1479	135,1171	95,0861		
64	Luteolin-O-(acetylrhamnosyl)glucuronide isomer 2	C29H30O17	29,63		649,14048	285,0405	284,0328	199,0392	151,0023	133,0281		
65	Dimethoxy-trihydroxy(iso)flavone isomer 1	C17H14O7	30,20		329,06613	314,0436	313,0338	299,0195	271,0247			
66	Lubiminol or Canusesnol I	C15H26O2	30,26	239,20111		221,1901	203,1797	163,1485	135,1171	95,0860		
67[1]	Luteolin (3',4',5,7-Tetrahydroxyflavone)	C15H10O6	30,27		285,03991	217,0500	199,0393	175,0389	151,0024	133,0281		
68	N1,N5,N10-Tricoumaroylspermidine isomer 3	C34H37N3O6	30,28		582,26042	462,2039	342,1456	316,1664	145,0283	119,0487		
69	Apigenin-O-(acetylrhamnosyl)glucuronide	C29H30O16	31,04		633,14556	269,0456	268,0375	227,0347	225,0556	113,0229		
70	Chrysoeriol-O-(acetylrhamnosyl)glucuronide	C30H32O17	31,06		663,15612	299,0562	298,0485	284,0328	283,0262	256,0372		
71	N1,N5,N10-Tricoumaroylspermidine isomer 4	C34H37N3O6	31,16		582,26042	462,2025	342,1460	316,1654	145,0280	119,0489		
72	Apigenin-O-coumaroylhexoside	C30H26O12	31,41		577,13461	431,0986	269,0456	268,0378	145,0282			
73	Chrysoeriol-7-O-(3-Z-p-coumaroyl)glucose	C31H28O13	31,60		607,14517	461,1093	299,0562	284,0327	255,0296	145,0282		
74	Dimethoxy-trihydroxy(iso)flavone isomer 2	C17H14O7	31,77		329,06613	314,0438	313,0352	299,0195	285,0397	271,0244		
75[1]	Apigenin (4',5,7-Trihydroxyflavone)	C15H10O5	32,01		269,04500	227,0341	225,0550	151,0024	149,0230	117,0330		
76[1]	Chrysoeriol (3'-Methoxy-4',5,7-trihydroxyflavone)	C16H12O6	32,26		299,05556	284,0327	256,0375	227,0338	151,0019	107,0122		
77	Dimethoxy-trihydroxy(iso)flavone isomer 3	C17H14O7	32,31		329,06613	314,0432	313,0365	299,0197	271,0249			
78	Chrysoeriol-7-O-(3-E-p-coumaroyl)glucose	C31H28O13	32,57		607,14517	461,1092	299,0562	284,0327	255,0294	145,0282		
79	Traumatic acid (2-Dodecenedioic acid)	C12H20O4	33,40		227,12834	209,1178	183,1381	165,1273	111,0803			
80	Traumatic acid isomer	C12H20O4	33,87		227,12834	209,1173	183,1381	165,1273	111,0800			
81	Acacetin (5,7-Dihydroxy-4'-methoxyflavone)	C16H12O5	35,58		283,06065	268,0378	240,0427	239,0344				
82	9-Hydroxyoctadecatrienoic acid	C18H30O3	40,78		293,21167	275,2021	231,2116	171,1016	121,1008	59,0122		
83	13-Hydroxyoctadecatrienoic acid	C18H30O3	40,95		293,21167	275,2022	235,1696	223,1334	195,1383	59,0123		
84	9-Hydroxyoctadecadienoic acid	C18H32O3	41,99		295,22732	277,2174	195,1381	171,1014	59,0122			
85[1]	α-Linolenic acid	C18H30O2	45,20		277,21676	233,1906						
86[1]	Linoleic acid	C18H32O2	46,11		279,23241							
87	Linoleic acid isomer	C18H32O2	46,65		279,23241							

[1] Confirmed by standard [2] Formate adduct

Table S8. Chemical composition of the water extract of *P. herba-venti*

No.	Name	Formula	Rt	[M + H]$^{+}$	[M - H]$^{-}$	Fragment 1	Fragment 2	Fragment 3	Fragment 4	Fragment 5	Literature
1	Quinic acid	C7H12O6	2,05		191,05557	173,0442	171,0286	127,0387	111,0435	85,0279	
2	Citric acid	C6H8O7	2,96		191,01918	173,0083	171,0287	111,0072	87,0071	85,0279	
3	Protocatechuic acid (3,4-Dihydroxybenzoic acid)	C7H6O4	9,01		153,01879	109,0280	108,0202	91,0173	81,0331		
4	Lamiide	C17H26O12	13,24		467.14008[2]	421,1336	403,1249	385,1143	259,0833	101,0230	[7]
5[1]	Neochlorogenic acid (5-O-Caffeoylquinic acid)	C16H18O9	13,55	355,10291		163,0389	145,0285	135,0442	117,0337	89,0389	
6	Unidentified iridoid	C17H26O12	15,88		467.14008[2]	403,1242	385,1139	331,1032	223,0606	179,0552	
7[1]	Chlorogenic acid (3-O-Caffeoylquinic acid)	C16H18O9	17,24	355,10291		163,0389	145,0284	135,0441	117,0337	89,0389	
8[1]	Caffeic acid	C9H8O4	17,54		179,03444	135,0438	107,0488				
9[1]	Chryptochlorogenic acid (4-O-Caffeoylquinic acid)	C16H18O9	18,08	355,10291		163,0389	145,0284	135,0442	117,0339	89,0389	
10	Kynurenic acid	C10H7NO3	18,55	190,05042		162,0550	144,0444	116,0496	89,0388		
11	Naringenin-6,8-di-C-glucoside	C27H32O15	18,75		595,16630	475,1235	415,1039	385,0930	355,0825		
12	5-O-(4-Coumaroyl)quinic acid	C16H18O8	19,79		337,09235	191,0552	173,0443	163,0388	119,0487		
13	12-Hydroxyjasmonic acid or Tuberonic acid	C12H18O4	20,18		225,11269	181,1230	165,0910	163,1109	147,0802	59,0123	
14	12-Hydroxyjasmonic acid or Tuberonic acid	C12H18O4	20,40		225,11269	181,1220	165,0913	163,1129	147,0806	59,0123	
15	Caffeoylshikimic acid	C16H16O8	20,46		335,07670	179,0340	173,0439	161,0232	135,0438	111,0436	
16	Riboflavin	C17H20N4O6	20,51	377,14611		359,0731	243,0876	172,0871	99,0447	69,0341	
17	5-O-Feruloylquinic acid	C17H20O9	20,63		367,10291	193,0497	191,0552	173,0443	134,0360		
18[1]	p-Coumaric acid	C9H8O3	20,71		163,03952	119,0487					
19	Vicenin-2 (Apigenin-6,8-di-C-glucoside)	C27H30O15	20,90	595,16630		577,1542	541,1337	457,1130	325,0707	295,0601	
20	5-O-(4-Coumaroyl)quinic acid cis isomer	C16H18O8	21,38		337,09235	191,0553	173,0447	163,0387	119,0486		
21	12-Hydroxyjasmonic acid sulfate or Tuberonic acid sulfate	C12H18O7S	22,15		305,06950	225,1125	96,9586	59,0122			
22	5-O-Feruloylquinic acid cis isomer	C17H20O9	22,18		367,10291	193,0498	191,0553	173,0444	134,0363		
23	Pentahydroxy(iso)flavone-O-rhamnosylglucuronide	C27H28O17	22,55		623,12483	301,0354	300,0281	255,0301	227,0329		
24	Pentahydroxy(iso)flavone-O-hexoside	C21H20O12	23,13		463,08765	301,0357	300,0276	283,0247	255,0302	228,0422	

25	Luteolin-O-rhamnosylhexoside isomer 1	C27H30O15	23,69		593,15065	285,0407	284,0328	283,0257	133,0278		
26	Luteolin-O-rhamnosylglucuronide isomer 1	C27H28O16	24,11		607,12991	285,0405	284,0339	133,0278			
27	Luteolin-O-pentosylhexoside	C26H28O15	24,25		579,13500	285,0406	284,0326	151,0022	133,0279		
28	Luteolin-O-rhamnosylhexoside isomer 2	C27H30O15	24,54		593,15065	285,0406	284,0335	151,0017	133,0275		
29	Hattushoside	C28H36O15	24,60		611,19760	431,1577	413,1448	299,1143	197,0447	153,0544	[7]
30[1]	Luteolin-7-O-glucoside (Cynaroside)	C21H20O11	24,61		447,09274	285,0406	284,0327	256,0374	151,0025	133,0282	
31	Luteolin-O-crotonylhexoside	C25H24O12	24,63		515,11896	447,0930	285,0406	284,0328			
32	Methoxy-tetrahydroxy(iso)flavone-O-hexoside isomer 1	C22H22O12	25,02		477,10330	462,0840	315,0514	314,0430	300,0279	299,0189	
33	Luteolin-O-rhamnosylhexoside isomer 3	C27H30O15	25,12		593,15065	447,0945	285,0405	284,0327	151,0023	133,0281	
34	Luteolin-O-glucuronide	C21H18O12	25,29		461,07201	285,0406	199,0389	175,0390	151,0022	133,0284	
35	Luteolin-O-rhamnosylglucuronide isomer 2	C27H28O16	25,40		607,12991	285,0405	284,0328	151,0025	133,0280		
36	Methoxy-tetrahydroxy(iso)flavone-O-hexoside isomer 2	C22H22O12	25,41		477,10330	462,0785	315,0511	313,0350	300,0275	299,0199	
37	Methoxy-tetrahydroxy(iso)flavone-O-glucuronide isomer 1	C22H20O13	25,81		491,08257	315,0515	300,0283				
38	Chrysoeriol-O-pentosylhexoside	C27H30O15	25,93		593,15065	299,0561	297,0389	285,0411	284,0326		
39[1]	Cosmosiin (Apigenin-7-O-glucoside)	C21H20O10	26,20	433,11347		271,0600	153,0180	119,0490			
40	Methoxy-tetrahydroxy(iso)flavone-O-glucuronide isomer 2	C22H20O13	26,29		491,08257	315,0512	300,0278				
41	Chrysoeriol-7-O-glucoside	C22H22O11	26,48		461,10839	446,0853	299,0560	298,0480	283,0249	255,0297	
42	Forsythoside G or isomer	C35H46O19	26,65		769,25551	607,2238	475,1830	329,1245	179,0337	161,0231	
43	Leucosceptoside B isomer	C36H48O19	26,87		783,27116	607,2260	589,2122	461,1671	193,0499	175,0389	
44[1]	Apigenin-7-O-glucuronide	C21H18O11	26,99		445,07709	269,0455	175,0229	113,0228			
45	Caffeoyl-hydroxybenzoylhexose	C22H22O11	27,06		461,10839	323,0769	283,0244	179,0340	161,0231	137,0230	
46	Tricin-7-O-glucoside	C23H24O12	27,12		491,11896	476,0958	327,0509	313,0355	298,0117	285,0409	
47	Chrysoeriol-O-glucuronide isomer 1	C22H20O12	27,20		475,08766	299,0558	284,0330				
48	Abscisic acid	C15H20O4	27,25		263,12834	219,1386	204,1148	201,1284	152,0832	151,0749	
49	Chrysoeriol-O-glucuronide isomer 2	C22H20O12	27,57		475,08766	299,0561	284,0328				
50	Chrysoeriol-O-glucuronide isomer 3	C22H20O12	27,84		475,08766	299,0562	284,0326				
51	Luteolin-O-(acetylrhamnosyl)glucuronide isomer 1	C29H30O17	27,97		649,14048	285,0405	284,0322	199,0390			
52	Methoxy-tetrahydroxy(iso)flavone-O-(acetylrhamnosyl)glucuronide	C30H32O18	28,05		679,15104	315,0511	314,0428	300,0275	299,0201		
53	Leucosceptoside B	C36H48O19	28,06		783,27116	607,2247	589,2149	475,1814	329,1247	175,0389	[7]
54	N1,N5,N10-Tricoumaroylspermidine isomer 1	C34H37N3O6	28,41		582,26042	462,2039	342,1458	316,1634	145,0280	119,0487	
55[1]	Naringenin (4',5,7-Trihydroxyflavanone)	C15H12O5	29,22		271,06065	177,0192	151,0020	119,0485	107,0123		

56	Lubiminol or Canusesnol I	C15H26O2	29,40	239,20111		221,1905	203,1800	163,1483	135,1169	95,0860		
57	N1,N5,N10-Tricoumaroylspermidine isomer 2	C34H37N3O6	29,43		582,26042	462,2028	342,1446	316,1670	145,0279	119,0488		
58	Luteolin-O-(acetylrhamnosyl)glucuronide isomer 2	C29H30O17	29,66		649,14048	285,0405	284,0325	199,0396	151,0024	133,0279		
59	Dimethoxy-trihydroxy(iso)flavone isomer 1	C17H14O7	30,21		329,06613	314,0434	313,0338	299,0197	271,0246			
60	Lubiminol or Canusesnol I	C15H26O2	30,27	239,20111		221,1899	203,1797	163,1481	135,1170	95,0860		
61[1]	Luteolin (3',4',5,7-Tetrahydroxyflavone)	C15H10O6	30,28		285,03991	217,0498	199,0390	175,0389	151,0023	133,0281		
62	Apigenin-O-(acetylrhamnosyl)glucuronide	C29H30O16	31,06		633,14556	269,0455	268,0374	227,0346	225,0553	113,0229		
63	Chrysoeriol-O-(acetylrhamnosyl)glucuronide	C30H32O17	31,08		663,15612	299,0561	298,0477	284,0327	283,0245	256,0377		
64	Apigenin-O-coumaroylhexoside	C30H26O12	31,42		577,13461	431,0985	269,0452	268,0386	145,0282			
65	Chrysoeriol-7-O-(3-Z-p-coumaroyl)glucose	C31H28O13	31,60		607,14517	461,1085	299,0557	284,0329	255,0302	145,0282		
66	Dimethoxy-trihydroxy(iso)flavone isomer 2	C17H14O7	31,76		329,06613	314,0427	313,0349	299,0199	285,0397	271,0248		
67[1]	Apigenin (4',5,7-Trihydroxyflavone)	C15H10O5	32,03		269,04500	227,0347	225,0551	151,0022	149,0232	117,0330		
68[1]	Chrysoeriol (3'-Methoxy-4',5,7-trihydroxyflavone)	C16H12O6	32,26		299,05556	284,0327	256,0375	227,0334	151,0020	107,0123		
69	Dimethoxy-trihydroxy(iso)flavone isomer 3	C17H14O7	32,32		329,06613	314,0433	313,0333	299,0199	271,0250	255,0292		
70	Chrysoeriol-7-O-(3-E-p-coumaroyl)glucose	C31H28O13	32,56		607,14517	461,1085	299,0563	284,0322	255,0298	145,0280		
71	Traumatic acid (2-Dodecenedioic acid)	C12H20O4	33,42		227,12834	209,1173	183,1381	165,1272	111,0803			
72	Traumatic acid isomer	C12H20O4	33,88		227,12834	209,1172	183,1381	165,1272	111,0797			
73	Acacetin (5,7-Dihydroxy-4'-methoxyflavone)	C16H12O5	35,58		283,06065	268,0380	240,0427	239,0345				
74	9-Hydroxyoctadecatrienoic acid	C18H30O3	40,78		293,21167	275,2020	231,2109	171,1014	121,1009	59,0122		
75	13-Hydroxyoctadecatrienoic acid	C18H30O3	40,95		293,21167	275,2017	235,1695	223,1332	195,1384	59,0123		
76	9-Hydroxyoctadecadienoic acid	C18H32O3	41,99		295,22732	277,2175	195,1380	171,1016	59,0122			

[1] Confirmed by standard

[2] Formate adduct

Table S9. Chemical composition of the ethyl acetate extract of *P. kurdica*

No.	Name	Formula	Rt	$[M + H]^+$	$[M - H]^-$	Fragment 1	Fragment 2	Fragment 3	Fragment 4	Fragment 5	Literature
1	Quinic acid	C7H12O6	2,10		191,05557	173,0439	171,0285	127,0388	111,0436	85,0279	
2[1]	Chlorogenic acid (3-O-Caffeoylquinic acid)	C16H18O9	17,32	355,10291		163,0389	145,0283	135,0441	117,0341	89,0389	[8]
3[1]	Caffeic acid	C9H8O4	17,49		179,03444	135,0438	107,0485				
4[1]	Vanillin (4-Hydroxy-3-methoxybenzaldehyde)	C8H8O3	18,27	153,05517		125,0598	111,0443	110,0365	93,0339	65,0392	
5	Syringaldehyde (3,5-Dimethoxy-4-hydroxybenzaldehyde)	C9H10O4	19,74	183,06574		155,0699	140,0466	123,0442	105,0338	95,0495	
6	Caffeoylshikimic acid	C16H16O8	20,46		335,07670	179,0339	173,0421	161,0230	135,0438	111,0441	
7[1]	p-Coumaric acid	C9H8O3	20,71		163,03952	119,0487					
8[1]	Ferulic acid	C10H10O4	21,86		193,05009	178,0263	149,0598	137,0234	134,0358		
9	Eriodictyol-O-hexoside	C21H22O11	22,23		449,10839	287,0563	151,0025	135,0439	107,0125		
10	Forsythoside B	C34H44O19	23,40		755,23986	593,2083	461,1676	447,1521	315,1087	161,0231	[9]
11	Verbascoside (Acteoside)	C29H36O15	23,75		623,19760	461,1669	315,1096	179,0338	161,0231	133,0282	[9]
12	Prunin (Naringenin-7-O-glucoside)	C21H22O10	24,28		433,11347	271,0613	177,0182	151,0027	119,0488	107,0122	
13	Samioside	C34H44O19	24,40		755,23986	593,2083	461,1676	315,1087	179,0340	161,0231	
14	Hattushoside	C28H36O15	24,63		611,19760	431,1577	413,1460	299,1135	197,0448	153,0544	
15[1]	Luteolin-7-O-glucoside (Cynaroside)	C21H20O11	24,65		447,09274	285,0404	284,0327	256,0381	151,0023	133,0282	[8]
16	Alyssonoside	C35H46O19	24,85		769,25551	593,2097	575,1973	461,1677	447,1497	175,0389	[9]
17	Forsythoside B isomer	C34H44O19	25,07		755,23986	593,2071	461,1655	447,1476	315,1091	161,0231	
18	Isoverbascoside	C29H36O15	25,09		623,19760	461,1675	315,1089	179,0342	161,0231	135,0439	
19	Leucosceptoside A	C30H38O15	25,44		637,21325	461,1663	443,1544	315,1077	175,0390	160,0154	[9]
20	Caffeoyl-vanilloylglucose	C23H24O12	25,64		491,11896	323,0771	179,0335	167,0331	161,0233	152,0105	
21	Chrysoeriol-O-pentosylhexoside	C27H30O15	25,96		593,15065	299,0561	297,0385	285,0401	284,0324		
22	Alyssonoside isomer	C35H46O19	26,14		769,25551	593,2069	575,1997	461,1653	315,1105	175,0390	

23	Rosmarinic acid (Labiatenic acid)	C18H16O8	26,35		359,07670	197,0446	179,0339	161,0230	135,0438	72,9915	
24	Chrysoeriol-7-O-glucoside	C22H22O11	26,51		461,10839	446,0855	299,0560	298,0484	283,0248	255,0296	
25	Leucosceptoside B isomer	C36H48O19	26,89		783,27116	607,2246	589,2130	461,1670	193,0499	175,0390	
26[1]	Eriodictyol (3′,4′,5,7-Tetrahydroxyflavanone)	C15H12O6	26,95		287,05556	269,0504	151,0025	135,0442	107,0125	83,0122	
27	Caffeoyl-hydroxybenzoylhexose	C22H22O11	27,07		461,10839	323,0768	283,0249	179,0339	161,0230	137,0231	
28	Chrysoeriol-O-glucuronide	C22H20O12	27,26		475,08766	299,0558	284,0313	256,0366			
29	Abscisic acid	C15H20O4	27,28		263,12834	219,1388	204,1144	201,1261	152,0829	151,0747	
30	Martynoside	C31H40O15	27,67		651,22890	475,1819	329,1241	193,0502	175,0389	160,0153	
31	Leucosceptoside B	C36H48O19	28,05		783,27116	607,2226	589,2129	475,1848	329,1265	175,0389	[9]
32	N1,N5,N10-Tricoumaroylspermidine isomer 1	C34H37N3O6	28,43		582,26042	462,2017	342,1441	316,1650	145,0285	119,0487	
33[1]	Naringenin (4',5,7-Trihydroxyflavanone)	C15H12O5	29,23		271,06065	227,0702	177,0183	151,0023	119,0487	107,0123	
34	N1,N5,N10-Tricoumaroylspermidine isomer 2	C34H37N3O6	29,41		582,26042	462,2068	342,1452	316,1679	145,0281	119,0487	
35	N1,N5,N10-Tricoumaroylspermidine isomer 3	C34H37N3O6	30,31		582,26042	462,2035	342,1468	316,1660	145,0282	119,0487	
36[1]	Luteolin (3',4',5,7-Tetrahydroxyflavone)	C15H10O6	30,32		285,03991	217,0494	199,0389	175,0393	151,0024	133,0281	
37	Rosmanol isomer	C20H26O5	30,54		345,17020	301,1809	283,1704				
38	Chrysoeriol-O-(acetylrhamnosyl)glucuronide	C30H32O17	31,16		663,15612	299,0561	298,0484	284,0328	283,0240	256,0364	
39	N1,N5,N10-Tricoumaroylspermidine isomer 4	C34H37N3O6	31,17		582,26042	462,2037	342,1455	316,1671	145,0281	119,0487	
40	Apigenin-O-coumaroylhexoside	C30H26O12	31,46		577,13461	431,0999	269,0454	268,0373	145,0284		
41	Rosmanol	C20H26O5	31,50		345,17020	301,1806	283,1704	268,1473			
42	Chrysoeriol-O-(p-coumaroyl)hexoside	C31H28O13	31,61		607,14517	461,1107	443,0985	299,0562	284,0327	145,0282	
43	Dimethoxy-trihydroxy(iso)flavone isomer 1	C17H14O7	31,88		329,06613	314,0437	299,0198	278,0670	271,0246	243,0289	
44[1]	Apigenin (4',5,7-Trihydroxyflavone)	C15H10O5	32,06		269,04500	227,0345	225,0552	151,0024	149,0231	117,0330	
45[1]	Chrysoeriol (3'-Methoxy-4',5,7-trihydroxyflavone)	C16H12O6	32,29		299,05556	284,0326	256,0376	227,0339	151,0017	107,0123	
46	Dimethoxy-trihydroxy(iso)flavone isomer 2	C17H14O7	32,34		329,06613	314,0432	313,0359	299,0196	271,0251	243,0294	
47	Traumatic acid (2-Dodecenedioic acid)	C12H20O4	33,45		227,12834	209,1178	183,1380	165,1272	111,0795		
48	Traumatic acid isomer	C12H20O4	33,92		227,12834	209,1179	183,1380	165,1272	111,0801		
49	Rosmanol isomer	C20H26O5	35,10		345,17020	301,1803	283,1702	268,1462			

50	Dihydroxy-methoxy(iso)flavone	C16H12O5	35,97		283,06065	268,0377					
51	Hydroxy-trimethoxy(iso)flavone	C18H16O6	36,64	329,10251		314,0783	313,0702	299,0547	296,0678	268,0728	
52	9-Hydroxyoctadecatrienoic acid	C18H30O3	40,78		293,21167	275,2018	231,2119	171,1015	121,1009	59,0121	
53	Stearidonic acid	C18H28O2	40,95		275,20111	231,2120	59,0122				
54	13-Hydroxyoctadecatrienoic acid	C18H30O3	40,96		293,21167	275,2020	235,1697	223,1332	195,1382	59,0121	
55	9-Hydroxyoctadecadienoic acid	C18H32O3	42,01		295,22732	277,2172	195,1382	171,1013	59,0121		
56[1]	α-Linolenic acid	C18H30O2	45,19		277,21676	233,1906					
57[1]	Linoleic acid	C18H32O2	46,11		279,23241						
58[1]	Oleic acid	C18H34O2	47,05		281,24806						
59[1]	Stearic acid	C18H36O2	48,08		283,26371						
60	Pheophytin A	C55H74N4O5	65,20	871,57375		593,2755	533,2545	505,2195	460,2247	433,2415	

[1] Confirmed by standard

Table S10. Chemical composition of the methanol extract of *P. kurdica*

No.	Name	Formula	Rt	[M + H]+	[M - H]-	Fragment 1	Fragment 2	Fragment 3	Fragment 4	Fragment 5	Literature
1	Quinic acid	C7H12O6	2,04		191,05557	173,0441	171,0286	127,0387	111,0438	85,0279	
2	Citric acid	C6H8O7	3,00		191,01918	173,0081	171,0282	111,0072	87,0071	85,0279	
3	Protocatechuic acid (3,4-Dihydroxybenzoic acid)	C7H6O4	9,00		153,01879	109,0280	108,0203	91,0169	81,0333		
4	Vanillic acid-4-O-glucoside	C14H18O9	11,91		329,08726	167,0337	152,0102	123,0436	108,0202		[8]
5	Hydroxybenzoic acid	C7H6O3	13,79		137,02387	94,0362	93,0330	65,0380			
6	Decaffeoylverbascoside	C20H30O12	14,54		461,16591	315,1093	297,0989	161,0445	135,0438	113,0229	
7	Hydroxybenzoic acid glucoside	C13H16O8	15,34		299,07670	137,0231	93,0330				
8	Vanillic acid (4-Hydroxy-3-methoxybenzoic acid)	C8H8O4	16,84		167,03444	152,0103	123,0437	108,0201			
9[1]	Chlorogenic acid (3-O-Caffeoylquinic acid)	C16H18O9	17,17	355,10291		163,0389	145,0285	135,0441	117,0337	89,0388	[8]
10[1]	Caffeic acid	C9H8O4	17,54		179,03444	135,0438	107,0486				
11[1]	Vanillin (4-Hydroxy-3-methoxybenzaldehyde)	C8H8O3	18,35	153,05517		125,0596	111,0444	110,0362	93,0338	65,0391	
12	Kynurenic acid	C10H7NO3	18,44	190,05042		162,0549	144,0440	116,0494	89,0390		
13	Benzyl-primeveroside or Icariside F2	C18H26O10	18,49		401,14478	269,1030	161,0443	149,0593	101,0229	71,0122	
14	Naringenin-6,8-di-C-glucoside	C27H32O15	18,76		595,16630	475,1226	415,1050	385,0927	355,0819	271,0609	
15	Ipolamiide	C17H26O11	18,79		405,13969	243,0872	225,0764	101,0229	71,0121	68,9965	
16	Benzyl-primeveroside or Icariside F2	C18H26O10	18,95		401,14478	269,1033	161,0445	113,0232	101,0229	71,0122	
17	Syringaldehyde (3,5-Dimethoxy-4-hydroxybenzaldehyde)	C9H10O4	19,76	183,06574		155,0703	140,0469	123,0443	105,0338	95,0497	
18	5-O-(p-Coumaroyl)quinic acid	C16H18O8	19,82		337,09235	191,0552	173,0444	163,0388	119,0487		
19	12-Hydroxyjasmonic acid or Tuberonic acid	C12H18O4	20,17		225,11269	181,1225	165,0906	163,1120	147,0799	59,0123	
20	12-Hydroxyjasmonic acid or Tuberonic acid	C12H18O4	20,42		225,11269	181,1226	165,0907	163,1121	147,0802	59,0123	
21	Caffeoylshikimic acid	C16H16O8	20,48		335,07670	179,0340	173,0441	161,0232	135,0438	111,0438	
22	5-O-Feruloylquinic acid	C17H20O9	20,66		367,10291	193,0502	191,0553	173,0445	134,0361		
23[1]	p-Coumaric acid	C9H8O3	20,74		163,03952	119,0487					
24	Vicenin-2 (Apigenin-6,8-di-C-glucoside)	C27H30O15	20,91	595,16630		577,1540	541,1340	457,1129	325,0704	295,0602	
25	5-O-(p-Coumaroyl)quinic acid cis isomer	C16H18O8	21,40		337,09235	191,0553	173,0442	163,0389	119,0485		
26[1]	Ferulic acid	C10H10O4	21,88		193,05009	178,0262	149,0594	137,0233	134,0360		
27	N1,N10-Bis(p-coumaroyl)spermidine	C25H31N3O4	22,16		436,22364	316,1668	290,1871	145,0279	119,0487		

28	Eriodictyol-O-hexoside	C21H22O11	22,23		449,10839	287,0562	151,0023	135,0438	107,0124		
29	12-Hydroxyjasmonic acid sulfate or Tuberonic acid sulfate	C12H18O7S	22,51		305,06950	225,1125	96,9586	59,0122			
30	Apigenin-C-hexoside-C-pentoside	C26H28O14	22,70	565,15574		547,1409	529,1352	379,0809	325,0706	295,0603	
31	Forsythoside B	C34H44O19	23,39		755,23986	593,2087	461,1651	447,1525	315,1100	161,0231	[9]
32	Luteolin-7-O-sophoroside	C27H30O16	23,52		609,14557	447,0918	285,0406	284,0331	133,0276		[8]
33	Verbascoside (Acteoside)	C29H36O15	23,74		623,19760	461,1665	315,1088	179,0338	161,0231	133,0282	[9]
34	Luteolin-O-rhamnosylglucuronide isomer 1	C27H28O16	24,17		607,12991	285,0406	284,0331	133,0276			
35	Luteolin-O-pentosylhexoside	C26H28O15	24,27		579,13500	285,0406	284,0330	151,0022	133,0280		
36	Prunin (Naringenin-7-O-glucoside)	C21H22O10	24,30		433,11347	271,0613	177,0185	151,0024	119,0487	107,0124	
37	Samioside	C34H44O19	24,48		755,23986	593,2092	461,1662	315,1097	179,0340	161,0231	
38	Hattushoside	C28H36O15	24,64		611,19760	431,1580	413,1464	299,1140	197,0448	153,0544	
39[1]	Luteolin-7-O-glucoside (Cynaroside)	C21H20O11	24,66		447,09274	285,0405	284,0327	256,0382	151,0024	133,0282	[8]
40	Luteolin-O-crotonylhexoside	C25H24O12	24,67		515,11896	447,0931	285,0404	284,0328			
41	Alyssonoside	C35H46O19	24,86		769,25551	593,2090	575,1982	461,1664	447,1502	175,0389	[9]
42	Forsythoside B isomer	C34H44O19	25,08		755,23986	593,2091	461,1665	447,1506	315,1092	161,0231	
43	Isoverbascoside	C29H36O15	25,10		623,19760	461,1669	315,1084	179,0341	161,0231	135,0439	
44	Luteolin-O-glucuronide	C21H18O12	25,32		461,07201	285,0405	199,0390	175,0387	151,0022	133,0281	
45	Luteolin-7-O-(6'''-O-acetylglucosyl)-(1→2)glucoside	C29H32O17	25,40		651,15613	285,0407	284,0326	133,0276			[8]
46	Leucosceptoside A	C30H38O15	25,45		637,21325	461,1668	443,1552	315,1079	175,0389	160,0153	[9]
47	Luteolin-O-rhamnosylglucuronide isomer 2	C27H28O16	25,46		607,12991	285,0406	284,0338	151,0025	133,0279		
48	Caffeoyl-vanilloylglucose	C23H24O12	25,64		491,11896	323,0772	179,0338	167,0337	161,0231	152,0102	
49	Chrysoeriol-O-pentosylhexoside	C27H30O15	25,96		593,15065	299,0560	297,0388	285,0403	284,0327		
50	Alyssonoside isomer	C35H46O19	26,13		769,25551	593,2092	575,1987	461,1663	315,1107	175,0389	
51[1]	Cosmosiin (Apigenin-7-O-glucoside)	C21H20O10	26,23	433,11347		271,0597	153,0177	119,0494			
52	Rosmarinic acid (Labiatenic acid)	C18H16O8	26,34		359,07670	197,0449	179,0341	161,0232	135,0439	72,9915	
53	Chrysoeriol-7-O-glucoside	C22H22O11	26,52		461,10839	446,0856	299,0562	298,0483	283,0249	255,0296	
54	Leucosceptoside B isomer	C36H48O19	26,89		783,27116	607,2247	589,2133	461,1671	193,0499	175,0389	
55[1]	Eriodictyol (3′,4′,5,7-Tetrahydroxyflavanone)	C15H12O6	26,95		287,05556	269,0501	151,0024	135,0439	107,0124	83,0121	
56[1]	Apigenin-7-O-glucuronide	C21H18O11	27,04		445,07709	269,0457	175,0236	113,0229			
57	Caffeoyl-hydroxybenzoylhexose	C22H22O11	27,08		461,10839	323,0772	283,0249	179,0341	161,0232	137,0231	
58	Methoxy-tetrahydroxy(iso)flavone-O-glucuronide	C22H20O13	27,09		491,08257	315,0513	300,0278	269,0452	137,0232	113,0230	
59	Chrysoeriol-O-glucuronide	C22H20O12	27,24		475,08766	299,0561	284,0328	256,0370			
60	Abscisic acid	C15H20O4	27,27		263,12834	219,1384	204,1147	201,1265	152,0831	151,0750	

61	Chrysoeriol-O-(acetylglucosyl)glucuronide	C30H32O18	27,44		679,15104	379,0881	299,0561	284,0327	256,0372	113,0229	
62	Martynoside	C31H40O15	27,68		651,22890	475,1822	329,1246	193,0500	175,0389	160,0153	
63	Leucosceptoside B	C36H48O19	28,07		783,27116	607,2244	589,2127	475,1843	329,1262	175,0390	[9]
64	N1,N5,N10-Tricoumaroylspermidine isomer 1	C34H37N3O6	28,43		582,26042	462,2041	342,1466	316,1654	145,0283	119,0487	
65[1]	Naringenin (4',5,7-Trihydroxyflavanone)	C15H12O5	29,23		271,06065	227,0701	177,0184	151,0024	119,0487	107,0124	
66	Homoeriodictyol (3'-Methoxy-4',5,7-trihydroxyflavanone)	C16H14O6	29,29		301,07122	196,0000	177,0184	151,0024	149,0596	134,0359	
67	N1,N5,N10-Tricoumaroylspermidine isomer 2	C34H37N3O6	29,42		582,26042	462,2040	342,1456	316,1668	145,0281	119,0487	
68	Luteolin-O-(acetylrhamnosyl)glucuronide	C29H30O17	29,72		649,14048	285,0404	284,0322	199,0390	151,0022	133,0282	
69	N1,N5,N10-Tricoumaroylspermidine isomer 3	C34H37N3O6	30,29		582,26042	462,2032	342,1459	316,1676	145,0282	119,0487	
70[1]	Luteolin (3',4',5,7-Tetrahydroxyflavone)	C15H10O6	30,30		285,03991	217,0497	199,0393	175,0390	151,0024	133,0281	
71	Luteolin-O-(p-coumaroyl)hexoside	C30H26O13	30,39		593,12952	447,0904	429,0828	285,0405	284,0325	145,0282	
72	Rosmanol isomer	C20H26O5	30,53		345,17020	301,1810	283,1703				
73	Apigenin-O-(acetylrhamnosyl)glucuronide	C29H30O16	31,13		633,14556	269,0457	268,0386	227,0340	225,0548	113,0229	
74	Chrysoeriol-O-(acetylrhamnosyl)glucuronide	C30H32O17	31,14		663,15612	299,0560	298,0485	284,0327	283,0240	256,0367	
75	N1,N5,N10-Tricoumaroylspermidine isomer 4	C34H37N3O6	31,17		582,26042	462,2038	342,1460	316,1669	145,0283	119,0487	
76	Apigenin-O-coumaroylhexoside	C30H26O12	31,45		577,13461	431,0979	269,0456	268,0376	145,0282		
77	Rosmanol	C20H26O5	31,50		345,17020	301,1798	283,1704	268,1467			
78	Chrysoeriol-O-(p-coumaroyl)hexoside	C31H28O13	31,62		607,14517	461,1090	443,0986	299,0560	284,0327	145,0282	
79	Dimethoxy-trihydroxy(iso)flavone isomer 1	C17H14O7	31,91		329,06613	314,0432	299,0200	278,0671	271,0246	243,0277	
80[1]	Apigenin (4',5,7-Trihydroxyflavone)	C15H10O5	32,05		269,04500	227,0333	225,0552	151,0024	149,0231	117,0330	
81[1]	Chrysoeriol (3'-Methoxy-4',5,7-trihydroxyflavone)	C16H12O6	32,29		299,05556	284,0327	256,0374	227,0351	151,0018	107,0123	
82	Dimethoxy-trihydroxy(iso)flavone isomer 2	C17H14O7	32,32		329,06613	314,0435	313,0349	299,0197	271,0252	243,0300	
83	Traumatic acid (2-Dodecenedioic acid)	C12H20O4	33,46		227,12834	209,1181	183,1381	165,1272	111,0797		
84	Traumatic acid isomer	C12H20O4	33,92		227,12834	209,1182	183,1381	165,1273	111,0801		
85	Rosmanol isomer	C20H26O5	35,11		345,17020	301,1829	283,1704	268,1468			
86	Dihydroxy-methoxy(iso)flavone	C16H12O5	35,97		283,06065	268,0377					
87	Hydroxy-trimethoxy(iso)flavone	C18H16O6	36,66	329,10251		314,0783	313,0703	299,0551	296,0680	268,0726	
88	9-Hydroxyoctadecatrienoic acid	C18H30O3	40,78		293,21167	275,2019	231,2111	171,1015	121,1008	59,0123	
89	13-Hydroxyoctadecatrienoic acid	C18H30O3	40,96		293,21167	275,2019	235,1703	223,1335	195,1385	59,0122	
90	9-Hydroxyoctadecadienoic acid	C18H32O3	42,00		295,22732	277,2174	195,1383	171,1014	59,0124		
91[1]	α-Linolenic acid	C18H30O2	45,20		277,21676	233,1904					
92[1]	Linoleic acid	C18H32O2	46,11		279,23241						
93	Pheophytin A	C55H74N4O5	64,95	871,57375		593,2763	533,2554	505,2209	460,2256	433,2405	

[1] Confirmed by standard

Table S11. Chemical composition of the methanol/water extract of *P. kurdica*

No.	Name	Formula	Rt	$[M + H]^+$	$[M - H]^-$	Fragment 1	Fragment 2	Fragment 3	Fragment 4	Fragment 5	Literature
1	Quinic acid	C7H12O6	2,03		191,05557	173,0441	171,0285	127,0388	111,0436	85,0279	
2	Citric acid	C6H8O7	2,94		191,01918	173,0080	171,0287	111,0073	87,0072	85,0279	
3	Protocatechuic acid (3,4-Dihydroxybenzoic acid)	C7H6O4	8,98		153,01879	109,0280	108,0202	91,0173	81,0331		
4	Vanillic acid-4-O-glucoside	C14H18O9	11,89		329,08726	167,0338	152,0102	123,0437	108,0202		[8]
5	Hydroxybenzoic acid	C7H6O3	13,80		137,02387	94,0367	93,0330	65,0381			
6	Decaffeoylverbascoside	C20H30O12	14,52		461,16591	315,1091	297,0987	161,0443	135,0438	113,0229	
7	Hydroxybenzoic acid glucoside	C13H16O8	15,34		299,07670	137,0230	93,0330				
8	Vanillic acid (4-Hydroxy-3-methoxybenzoic acid)	C8H8O4	16,82		167,03444	152,0102	123,0436	108,0202			
9[1]	Chlorogenic acid (3-O-Caffeoylquinic acid)	C16H18O9	17,25	355,10291		163,0387	145,0284	135,0441	117,0335	89,0388	[8]
10[1]	Caffeic acid	C9H8O4	17,55		179,03444	135,0438	107,0486				
11[1]	Vanillin (4-Hydroxy-3-methoxybenzaldehyde)	C8H8O3	18,37	153,05517		125,0598	111,0443	110,0366	93,0339	65,0392	
12	Benzyl-primeveroside or Icariside F2	C18H26O10	18,49		401,14478	269,1029	161,0447	149,0596	101,0230	71,0122	
13	Kynurenic acid	C10H7NO3	18,67	190,05042		162,0548	144,0437	116,0496	89,0390		
14	Naringenin-6,8-di-C-glucoside	C27H32O15	18,74		595,16630	475,1234	415,1050	385,0939	355,0822	271,0607	
15	Ipolamiide	C17H26O11	18,79		405,13969	243,0871	225,0762	101,0230	71,0120	68,9965	
16	Benzyl-primeveroside or Icariside F2	C18H26O10	18,95		401,14478	269,1033	161,0440	113,0232	101,0230	71,0122	
17	Syringaldehyde (3,5-Dimethoxy-4-hydroxybenzaldehyde)	C9H10O4	19,78	183,06574		155,0701	140,0468	123,0442	105,0338	95,0496	
18	5-O-(p-Coumaroyl)quinic acid	C16H18O8	19,83		337,09235	191,0552	173,0443	163,0388	119,0487		
19	12-Hydroxyjasmonic acid or Tuberonic acid	C12H18O4	20,17		225,11269	181,1223	165,0906	163,1118	147,0798	59,0123	
20	12-Hydroxyjasmonic acid or Tuberonic acid	C12H18O4	20,41		225,11269	181,1233	165,0906	163,1115	147,0803	59,0123	
21	Caffeoylshikimic acid	C16H16O8	20,47		335,07670	179,0340	173,0446	161,0232	135,0438	111,0436	
22	5-O-Feruloylquinic acid	C17H20O9	20,66		367,10291	193,0499	191,0553	173,0445	134,0360		
23[1]	p-Coumaric acid	C9H8O3	20,73		163,03952	119,0487					
24	Vicenin-2 (Apigenin-6,8-di-C-glucoside)	C27H30O15	20,91	595,16630		577,1543	541,1342	457,1129	325,0705	295,0603	
25	5-O-(p-Coumaroyl)quinic acid cis isomer	C16H18O8	21,40		337,09235	191,0552	173,0443	163,0391	119,0486		
26[1]	Ferulic acid	C10H10O4	21,88		193,05009	178,0262	149,0597	137,0232	134,0361		

27	N1,N10-Bis(p-coumaroyl)spermidine	C25H31N3O4	22,14		436,22364	316,1670	290,1876	145,0284	119,0487		
28	Eriodictyol-O-hexoside	C21H22O11	22,22		449,10839	287,0562	151,0024	135,0438	107,0123		
29	12-Hydroxyjasmonic acid sulfate or Tuberonic acid sulfate	C12H18O7S	22,52		305,06950	225,1133	96,9585	59,0123			
30	Apigenin-C-hexoside-C-pentoside	C26H28O14	22,71	565,15574		547,1407	529,1350	379,0804	325,0705	295,0601	
31	Forsythoside B	C34H44O19	23,38		755,23986	593,2084	461,1684	447,1505	315,1095	161,0232	[9]
32	Luteolin-7-O-sophoroside	C27H30O16	23,49		609,14557	447,0936	285,0405	284,0328	133,0279		[8]
33	Luteolin-O-hexosylglucuronide	C27H28O17	23,60		623,12483	285,0406	284,0332	151,0018	133,0282		
34	Verbascoside (Acteoside)	C29H36O15	23,73		623,19760	461,1671	315,1080	179,0340	161,0231	133,0282	[9]
35	Luteolin-O-rhamnosylglucuronide isomer 1	C27H28O16	24,15		607,12991	285,0406	284,0330	133,0274			
36	Luteolin-O-pentosylhexoside	C26H28O15	24,25		579,13500	285,0406	284,0331	151,0024	133,0282		
37	Prunin (Naringenin-7-O-glucoside)	C21H22O10	24,26		433,11347	271,0613	177,0185	151,0024	119,0487	107,0125	
38	Samioside	C34H44O19	24,45		755,23986	593,2083	461,1661	315,1084	179,0337	161,0231	
39	Hattushoside	C28H36O15	24,61		611,19760	431,1563	413,1446	299,1142	197,0448	153,0544	
40[1]	Luteolin-7-O-glucoside (Cynaroside)	C21H20O11	24,63		447,09274	285,0405	284,0328	256,0381	151,0019	133,0276	[8]
41	Luteolin-O-crotonylhexoside	C25H24O12	24,65		515,11896	447,0933	285,0407	284,0328	133,0283		
42	Alyssonoside	C35H46O19	24,84		769,25551	593,2092	575,1981	461,1662	447,1521	175,0389	[9]
43	Forsythoside B isomer	C34H44O19	25,05		755,23986	593,2092	461,1685	447,1532	315,1083	161,0232	
44	Isoverbascoside	C29H36O15	25,08		623,19760	461,1662	315,1080	179,0341	161,0231	135,0438	
45	Luteolin-O-glucuronide	C21H18O12	25,29		461,07201	285,0405	199,0386	175,0391	151,0025	133,0280	
46	Luteolin-7-O-(6'''-O-acetylglucosyl)-(1→2)glucoside	C29H32O17	25,39		651,15613	285,0405	284,0327	133,0284			[8]
47	Leucosceptoside A	C30H38O15	25,42		637,21325	461,1667	443,1511	315,1090	175,0389	160,0153	[9]
48	Luteolin-O-rhamnosylglucuronide isomer 2	C27H28O16	25,44		607,12991	285,0406	284,0331	151,0020	133,0283		
49	Caffeoyl-vanilloylglucose	C23H24O12	25,62		491,11896	323,0772	179,0340	167,0338	161,0231	152,0103	
50	Chrysoeriol-O-pentosylhexoside	C27H30O15	25,94		593,15065	299,0562	297,0408	285,0418	284,0328	255,0300	
51	Alyssonoside isomer	C35H46O19	26,12		769,25551	593,2083	575,1963	461,1652	315,1113	175,0389	
52[1]	Cosmosiin (Apigenin-7-O-glucoside)	C21H20O10	26,24	433,11347		271,0599	153,0179	119,0494			
53	Rosmarinic acid (Labiatenic acid)	C18H16O8	26,32		359,07670	197,0448	179,0341	161,0231	135,0438	72,9915	
54	Chrysoeriol-7-O-glucoside	C22H22O11	26,49		461,10839	446,0855	299,0557	298,0481	283,0248	255,0296	
55	Leucosceptoside B isomer	C36H48O19	26,87		783,27116	607,2244	589,2135	461,1672	193,0499	175,0389	
56[1]	Eriodictyol (3′,4′,5,7-Tetrahydroxyflavanone)	C15H12O6	26,93		287,05556	269,0500	151,0023	135,0438	107,0123	83,0123	
57[1]	Apigenin-7-O-glucuronide	C21H18O11	27,02		445,07709	269,0455	175,0237	113,0227			
58	Caffeoyl-hydroxybenzoylhexose	C22H22O11	27,07		461,10839	323,0772	283,0252	179,0340	161,0231	137,0231	
59	Methoxy-tetrahydroxy(iso)flavone-O-glucuronide	C22H20O13	27,08		491,08257	315,0512	300,0275	269,0469	137,0231	113,0229	

60	Chrysoeriol-O-glucuronide	C22H20O12	27,23		475,08766	299,0561	284,0328	256,0382	113,0229		
61	Abscisic acid	C15H20O4	27,25		263,12834	219,1379	204,1149	201,1280	152,0830	151,0750	
62	Chrysoeriol-O-(acetylglucosyl)glucuronide	C30H32O18	27,42		679,15104	379,0879	299,0561	284,0327	256,0378	113,0229	
63	Martynoside	C31H40O15	27,65		651,22890	475,1817	329,1244	193,0497	175,0389	160,0153	
64	Leucosceptoside B	C36H48O19	28,05		783,27116	607,2274	589,2129	475,1843	329,1261	175,0390	[9]
65	N1,N5,N10-Tricoumaroylspermidine isomer 1	C34H37N3O6	28,41		582,26042	462,2029	342,1455	316,1671	145,0284	119,0487	
66[1]	Naringenin (4',5,7-Trihydroxyflavanone)	C15H12O5	29,22		271,06065	227,0707	177,0183	151,0024	119,0487	107,0123	
67	Homoeriodictyol (3'-Methoxy-4',5,7-trihydroxyflavanone)	C16H14O6	29,26		301,07122	196,0002	177,0181	151,0022	149,0590	134,0359	
68	N1,N5,N10-Tricoumaroylspermidine isomer 2	C34H37N3O6	29,41		582,26042	462,2040	342,1455	316,1671	145,0282	119,0487	
69	Luteolin-O-(acetylrhamnosyl)glucuronide	C29H30O17	29,70		649,14048	285,0404	284,0326	199,0389	151,0022	133,0227	
70[1]	Luteolin (3',4',5,7-Tetrahydroxyflavone)	C15H10O6	30,27		285,03991	217,0496	199,0395	175,0391	151,0025	133,0282	
71	N1,N5,N10-Tricoumaroylspermidine isomer 3	C34H37N3O6	30,30		582,26042	462,2041	342,1465	316,1665	145,0282	119,0487	
72	Luteolin-O-(p-coumaroyl)hexoside	C30H26O13	30,38		593,12952	447,0936	429,0819	285,0404	284,0328	145,0281	
73	Rosmanol isomer	C20H26O5	30,51		345,17020	301,1810	283,1702				
74	Apigenin-O-(acetylrhamnosyl)glucuronide	C29H30O16	31,12		633,14556	269,0455	268,0372	227,0329	225,0546	113,0228	
75	Chrysoeriol-O-(acetylrhamnosyl)glucuronide	C30H32O17	31,13		663,15612	299,0561	298,0489	284,0327	283,0252	256,0384	
76	N1,N5,N10-Tricoumaroylspermidine isomer 4	C34H37N3O6	31,15		582,26042	462,2035	342,1455	316,1664	145,0282	119,0487	
77	Apigenin-O-(p-coumaroyl)hexoside	C30H26O12	31,43		577,13461	431,0979	269,0456	268,0378	145,0282		
78	Rosmanol	C20H26O5	31,47		345,17020	301,1799	283,1704	268,1468			
79	Chrysoeriol-O-(p-coumaroyl)hexoside	C31H28O13	31,59		607,14517	461,1096	443,0986	299,0560	284,0327	145,0282	
80	Dimethoxy-trihydroxy(iso)flavone isomer 1	C17H14O7	31,90		329,06613	314,0436	299,0197	278,0670	271,0244	243,0274	
81[1]	Apigenin (4',5,7-Trihydroxyflavone)	C15H10O5	32,04		269,04500	227,0340	225,0550	151,0024	149,0231	117,0330	
82[1]	Chrysoeriol (3'-Methoxy-4',5,7-trihydroxyflavone)	C16H12O6	32,27		299,05556	284,0327	256,0374	227,0343	151,0024	107,0124	
83	Dimethoxy-trihydroxy(iso)flavone isomer 2	C17H14O7	32,30		329,06613	314,0434	313,0367	299,0196	271,0244	243,0301	
84	Traumatic acid (2-Dodecenedioic acid)	C12H20O4	33,43		227,12834	209,1174	183,1381	165,1272	111,0799		
85	Traumatic acid isomer	C12H20O4	33,90		227,12834	209,1178	183,1381	165,1273	111,0800		
86	Rosmanol isomer	C20H26O5	35,09		345,17020	301,1832	283,1706	268,1469			
87	Dihydroxy-methoxy(iso)flavone	C16H12O5	35,95		283,06065	268,0379					
88	Hydroxy-trimethoxy(iso)flavone	C18H16O6	36,64	329,10251		314,0780	313,0702	299,0550	296,0678	268,0727	
89	9-Hydroxyoctadecatrienoic acid	C18H30O3	40,78		293,21167	275,2018	231,2119	171,1013	121,1007	59,0122	
90	13-Hydroxyoctadecatrienoic acid	C18H30O3	40,96		293,21167	275,2023	235,1698	223,1330	195,1385	59,0122	
91	9-Hydroxyoctadecadienoic acid	C18H32O3	42,00		295,22732	277,2174	195,1383	171,1015	59,0122		

92[1]	α-Linolenic acid	C18H30O2	45,19		277,21676	233,1897					
93[1]	Linoleic acid	C18H32O2	46,10		279,23241						

[1] Confirmed by standard

Table S12. Chemical composition of the water extract of *P. kurdica*

No.	Name	Formula	Rt	$[M + H]^+$	$[M - H]^-$	Fragment 1	Fragment 2	Fragment 3	Fragment 4	Fragment 5	Literature
1	Quinic acid	C7H12O6	2,03		191,05557	173,0446	171,0283	127,0387	111,0436	85,0279	
2	Citric acid	C6H8O7	2,95		191,01918	173,0081	171,0284	111,0073	87,0072	85,0279	
3	Protocatechuic acid (3,4-Dihydroxybenzoic acid)	C7H6O4	9,03		153,01879	109,0280	108,0202	91,0173	81,0330		
4	Vanillic acid-4-O-glucoside	C14H18O9	11,90		329,08726	167,0338	152,0102	123,0437	108,0202		[8]
5	Hydroxybenzoic acid glucoside	C13H16O8	15,35		299,07670	137,0231	93,0330				
6	Vanillic acid (4-Hydroxy-3-methoxybenzoic acid)	C8H8O4	16,85		167,03444	152,0103	123,0433	108,0201			
7[1]	Chlorogenic acid (3-O-Caffeoylquinic acid)	C16H18O9	17,25	355,10291		163,0389	145,0284	135,0442	117,0337	89,0388	[8]
8[1]	Caffeic acid	C9H8O4	17,55		179,03444	135,0438	107,0488				
9[1]	Vanillin (4-Hydroxy-3-methoxybenzaldehyde)	C8H8O3	18,36	153,05517		125,0599	111,0443	110,0365	93,0339	65,0392	
10	Benzyl-primeveroside or Icariside F2	C18H26O10	18,49		401,14478	269,1040	161,0442	149,0594	101,0229	71,0123	
11	Kynurenic acid	C10H7NO3	18,69	190,05042		162,0549	144,0445	116,0497	89,0390		
12	Naringenin-6,8-di-C-glucoside	C27H32O15	18,75		595,16630	475,1233	415,1014	385,0923	355,0822	271,0607	
13	Ipolamiide	C17H26O11	18,79		405,13969	243,0870	225,0759	101,0229	71,0122	68,9966	
14	Benzyl-primeveroside or Icariside F2	C18H26O10	18,95		401,14478	269,1033	161,0443	113,0228	101,0228	71,0122	
15	Syringaldehyde (3,5-Dimethoxy-4-hydroxybenzaldehyde)	C9H10O4	19,79	183,06574		155,0703	140,0468	123,0441	105,0338	95,0495	
16	5-O-(p-Coumaroyl)quinic acid	C16H18O8	19,84		337,09235	191,0553	173,0445	163,0389	119,0488		
17	12-Hydroxyjasmonic acid or Tuberonic acid	C12H18O4	20,17		225,11269	181,1222	165,0906	163,1112	147,0801	59,0123	
18	12-Hydroxyjasmonic acid or Tuberonic acid	C12H18O4	20,40		225,11269	181,1219	165,0908	163,1118	147,0799	59,0122	
19	Caffeoylshikimic acid	C16H16O8	20,48		335,07670	179,0341	173,0445	161,0232	135,0438	111,0436	
20	Riboflavin	C17H20N4O6	20,51	377,14611		359,1337	243,0875	200,0818	172,0870	69,0341	
21	5-O-Feruloylquinic acid	C17H20O9	20,66		367,10291	193,0502	191,0553	173,0446	134,0362		

22[1]	p-Coumaric acid	C9H8O3	20,73		163,03952	119,0487					
23	Vicenin-2 (Apigenin-6,8-di-C-glucoside)	C27H30O15	20,92	595,16630		577,1538	541,1353	457,1131	325,0705	295,0603	
24	5-O-(p-Coumaroyl)quinic acid cis isomer	C16H18O8	21,40		337,09235	191,0553	173,0435	163,0386	119,0487		
25	12-Hydroxyjasmonic acid sulfate or Tuberonic acid sulfate	C12H18O7S	22,52		305,06950	225,1126	96,9587	59,0123			
26	Apigenin-C-hexoside-C-pentoside	C26H28O14	22,70	565,15574		547,1409	529,1331	379,0803	325,0703	295,0597	
27	Forsythoside B	C34H44O19	23,39		755,23986	593,2121	461,1656	447,1529	315,1104	161,0231	[9]
28	Luteolin-7-O-sophoroside	C27H30O16	23,49		609,14557	447,0926	285,0407	284,0325	133,0275		[8]
29	Luteolin-O-hexosylglucuronide	C27H28O17	23,68		623,12483	285,0407	284,0324	151,0025	133,0282		
30	Luteolin-O-rhamnosylglucuronide isomer 1	C27H28O16	24,19		607,12991	285,0407	284,0330	133,0274			
31	Luteolin-O-pentosylhexoside	C26H28O15	24,26		579,13500	285,0407	284,0320	151,0027	133,0284		
32	Hattushoside	C28H36O15	24,64		611,19760	431,1575	413,1449	299,1133	197,0448	153,0545	
33[1]	Luteolin-7-O-glucoside (Cynaroside)	C21H20O11	24,65		447,09274	285,0408	284,0326	256,0380	151,0021	133,0282	[8]
34	Luteolin-O-crotonylhexoside	C25H24O12	24,68		515,11896	447,0931	285,0409	284,0331			
35	Alyssonoside	C35H46O19	24,91		769,25551	593,2065	575,1980	461,1661	447,1498	175,0391	[9]
36	Luteolin-O-glucuronide	C21H18O12	25,33		461,07201	285,0406	199,0400	175,0388	151,0024	133,0280	
37	Luteolin-7-O-(6'''-O-acetylglucosyl)-(1→2)glucoside	C29H32O17	25,39		651,15613	285,0406	284,0321	133,0275			[8]
38	Luteolin-O-rhamnosylglucuronide isomer 2	C27H28O16	25,46		607,12991	285,0407	284,0336	151,0021	133,0279		
39	Caffeoyl-vanilloylglucose	C23H24O12	25,63		491,11896	323,0774	179,0338	167,0337	161,0235	152,0100	
40	Chrysoeriol-O-pentosylhexoside	C27H30O15	25,95		593,15065	299,0564	297,0389	285,0407	284,0330		
41	Rosmarinic acid (Labiatenic acid)	C18H16O8	26,33		359,07670	197,0450	179,0331	161,0232	135,0437	72,9916	
42	Chrysoeriol-7-O-glucoside	C22H22O11	26,49		461,10839	446,0859	299,0563	298,0483	283,0250	255,0296	
43	Leucosceptoside B isomer	C36H48O19	26,88		783,27116	607,2257	589,2139	461,1670	193,0499	175,0390	
44[1]	Eriodictyol (3′,4′,5,7-Tetrahydroxyflavanone)	C15H12O6	26,94		287,05556	269,0504	151,0024	135,0439	107,0126	83,0119	
45[1]	Apigenin-7-O-glucuronide	C21H18O11	27,05		445,07709	269,0456	175,0241	113,0229			
46	Caffeoyl-hydroxybenzoylhexose	C22H22O11	27,07		461,10839	323,0773	283,0250	179,0340	161,0231	137,0231	
47	Chrysoeriol-O-glucuronide	C22H20O12	27,24		475,08766	299,0562	284,0328	256,0366	113,0229		
48	Chrysoeriol-O-(acetylglucosyl)glucuronide	C30H32O18	27,43		679,15104	379,0881	299,0561	284,0328	256,0380	113,0229	
49	Martynoside	C31H40O15	27,65		651,22890	475,1824	329,1249	193,0501	175,0390	160,0153	

50	Leucosceptoside B	C36H48O19	28,05		783,27116	607,2256	589,2119	475,1840	329,1260	175,0389	[9]
51	N1,N5,N10-Tricoumaroylspermidine isomer 1	C34H37N3O6	28,41		582,26042	462,2032	342,1429	316,1691	145,0279	119,0488	
52[1]	Naringenin (4',5,7-Trihydroxyflavanone)	C15H12O5	29,21		271,06065	227,0707	177,0182	151,0024	119,0488	107,0124	
53	Homoeriodictyol (3'-Methoxy-4',5,7-trihydroxyflavanone)	C16H14O6	29,26		301,07122	196,0008	177,0183	151,0025	149,0596	134,0363	
54	N1,N5,N10-Tricoumaroylspermidine isomer 2	C34H37N3O6	29,40		582,26042	462,2063	342,1468	316,1669	145,0286	119,0488	
55	Luteolin-O-(acetylrhamnosyl)glucuronide	C29H30O17	29,72		649,14048	285,0406	284,0327	199,0400	151,0023	133,0279	
56	N1,N5,N10-Tricoumaroylspermidine isomer 3	C34H37N3O6	30,26		582,26042	462,2039	342,1464	316,1678	145,0285	119,0488	
57[1]	Luteolin (3',4',5,7-Tetrahydroxyflavone)	C15H10O6	30,28		285,03991	217,0498	199,0396	175,0389	151,0024	133,0282	
58	Apigenin-O-(acetylrhamnosyl)glucuronide	C29H30O16	31,14		633,14556	269,0458	268,0389	227,0344	225,0555	113,0230	
59	Chrysoeriol-O-(acetylrhamnosyl)glucuronide	C30H32O17	31,15		663,15612	299,0562	298,0479	284,0329	283,0262	256,0377	
60	N1,N5,N10-Tricoumaroylspermidine isomer 4	C34H37N3O6	31,16		582,26042	462,2030	342,1485	316,1673	145,0279	119,0487	
61	Chrysoeriol-O-(p-coumaroyl)hexoside	C31H28O13	31,59		607,14517	461,1088	443,0974	299,0562	284,0325	145,0283	
62[1]	Apigenin (4',5,7-Trihydroxyflavone)	C15H10O5	32,03		269,04500	227,0342	225,0552	151,0025	149,0231	117,0331	
63[1]	Chrysoeriol (3'-Methoxy-4',5,7-trihydroxyflavone)	C16H12O6	32,27		299,05556	284,0329	256,0377	227,0342	151,0020	107,0128	
64	Dimethoxy-trihydroxy(iso)flavone isomer 2	C17H14O7	32,32		329,06613	314,0432	313,0344	299,0186	271,0252	243,0298	

[1] Confirmed by standard

References

[1] S. Uysal, G. Zengin, M. Locatelli, M. B. Bahadori, A. Mocan, G. Bellagamba, E. De Luca, A. Mollica, A. Aktumsek, 'Cytotoxic and enzyme inhibitory potential of two Potentilla species (P. speciosa L. and P. reptans Willd.) and their chemical composition', *Frontiers in pharmacology* **2017**, *8*, 290.

[2] D. M. Grochowski, S. Uysal, A. Aktumsek, S. Granica, G. Zengin, R. Ceylan, M. Locatelli, M. Tomczyk, 'In vitro enzyme inhibitory properties, antioxidant activities, and phytochemical profile of Potentilla thuringiaca', *Phytochemistry Letters* **2017**, *20*, 365-372.

[3] G. Zengin, A. Uysal, A. Diuzheva, E. Gunes, J. Jekő, Z. Cziáky, C. M. N. Picot-Allain, M. F. Mahomoodally, 'Characterization of phytochemical components of Ferula halophila extracts using HPLC-MS/MS and their pharmacological potentials: A multi-functional insight', *Journal of Pharmaceutical and Biomedical Analysis* **2018**, *160*, 374-382.

[4] D. Stojković, U. Gašić, D. Drakulić, G. Zengin, M. Stevanović, N. Rajčević, M. Soković, 'Chemical profiling, antimicrobial, anti-enzymatic, and cytotoxic properties of Phlomis fruticosa L', *Journal of Pharmaceutical and Biomedical Analysis* **2021**, *195*, 113884.

[5] T. ERSÖZ, İ. SARACOĞLU, Ü. Ş. HARPUT, İ. ÇALIŞ, A. A. DÖNMEZ, 'Iridoid and phenylpropanoid glycosides from Phlomis grandiflora var. fimbrilligera and Phlomis fruticosa', *Turkish Journal of Chemistry* **2002**, *26*, 171-177.

[6] P. D. Marin, N. C. Veitch, R. J. Grayer, G. C. Kite, M. Soković, P. Janaćković, 'Flavonoids from Phlomis fruticosa (Lamiaceae) growing in Montenegro', *Biochemical Systematics and Ecology* **2007**, *35*, 462-466.
[7] İ. Saracoglu, K. KOJIMA, U. S. HARPUT, Y. OGIHARA, 'A new phenylethanoid glycoside from Phlomis pungens Willd. var. pungens', *Chemical and pharmaceutical bulletin* **1998**, *46*, 726-727.
[8] A. Bader, T. Tuccinardi, C. Granchi, A. Martinelli, M. Macchia, F. Minutolo, N. De Tommasi, A. Braca, 'Phenylpropanoids and flavonoids from Phlomis kurdica as inhibitors of human lactate dehydrogenase', *Phytochemistry* **2015**, *116*, 262-268.
[9] H. Kırmızıbekmez, P. Montoro, S. Piacente, C. Pizza, A. Dönmez, İ. Çalış, 'Identification by HPLC-PAD-MS and quantification by HPLC-PAD of phenylethanoid glycosides of five Phlomis species', *Phytochemical Analysis: An International Journal of Plant Chemical and Biochemical Techniques* **2005**, *16*, 1-6.
